# Photoswitching of Local (Anti)Aromaticity in Biphenylene-Based Diarylethene Molecular Switches

**DOI:** 10.1021/acs.joc.2c00504

**Published:** 2022-07-18

**Authors:** Péter Pál Kalapos, Péter J. Mayer, Tamás Gazdag, Attila Demeter, Baswanth Oruganti, Bo Durbeej, Gábor London

**Affiliations:** † MTA TTK Lendület Functional Organic Materials Research Group, Institute of Organic Chemistry, Research Centre for Natural Sciences, Magyar tudósok krt. 2, 1117 Budapest, Hungary; ‡ Institute of Chemistry, University of Szeged, Rerrich tér 1, 6720 Szeged, Hungary; § Hevesy György PhD School of Chemistry, Eötvös Loránd University, Pázmány Péter sétány 1/a, Budapest 1117, Hungary; ∥ Institute of Materials and Environmental Chemistry, Research Centre for Natural Sciences, Magyar tudósok krt. 2, 1117 Budapest, Hungary; ⊥ Department of Chemistry and Biomedical Sciences, Faculty of Health and Life Sciences, Linnaeus University, SE-45041 Kalmar, Sweden; # Division of Theoretical Chemistry, IFM, Linköping University, SE-58183 Linköping, Sweden

## Abstract

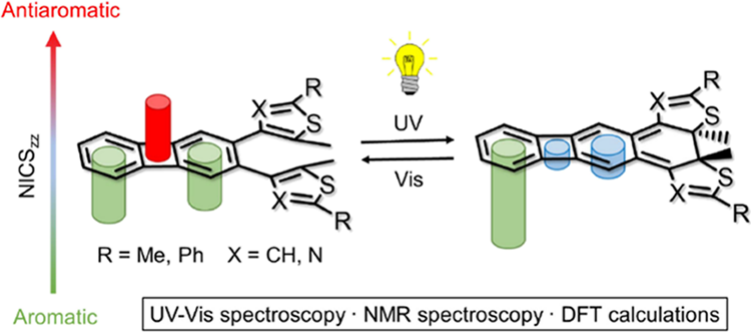

Photoinduced tuning of (anti)aromaticity and associated molecular properties is currently in the focus of attention for both tailoring photochemical reactivity and designing new materials. Here, we report on the synthesis and spectroscopic characterization of diarylethene-based molecular switches embedded in a biphenylene structure composed of rings with different levels of local (anti)aromaticity. We show that it is possible to modulate and control the (anti)aromatic character of each ring through reversible photoswitching of the aryl units of the system between open and closed forms. Remarkably, it is shown that the irreversible formation of an annulated bis(dihydro-thiopyran) side-product that hampers the photoswitching can be efficiently suppressed when the aryl core formed by thienyl groups in one switch is replaced by thiazolyl groups in another.

## Introduction

Aromaticity and antiaromaticity are two fundamental physical organic chemistry concepts that underlie several molecular properties of polycyclic conjugated systems.^1−6^ Hence, a widely used approach to design such molecules with novel functions is to tune their electronic structure on the aromatic–nonaromatic–antiaromatic scale.^7,8^ Apart from structure and reactivity, properties that are influenced by (anti)aromaticity such as molecular stacking or conductance have recently been the subject of major interest. For example, aromatic systems assemble in a manner to minimize π-orbital repulsion,^9,10^ while antiaromatic rings are predicted to prefer face-to-face stacking due to the emergence of transannular delocalization.^11−15^ Furthermore, the increment in conductivity within aromatic, nonaromatic, and antiaromatic molecular wires has been rationalized based on their decreasing resistance to redistribute their π-electron systems upon charge transport.^16−22^ As of now, the best approach to probe the effects of (anti)aromaticity on molecular properties is to design and synthesize new compounds with different conjugation patterns,^23−35^ among which reversible control of (anti)aromaticity has rarely been reported.^20,36,37^

To reversibly control the (anti)aromatic character of individual rings in polycyclic conjugated systems, photochemical mechanisms for rearranging their π-systems are particularly desirable, as electronic excitation circumvents the need to overcome thermal barriers inducible by losses in aromaticity. Photochromic switching that provides opportunities to create dynamic molecular systems can be considered such a mechanism.^38^ Among the established molecular photoswitches, diarylethenes^39^ are of particular interest, as these molecules significantly alter their electronic structure upon photoinduced electrocyclization, which could potentially be exploited in (anti)aromaticity control. A further advantage of this class of molecules is that they can switch also in the solid state, which makes them suitable for thin-film- or single-crystal-based applications.^40^

To construct functional diarylethene switches that are interfaced with benzenoid conjugated systems, one common approach in the literature has been to lower the aromaticity of the ethylene bridge by embedding it in a heteroaromatic unit or a six-membered benzenoid ring with strongly electron-withdrawing substituents.^41−46^ The assumption underlying this approach is that the low aromaticity of the bridge that facilitates thermal electrocyclization (by reducing the associated barriers) is preserved also in the photoactive excited state of the switch. Notably, our groups have recently shown that electronically unperturbed diarylethene switches with an aromatic benzene bridge connecting two thienyl units undergo photoinduced electrocyclization^47^ driven by excited-state antiaromaticity,^48−50^ in accordance with Baird’s rules^51^ (which are the reverse of Hückel’s rules for ground-state aromaticity).

**Scheme 1:**
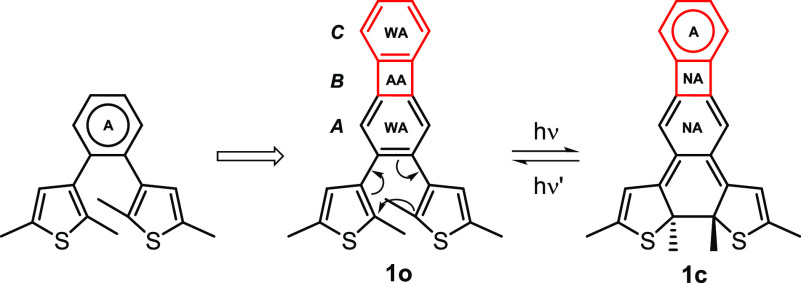
Design of a Dithienylbiphenylene Molecule and the Expected Changes in (Anti)aromaticity of Its ***A***, ***B***, and ***C*** Rings Upon Photocyclization of the Open Isomer (**1o)** into the Closed Isomer (**1c)**^a^ A: aromatic; WA: weakly aromatic; AA: antiaromatic; NA: nonaromatic.

The goal of this work is to elucidate whether the local ground-state (anti)aromaticity of all monocyclic units in a polycyclic bridge fused to a diarylethene framework can be modulated through the ensuing photoswitching. In this regard, bridges that contain both aromatic and antiaromatic subunits are an interesting prospect. To this end, we envisioned a system where the aromatic benzene bridge of dithienylbenzene switches is extended with an antiaromatic benzocyclobutadiene unit, resulting in a dithienylbiphenylene molecule. The open and closed isomers of this molecule, hereafter referred to as **1o** (or **1**, for simplicity) and **1c**, respectively, are shown in Scheme 1.

In the biphenylene bridge of **1**, the two conjugated six-membered rings ***A*** and ***C*** are only weakly aromatic, as a means to minimize the antiaromatic contribution of the central cyclobutadiene ring ***B***.^52^ If **1** is able to undergo photocyclization, the ensuing electronic rearrangement should change the (anti)aromatic character of all three rings ***A***, ***B***, and ***C***, as illustrated in Scheme 1. Additionally, the initially aromatic thienyl moieties would become nonaromatic upon ring closing, which is a characteristic change of dithienylethene photoswitches.^53^ However, besides asserting that photocyclization does indeed promote all of these changes in (anti)aromaticity, it is also desirable to verify that the changes can be reverted back by photocycloreversion of **1c** into **1o**, and that reversible photoswitching between **1o** and **1c** can be achieved for multiple cycles under ambient conditions. In this work, combining organic synthesis, spectroscopic characterization, and quantum chemical calculations, we report on how diarylethene switches can be made to realize all of these goals.

Finally, as for facilitating applications of diarylethene switches, the incorporation of a polycyclic bridge where all monocyclic units change the (anti)aromatic character between the open and closed isomers has a particular potential key advantage. Specifically, such a design might afford high-free-energy barriers for both thermal electrocyclization and thermal cycloreversion if both reactions are impeded by a loss of aromaticity in at least one of the monocyclic units. This characteristic would be appealing for the possibility to use diarylethenes as molecular solar thermal energy (MOST) storage systems^54−57^ that first absorb solar energy through the parent, open form, and then store it as chemical energy in the photoisomerized, closed form.^47^ In particular, a high barrier for the thermal cycloreversion is advantageous in allowing the absorbed solar energy to be stored for a long time, which is one of the main desirable features of MOST systems,^58^ and a high barrier for the thermal electrocyclization is naturally required for the overall efficiency of this technique.

## Results and Discussion

In the following, we first present the synthesis of **1** and characterize its photoswitching by means of UV–vis and proton nuclear magnetic resonance (^1^H NMR) spectroscopy methods. Next, we describe a computational assessment of how the photoswitching changes the local (anti)aromatic character of the individual rings (***A***, ***B***, and ***C***) of the biphenylene moiety of **1**. Noting that the photoswitching is hampered by low fatigue resistance due to the occurrence of a competing, irreversible side-reaction, we then explore different strategies to circumvent this problem. Among these, replacing the thienyl groups of **1** by thiazolyl groups is found to be of particular value.

### Synthesis and Spectroscopic Characterization of the Photoswitching of Compound 1

**Scheme 2:**
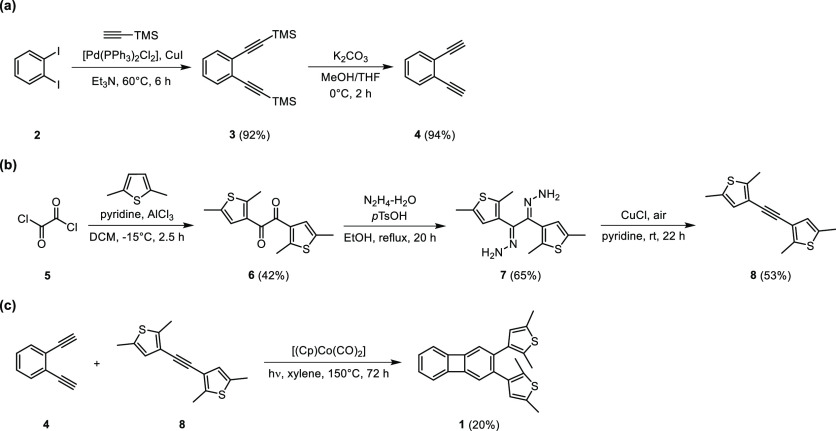
Synthesis of Compound **1**. (a) Synthesis of the Dialkyne Precursor **4**, (b) Synthesis of the Monoalkyne Precursor **8**, and (c) Synthesis of Compound **1** from Alkynes **4** and **8**

The synthesis of compound **1** was achieved by a [2 + 2 + 2] cycloaddition of dialkyne **4** and monoalkyne **8** (Scheme 2). Dialkyne **4** was prepared according to a literature procedure^59^ in two steps (Scheme 2a). 1,2-Diodobenzene was reacted with TMS-acetylene under Sonogashira-coupling conditions, which provided compound **3** in excellent yield. The TMS-protecting groups were removed in a quantitative reaction to obtain the desired dialkyne **4**. Monoalkyne **8**, in turn, was prepared according to a literature procedure^60^ in three steps (Scheme 2b). First, 2,5-dimethylthiophene was reacted with oxalyl-chloride (**5**) in a double Friedel-Crafts reaction leading to diketone **6** in moderate yield (42%). Compound **6** was then condensed with hydrazine hydrate to afford dihydrazone **7**, followed by a Cu(I) mediated aerobic oxidation/elimination sequence to obtain the desired dithienylacetylene derivative **8** in reasonable yield (53%). Finally, compound **1** was synthesized in modest yield by a CpCo(I)-catalyzed [2 + 2 + 2] cycloaddition under light irradiation (white LED) according to a modified literature procedure^61^ (Scheme 2c). The product was characterized by ^1^H and ^13^C NMR spectroscopy. Thereby, ^1^H NMR chemical shifts of protons directly attached to the biphenylene motif (6.61, 6.67, 6.77 ppm, CDCl_3_) suggest that the product has a modest Hückel-aromatic character. The parallel and antiparallel conformers of the dithienylbiphenylene were not distinguishable by NMR spectroscopy at 30 °C, indicating free rotation around the carbon–carbon bonds that connect the biphenylene to the thiophene moieties.

**Figure 1:**
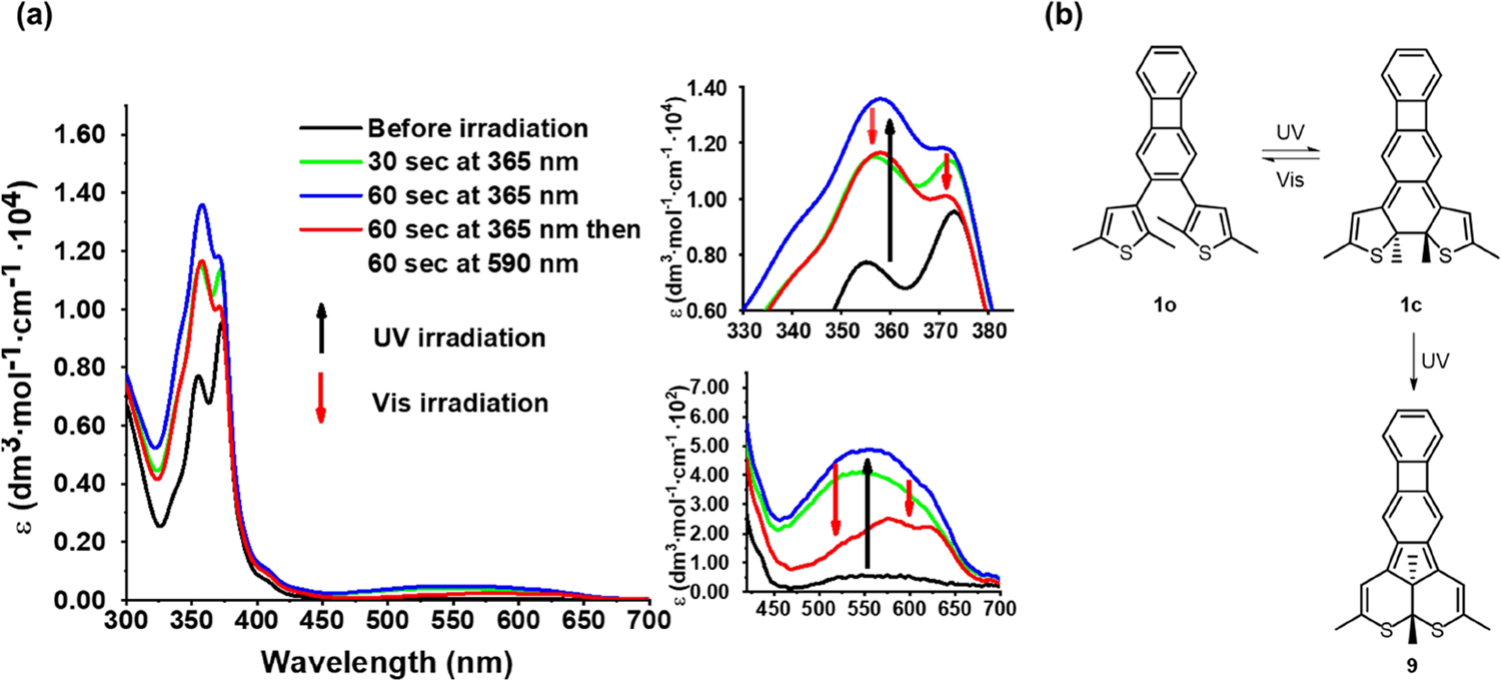
(a) Irradiation experiments of **1o** with UV and visible light in toluene under a nitrogen atmosphere followed by UV–vis spectroscopy (*c* = 4.02 × 10^–5^ M, rt). To better elucidate the scale of the changes at the different wavelengths, ε is *the apparent molar extinction coefficient*, derived from the sample absorbance divided by the concentration of the starting material before irradiation. (b) Formation of side-product **9** from **1c** upon UV irradiation.

The UV–vis absorption spectrum of **1o** in toluene displays a sharp band at 260 nm and two less intense bands at 353 and 372 nm (Figure 1a). Recording the spectrum in different aprotic solvents showed little effect on the bands (Figure S1 in the Supporting Information (SI)). Upon irradiation of a sample of **1o** in toluene with UV light (365 nm), the intensity of the bands at 353 and 372 nm increased, while a new band appeared at 550 nm (Figure 1a). Similar changes were observed upon irradiation in CH_3_CN (Figure S2 in the SI). A closer look at the spectra obtained after 30 and 60 s of irradiation revealed that the two bands at 353 and 372 nm increased independently. Furthermore, when the resulting solution was irradiated with visible light (590 nm), the intensity of the peaks at 353, 372, and 550 nm decreased, but the initial spectrum could not be regenerated completely.

**Figure 2:**
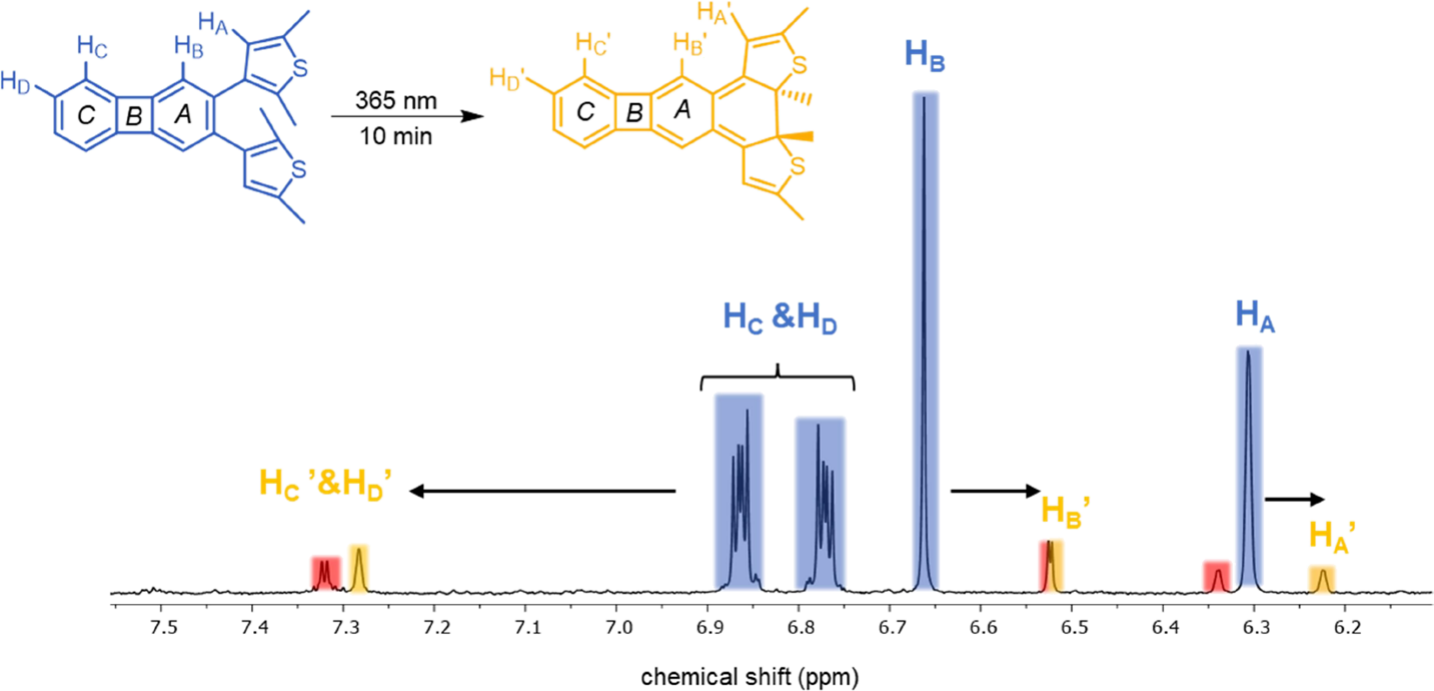
Aromatic region of the ^1^H NMR (500 MHz) spectrum of an irradiated sample of **1o** (CD_3_CN, N_2_, 10 min at 365 nm). Changes in (anti)aromaticity of the individual rings can be estimated from the chemical shifts of the protons directly attached to **1o** and **1c**. Proton resonances of side-product **9** are highlighted in red.

These observations prompted us to speculate on the existence of two distinct photochemical processes driven by UV irradiation. In particular, the occurrence of a side-reaction that transforms the closed isomer **1c** into an annulated bis(dihydro-thiopyran) side-product (**9** in Figure 1b) could be confirmed by ^1^H NMR spectroscopy (Figure 2). Furthermore, this species, which is a well-known source of fatigue within dithienylethene-type molecular switches,^62−65^ could be isolated and characterized (Section S3 in the SI). Although the side-reaction clearly hampers the reversible photoswitching of **1**, the process is not fully inhibited (Figures 1a and S7 and S8 in the SI).

To monitor the changes in aromaticity resulting from the photocyclization of compound **1o**, a detailed ^1^H NMR spectrum of the aromatic region of **1** was recorded in a CD_3_CN solvent (Figures 2 and S8 in the SI). Thereby, resonances of protons attached to ring ***A*** and the thiophenes (H_A_ and H_B_) shifted upfield, whereas signals from protons attached to ring ***C*** (H_C_ and H_D_) shifted significantly downfield (Figure 2). This suggests that the photocyclization disrupts the π-electron system of the biphenylene, making ring ***A*** nonaromatic, while the outer benzene ring ***C*** attains a more pronounced aromatic character. In the next section, the extent to which this picture of a gain (loss) in the aromaticity of ring ***C*** (***A***) is consistent with the results from quantum chemical calculations will be analyzed.

### Quantum Chemical Calculations

To further assess the effect of photocyclization of **1o** on the local (anti)aromatic character of rings ***A***, ***B***, and ***C***, nucleus-independent chemical shift (NICS) indices^66,67^ were calculated with density functional theory (DFT) methods, as fully detailed in Section S7 of the SI. Briefly, providing a magnetic measure of (anti)aromaticity by probing ring currents resulting from circulating π-electrons, these indices were calculated by using gauge-including atomic orbitals and an NICS-scan procedure.^68,69^ Thereby, so-called NICS_zz_ values were obtained for each ring of the biphenylene at distances 1.50/1.60/1.70/1.80/1.90/2.00 Å above the geometric center of the ring in question. The NICS-scan procedure was adopted to alleviate the arbitrariness associated with single-point NICS_zz_ calculations and to reduce σ-electron contaminations of the induced magnetic field,^68,69^ as further described in Section S7 of the SI. For ease of analysis, the discussion of these results below focuses on the NICS_zz_ values obtained at 1.70 Å, which is the distance that Gershoni-Poranne and Stanger^69^ recommend for calculations of this kind. All calculations were carried out with the B3LYP hybrid density functional in combination with the cc-pVTZ basis set and the SMD continuum solvation model^70^ to describe a toluene solvent (this solvent was used in the UV–vis irradiation experiments summarized in Figure 1).

**Figure 3:**
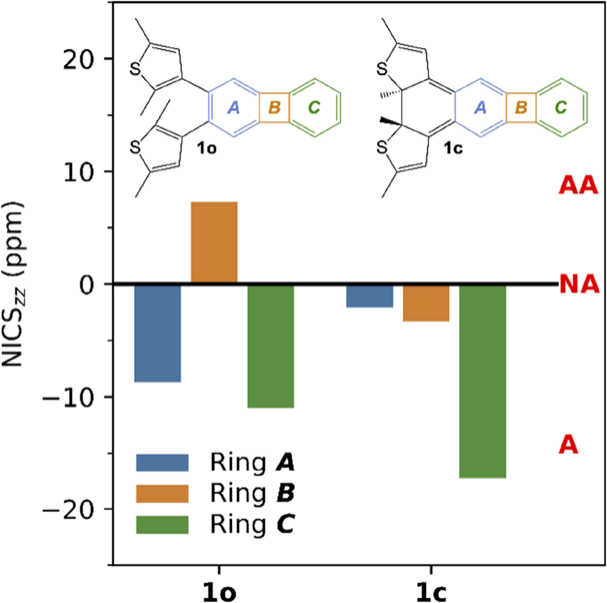
NICS_zz_ values for rings ***A***–***C*** of **1o** and **1c** calculated 1.70 Å above the geometric ring centers. (A: aromatic; WA: weakly aromatic; AA: antiaromatic; NA: nonaromatic).

The NICS_zz_ values calculated for **1o** and **1c** at 1.70 Å are summarized in Figure 3, whereas the full set of NICS_zz_ values is provided in Table S3 of the SI. Noting that negative/positive values indicate aromaticity/antiaromaticity and values close to zero indicate nonaromaticity,^66^ it can first be seen that ring ***A*** upon photocyclization loses the weak aromaticity that it shows in **1o** (NICS_zz_ = −8.7 ppm) by becoming nonaromatic in **1c** (NICS_zz_ = −2.1 ppm). Ring ***B***, in turn, also becomes nonaromatic in **1c**, but rather by relief of antiaromaticity, as can be inferred from the change in the NICS_zz_ value from 7.3 ppm in **1o** to −3.3 ppm in **1c**. At the same time, ring ***C*** experiences a gain in aromaticity upon photocyclization, going from being quite weakly aromatic in **1o** (NICS_zz_ = −11.0 ppm) to being more distinctly aromatic in **1c** (NICS_zz_ = −17.3 ppm). Overall, these results suggest that the local (anti)aromatic character of each ring of the biphenylene can indeed be controlled through the photocyclization of **1o**.

To further corroborate this conclusion and complement the magnetic NICS indices, geometric harmonic oscillator model of aromaticity (HOMA)^71,72^ and electronic Shannon aromaticity (SA)^73,74^ indices were also calculated, as detailed in Section S7 of the SI. The corresponding results are given in Table S4 of the SI. The HOMA index probes the deviation of the carbon–carbon bond lengths of the conjugated ring system in question from an ideal value associated with the fully aromatic benzene molecule. In particular, this index is designed to approach 1, 0, and −1 for an aromatic, nonaromatic, and antiaromatic system, respectively.^71,72^ The SA index, in turn, probes the variation in electron density at bond critical points of the conjugated ring, with aromatic/antiaromatic systems typically showing SA values below/above 0.003/0.005.^73,74^ From Table S4, the changes in HOMA and SA values upon photocyclization are consistent with those in NICS_zz_ values described above. Accordingly, ring ***A*** loses aromaticity (the HOMA/SA value goes from 0.76/0.0013 in **1o** to −0.32/0.0065 in **1c**), ring ***B*** is relieved of antiaromaticity (the HOMA/SA value goes from −0.85/0.0062 in **1o** to −0.60/0.0024 in **1c**), and ring ***C*** gains some aromaticity (the HOMA/SA value goes from 0.87/0.0006 in **1o** to 0.93/0.0002 in **1c**).

DFT calculations were also performed to estimate the relative free energies of **1o** and **1c**, as well as the free-energy barriers for their thermal electrocyclization and cycloreversion reactions. These results, which are summarized in Table S5 of the SI, indicate that systems of this kind are an interesting prospect for the development of MOST applications. In particular, as mentioned as a possibility in Introduction, both reactions have a high barrier, amounting to ∼206 (electrocyclization) and ∼105 kJ mol^–1^ (cycloreversion). Furthermore, the fact that **1c** lies ∼101 kJ mol^–1^ higher in energy than **1o** suggests that a reasonably high energy-storage density can be achieved by this system upon photocyclization. In fact, with a molecular weight of ∼373 g mol^–1^, the value of 101/373 ≈ 0.27 MJ kg^–1^ attained by **1** comes quite close to the often-quoted target value of 0.30 MJ kg^–1^ for MOST applications.^75^

### Strategies To Improve the Reversible Photoswitching through Improved Fatigue Resistance

Overall, the UV–vis and NMR spectroscopic data reveal that the photocyclization of **1o** into **1c** occurs readily. Furthermore, the experimental and computational results show that this reaction modulates the local (anti)aromaticity of all monocyclic units of the biphenylene moiety. However, exploiting such modulation for the realization of reversible tuning of (anti)aromaticity and associated material properties (such as conductance) in future molecular electronic devices would require that the fatigue resistance of the system is improved. Indeed, ideally one would like to achieve reversible photoswitching between **1o** and **1c** for multiple cycles under ambient conditions. In this regard, photoswitching in the solid state is an appealing prospect, because this has been shown to suppress the formation of the annulated side-product^62,63^ and is desirable for thin-film- and single-crystal-based applications.^41^ Unfortunately, however, solid-state irradiation of a film of **1o** with 365 nm light produced absorption bands at ∼600 nm characteristic of the side-product (Figure S5 in the SI). Moreover, when the sample subsequently was irradiated with visible light, only minor reversibility was observed, suggesting that most of **1c** rearranged to side-product **9** during the initial UV irradiation.

**Figure 4:**
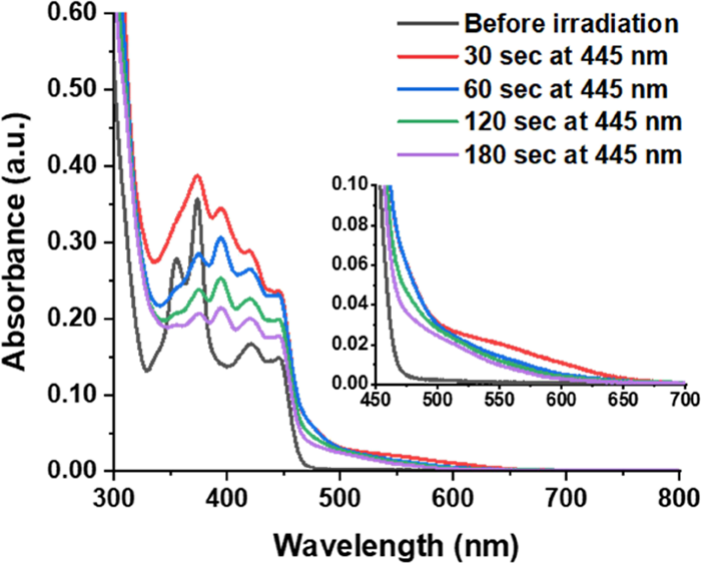
Irradiation of **1o** (*c* = approx. 3 × 10^–5^ M, rt) with 445 nm light in toluene in the presence of diacetyl (*c* = approx. 3 × 10^–3^ M) as a triplet sensitizer. The solution was thoroughly degassed by the freeze-pump-thaw technique.

Turning to other possible strategies to suppress the formation of **9**, we then performed triplet-sensitization experiments.^63,76,77^ Irradiation of **1o** with 445 nm light in the presence of diacetyl (∼100 equiv) produced a red-shifted absorption between 400 and 450 nm, as well as a weak-intensity band at 550 nm (Figure 4). The visible absorption is consistent with the initial absorption changes when **1o** was irradiated with 365 nm light in the absence of diacetyl (Figure 1a), indicating that this band (at 550 nm) corresponds to **1c**. Prolonged irradiation (>30 s) resulted in intensity loss of all bands, which suggests that all species undergo degradation in the presence of the sensitizer. A similar degradation of a biphenylene derivative in the presence of a triplet sensitizer has been observed by Ottosson and co-workers.^78^ Because they used naphthalene as the sensitizer, we believe that the underlying degradation process might be independent of the nature of the sensitizer molecule. The initial formation of **1c** in the sensitization experiments was also confirmed by ^1^H NMR spectroscopy (Figure S10 in the SI).

**Scheme 3:**
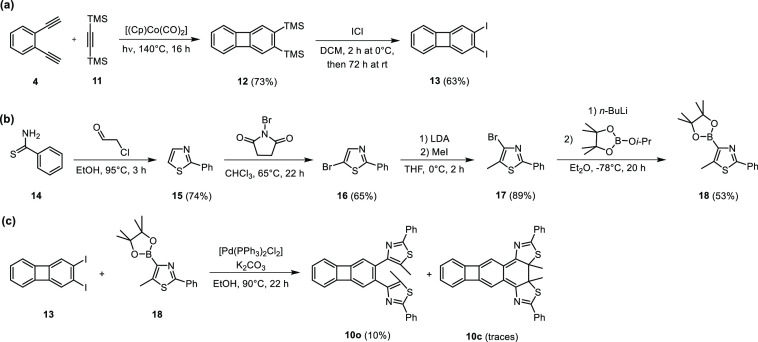
Synthesis of Compound **10**; (a) Synthesis of the 2,3-Diiodobiphenylene Precursor **13**, (b) Synthesis of the Thiazolylboronic Acid Derivative **18**, and (c) Synthesis of Compound **10** from Compounds **13** and **18**

As the solid-state irradiation and triplet-sensitization experiments achieved only limited success in improving the fatigue resistance of **1**, we then turned to synthetic modifications of its aryl core. In particular, retaining the biphenylene bridge, we decided to replace the thienyl groups of **1** with thiazolyl groups. To this end, and because of the straightforward synthetic access to its components^79,80^ and the previously reported reliable photochemistry of dithiazolylarenes,^81,82^ we synthesized compound **10**, a 2,3-dithiazoylbiphenylene (Scheme 3), via a Suzuki–Miyaura cross-coupling between 2,3-diiodobiphenylene (**13**) and thiazolylboronic acid derivative **18**. Both **13** and **18** were prepared according to literature procedures.^68,80^ Briefly, **13** was synthesized via a CpCo(I)-catalyzed [2 + 2 + 2] trimerization between diacetylene **4** and bis(trimethylsilyl)acetylene **11**, followed by an iododesilylation^68^ (Scheme 3a). **18**, in turn, was prepared via the condensation of thiobenzamide **14** and 2-chloroacetaldehyde followed by the bromination of the resulting heterocycle **15** using NBS. The brominated compound **16** was reacted with LDA and iodomethane to yield **17** in excellent yield. Compound **17** was subjected to *n*-butyllithium, and the resulting lithiated heterocycle was quenched with isopropyl-pinacol-borate to obtain **18** in moderate yield^80^ (Scheme 3b).

**Figure 5:**
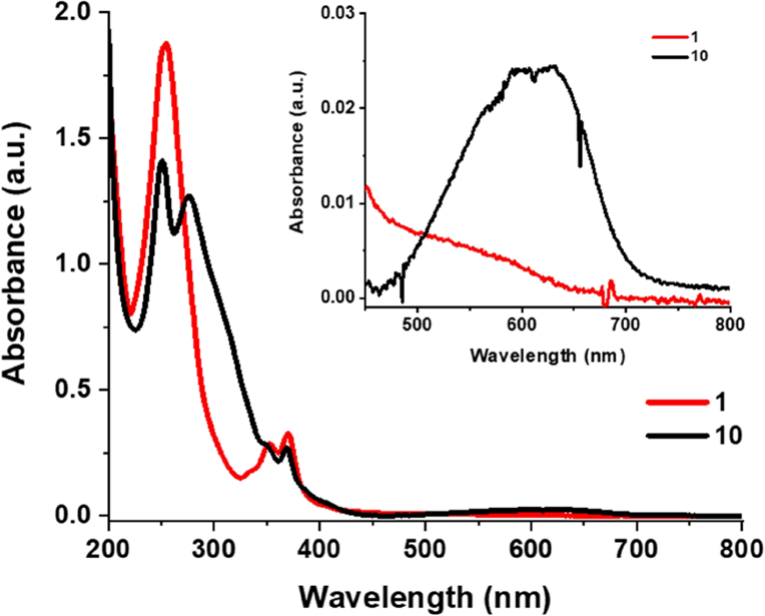
UV–vis spectra of **1** and **10** in CH_3_CN (*c* = approx. 3 × 10^–5^ M, rt).

The UV–vis spectrum of **10** in CH_3_CN shows two distinct absorption maxima (at 251 and 276 nm) below 300 nm compared to the single band (at 254 nm) that **1** displays in this region (Figure 5). Furthermore, in the visible region of the UV–vis spectrum of **10**, a band centered around 600 nm could be observed that is characteristic of the closed form of the molecule, which suggests that **10** is sensitive to ambient light. Indeed, this band disappeared when the solution was irradiated with visible (620 nm) light (Figures 5 and S18 in the SI). Moreover, the formation of a trace amount of **10c** from **10o** under ambient light is confirmed by the observed color change of unprotected samples (Figure S19 in the SI). Similar to the situation for **1**, the UV–vis spectrum of **10** showed only moderate solvent dependence (Figure S16 in the SI).

**Figure 6:**
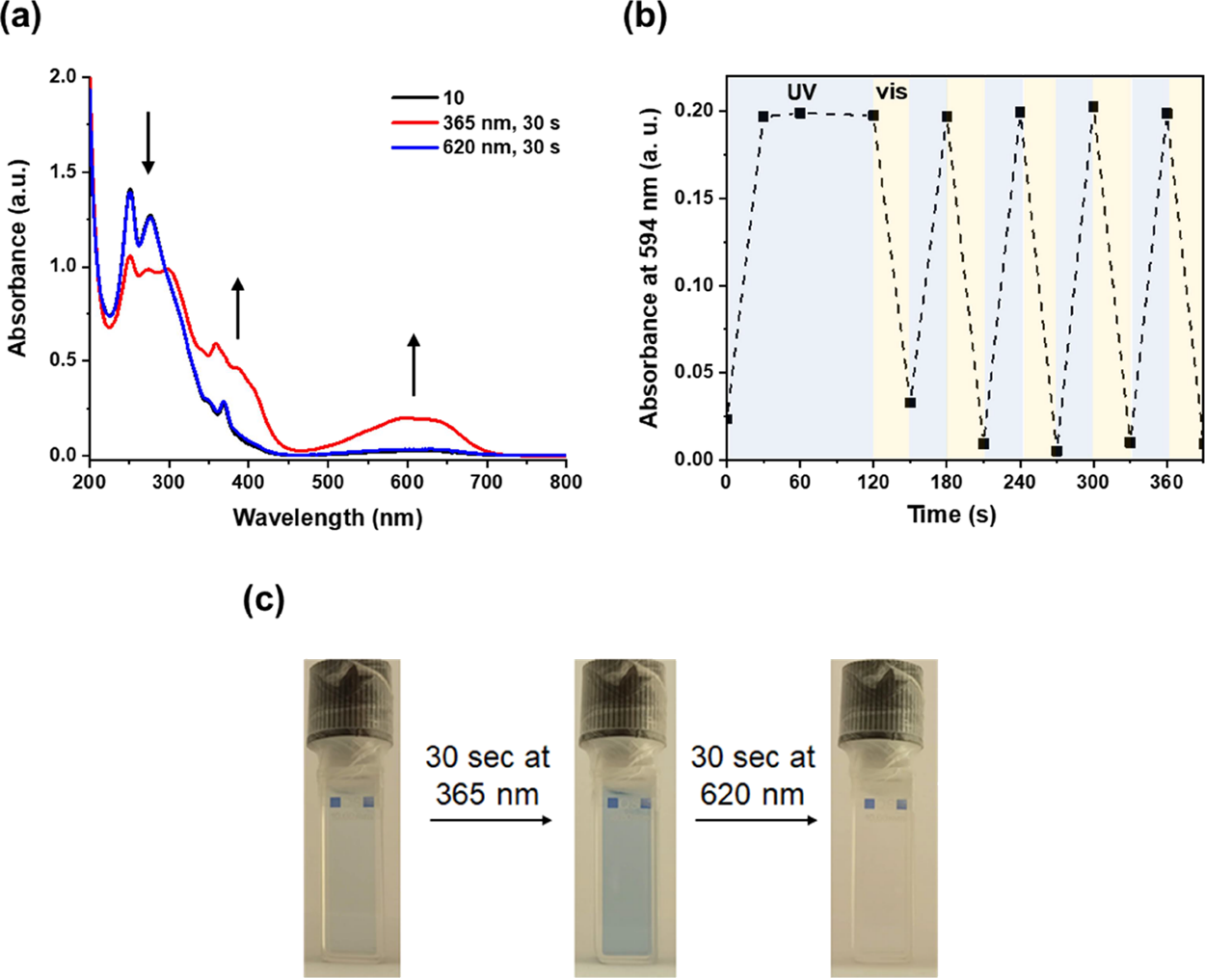
(a) Irradiation of **10** with UV and visible light followed by UV–vis spectroscopy. (b) UV and visible light irradiation cycles of **10** in CH_3_CN (*c* = approx. 3 × 10^–5^ M, rt). (c) Solution of **10** in CH_3_CN before irradiation, after UV irradiation, and after visible light irradiation.

Irradiation experiments with **10o** to generate **10c** were performed in CH_3_CN using 356 nm light (Figure 6a). Upon UV irradiation, the appearance of a blue color (Figure 6c) along with new absorption bands around 400 and 600 nm is indicative of the formation of **10c**. Based on ^1^H NMR spectroscopy, a composition of 1:1.6 (**10o**/**10c**) at the photostationary state was reached in dichloromethane after 95 min of irradiation, while an improved composition of 1:4.8 was reached in C_6_D_6_ after 160 min of irradiation (Figures S20 and S22 in the SI). Satisfyingly, the original spectrum could be regenerated by irradiating the solution with visible light (620 nm). This latter observation confirms that replacing the thiophene units in **1** with thiazole moieties in **10** is sufficient to efficiently suppress the formation of the annulated side-product of type **9**. Furthermore, repeated UV and visible irradiation cycles did not lead to observable degradation or side-product formation (Figure 6b). Reversible switching of **10** was also demonstrated in dichloromethane and methanol solutions (Figure S18 in the SI). Moreover, the photostability of **10** is also considerably higher than that of **1**. In particular, while the irradiation experiments of **1** had to be performed under oxygen-free conditions to avoid degradation, in the case of **10** this was not necessary for reversible switching (Figure S18 in the SI). It is noted, however, that prolonged exposure to UV light in CH_3_CN led to the degradation of **10**.

In addition to probing its photostability, we also assessed the thermal stability of **10c** (Section S6 in the SI). Measurements by UV–vis spectroscopy in toluene revealed that, at 110 °C, most of **10c** isomerizes back to **10o** within 1 h. Furthermore, we conducted kinetics measurements of the thermal cycloreversion in C_6_D_6_ at 70 and 90 °C using ^1^H NMR spectroscopy. These measurements revealed that, at 70 °C, the half-life for the process is about 54 h, while it is about 5.7 h at 90 °C. Based on these results, the activation energy for the cycloreversion was estimated to be 117 kJ mol^–1^, which is quite close to the calculated value of 104.8 kJ mol^–1^ (Table S5). Notably, no thermal decomposition or side-product formation was observed in these experiments.

Finally, regarding the structural changes made to **1** that resulted in improved fatigue resistance in **10**, it should be noted that these do not affect the modulation of the local (anti)aromaticity of each ring of the biphenylene achieved by **1** upon photoswitching. In particular, NICS_zz_, HOMA, and SA values calculated for both the open and closed forms of **10** predict changes in local (anti)aromaticity that are very similar to those that were predicted for **1** by analogous calculations. The corresponding results are summarized in Figure S28 and Tables S3 and S4 of the SI.

Overall, then, we have shown that reversible photoswitching of diarylbiphenylenes is readily achievable under ambient conditions and that this process can be exploited to reversibly tune the local (anti)aromaticity of all three monocyclic units of the biphenylene moiety. For future research, and clearly beyond the scope of the present work, it will be of interest to also investigate aromaticity changes in the excited state *during* the photoswitching and gain a mechanistic understanding of why **10** exhibits better fatigue resistance than **1**. From a computational point of view, exploring these problems poses a considerable but worthwhile challenge in photochemical modeling.

## Conclusions

In summary, we have demonstrated efficient and reversible photoswitching by diarylethenes featuring a biphenylene group as the ethene bridge between the two aryl units. Starting with a compound (**1**) for which the aryl core is constituted by thienyl groups, the photoswitching is assessed experimentally using UV–vis and NMR spectroscopy methods. Probing changes in the local (anti)aromaticity of the individual rings of the biphenylene in terms of experimental ^1^H NMR chemical shifts and calculated NICS, HOMA, and SA aromaticity indices, it is found that the character of each ring is altered through the photoswitching. Although **1** is found to undergo reversible photoswitching, the process is hampered by the irreversible formation of an annulated bis(dihydro-thiopyran) side-product (**9**).

Next, to bypass this problem and improve the fatigue resistance of the switch, which is a prerequisite for its potential use in applications, the thienyl groups in the aryl core of **1** were replaced by thiazolyl groups. This structural change resulted in a switch (**10**) capable of reversible operation under ambient conditions without considerable degradation even after multiple switching cycles. Overall, we conclude that the photoswitching can be used to control the local (anti)aromatic character of each ring of the biphenylene in a reversible fashion. In future research, we plan to investigate whether other diarylethene switches can be made to control the local (anti)aromatic character of other polycyclic conjugated systems in a similar fashion.

## Experimental Section

### General Information

Commercial reagents, solvents, and catalysts (Aldrich, Fluorochem, VWR) were purchased as reagent grade and used without further purification. Solvents for extraction or column chromatography were of technical quality. For spectroscopy and sample treatment, opti-grade quality solvents were used. Organic solutions were concentrated by rotary evaporation at 25–40 °C. Thin-layer chromatography (TLC) was carried out on SiO_2_–layered aluminum plates (60778-25EA, Fluka). Column chromatography was performed using SiO_2_–60 (230–400 mesh ASTM, 0.040–0.063 mm from Merck) at 25 °C or using a Teledyne Isco CombiFlash Rf + automated flash chromatographer with silica gel (25–40 μm, Redisep Gold). Room temperature refers to 25–20 °C depending on the time of day.

NMR spectra were acquired on a Varian 500 NMR spectrometer, running at 500 and 126 MHz for ^1^H and ^13^C. The residual solvent peaks were used as the internal reference. Chemical shifts (δ) are reported in ppm. The following abbreviations are used to indicate the multiplicity in ^1^H NMR spectra: s, singlet; d, doublet; t, triplet; q, quartet; p, pentett; m, multiplet. ^13^C NMR spectra were acquired on a broad band decoupled mode.

UV–vis spectrophotometry was executed on a Jasco V-750 or a PerkinElmer Lambda 465 spectrophotometer. Hellma Analytics High Precision quartz cuvettes were used with an optical path length of 1.0 cm. Irradiation of samples was carried out with 10 W COB LED lights with nominal emission maxima at 365, 450, 590, and 620 nm (for measured emission spectra, see Figure S56 in the SI).

High-resolution measurements were performed on a Sciex TripleTOF 5600+ high-resolution tandem mass spectrometer equipped with a DuoSpray ion source. Electrospray ionization was applied in positive-ion detection mode. Samples were dissolved in acetonitrile and flow injected into acetonitrile/water 1:1 flow, or otherwise noted. The flow rate was 0.2 mL/min. The resolution of the mass spectrometer was 35,000.

#### Synthesis of 1,2-Bis((trimethylsilyl)ethynyl)benzene (**3**)

A round-bottom flask was charged with Pd(PPh_3_)_2_Cl_2_ (298 mg, 0.424 mmol) and CuI (40.4 mg, 0.212 mmol) and purged with N_2_. 1,2-Diiodobenzene (14.0 g 42.44 mmol) and ethynyltrimethylsilane (18.2 mL, 131.6 mmol) dissolved in triethylamine (100 mL) were added, and the mixture was heated in an oil bath and refluxed at 60 °C for 6 h.^83^ After completion, the reaction mixture was filtered through a Celite pad. The solvent was evaporated in vacuo, and the crude product was further purified by column chromatography (SiO_2_, *n*-hexane) to yield the product (10.6 g, 92%) as a yellow oil, which solidified in the freezer. ^1^H NMR (500 MHz, CDCl_3_) δ = 7.45 (dd, *J* = 5.8, 3.4 Hz, 2H), 7.23 (dd, *J* = 5.8, 3.3 Hz, 2H), 0.27 ppm (s, 18H); ^13^C{^1^H} NMR (126 MHz, CDCl_3_) δ = 132.5 (2), 128.2 (2), 126.0 (2), 103.4 (2), 98.6 (2), 0.2 (6) ppm.

#### Synthesis of 1,2-Diethynylbenzene (**4**)

Compound **3** (10.00 g, 14.8 mmol) and K_2_CO_3_ (25.5 g, 73.9 mmol) were dissolved in a mixture of methanol (200 mL) and THF (200 mL) at 0 °C.^79^ The mixture was stirred at 0 °C in an ice bath for 2 h. After completion, the solvent was evaporated in vacuo, the crude product was re-dissolved in EtOAc (100 mL) and washed with water (3 × 50 mL), and the organic layer was dried over MgSO_4_. Removal of the solvent gave compound **4** (4.38 g, 94%) as a crimson oil. ^1^H NMR (500 MHz, CDCl_3_) δ = 7.52 (dd, *J* = 5.7, 3.4 Hz, 2H), 7.31 (dd, *J* = 5.8, 3.4 Hz, 2H), 3.33 ppm (s, 2H); ^13^C{^1^H} NMR (126 MHz, CDCl_3_) δ = 132.8 (2), 128.6 (2), 125.2 (2), 82.0 (2), 81.3 (2) ppm.

#### Synthesis of 1,2-Bis(2,5-dimethylthiophen-3-yl)ethane-1,2-dione (**6**)

A three-necked flask equipped with a thermometer was charged with AlCl_3_ (6.0 g, 44.9 mmol) and purged with N_2_. CH_2_Cl_2_ (50 mL) was added, and the suspension formed upon stirring was cooled to −15 °C using an NaCl/ice bath.^60^ Pyridine (1.8 mL, 22.3 mmol) dissolved in CH_2_Cl_2_ (10 mL) and 2,5-dimethylthiophene (5.0 g, 44.6 mmol) dissolved in CH_2_Cl_2_ (25 mL) were added followed by the dropwise addition of oxalyl chloride (2.3 mL, 26.8 mmol) dissolved in CH_2_Cl_2_ (25 mL) in ∼90 min at −15 °C. After the addition was complete, the reaction was allowed to warm to 5 °C in 60 min. The mixture was poured on ice cold water (200 mL), and the organic phase was separated. The aqueous layer was extracted with CHCl_3_ (3 × 200 mL) and the combined organic layer was washed with water and saturated Na_2_CO_3_ solution and dried over MgSO_4_. The solvent was evaporated under reduced pressure, and the dark residue was further purified by column chromatography (SiO_2_, *n*-hexane/EtOAc 9:1) to obtain the product as a red oil (2.6 g, 43%). ^1^H NMR (500 MHz, CDCl_3_) δ = 6.91 (s, 2H), 2.72 (s, 6H), 2.38 ppm (s, 6H). ^13^C{^1^H} NMR (126 MHz, CDCl_3_) δ = 189.2 (2), 151.5 (2), 136.2 (2), 131.8 (2), 126.9 (2), 15.9 (2), 14.8 (2) ppm.

#### Synthesis of 1,2-Bis(2,5-dimethylthiophen-3-yl)-1,2-dihydrazonoethane (**7**)

Compound **6** (4.0 g, 14.32 mmol) and *p*-toluenesulfonic acid (130 mg, 0.7 mmol) were dissolved in EtOH (30 mL), and hydrazine hydrate was added (7.0 mL, 144 mmol).^60^ The reaction was heated in an oil bath and refluxed for 20 h. After completion, the reaction mixture was cooled to 0 °C and filtered, and the solid was washed with ice cold EtOH and allowed to dry on air. Compound **7** was obtained as a white/pale yellow solid (2.91 g, 65%). ^1^H NMR (500 MHz, DMSO-d_6_) δ = 6.45 (s, 2H), 6.05 (s, 4H), 2.38 (s, 6H), 2.15 ppm (s, 6H). ^13^C{^1^H} NMR (126 MHz, DMSO-d_6_) δ = 143.1 (2), 135.6 (2), 133.6 (2), 129.8 (2), 126.3 (2), 14.8 (2), 13.8 (2) ppm.

#### Synthesis of 1,2-Bis(2,5-dimethylthiophen-3-yl)ethyne (**8**)

CuCl (653 mg, 6.6 mmol) was dissolved in pyridine (25 mL) and stirred vigorously in an open flask for 30 min.^60^ Compound **7** (1.0 g, 3.26 mmol) was added in four equal portions in 2 h, and then the reaction mixture was stirred for 22 h on air. After completion, 3 M HCl was added, and the mixture was extracted with Et_2_O three times. The combined organic layer was washed with water and brine and dried over MgSO_4_. The crude product was further purified by column chromatography (SiO_2_, *n*-hexane) to obtain compound **8** as a white solid (403 mg, 53%). ^1^H NMR (500 MHz, CDCl_3_) δ = 6.66 (s, 2H), 2.50 (s, 6H), 2.40 ppm (s, 6H); ^13^C{^1^H} NMR (126 MHz, CDCl_3_) δ = 140.6 (2), 135.8 (2), 127.3 (2), 119.8 (2), 86.2 (2), 15.3 (2), 14.5 (2) ppm.

#### Synthesis of 2,3-Bis(2,5-dimethylthiophen-3-yl)biphenylene (**1**)

Compound **4** (100 mg, 0.79 mmol), compound **8** (205 mg, 0.83 mmol), and cyclopentadienylcobalt dicarbonyl (14.3 mg, 10.6 μL, 0.08 mmol) were dissolved in xylene (1.5 mL, mixture of isomers) in a glove box. The solution was transferred to a syringe and added to xylene (5.0 mL, mixture of isomers, under an N_2_ atmosphere) in 8 h at 150 °C (the reaction was heated in an oil bath), under visible light irradiation. After the addition was complete, the reaction mixture was refluxed and irradiated for an additional ∼60 h under an N_2_ atmosphere. After completion, the reaction mixture was quenched with water and diluted with EtOAc. The organic layer was washed with water and brine, dried over MgSO_4_, and filtered, and the solvent was evaporated under reduced pressure. The crude product was further purified by column chromatography (SiO_2_, *n*-hexane) to obtain compound **1** as a pale-yellow oil (60 mg, 20%), which solidified in the freezer. ^1^H NMR (500 MHz, CDCl_3_) δ = 6.77 (dd, *J* = 4.9, 2.8 Hz, 2H), 6.67 (dd, *J* = 4.8, 2.8 Hz, 2H), 6.61 (s, 2H), 6.23 (s, 2H), 2.32 (s, 6H), 2.05 ppm (s, 6H); ^13^C{^1^H} NMR (126 MHz, CDCl3) δ = 150.9 (2), 149.4 (2), 138.0 (2), 135.9 (2), 134.5 (2), 132.5 (2), 128.3 (2), 127.5 (2), 119.9 (2), 117.5 (2), 15.0 (2), 13.8 (2) ppm. HRMS (APCI) *m*/*z*: [M + H]^+^ calcd for C_24_H_21_S_2_^+^: 373.1079; found 373.1081.

#### Synthesis of 2,3-Bis(trimethylsilyl)biphenylene (**12**)

Compound **4** (230 mg, 1.83 mmol) was dissolved in bis(trimethylsilyl)acetylene (3 mL), and the solution was transferred to a glove box.^79^ (Cp)Co(CO)_2_ (25 μL, 0.18 mmol) was added, and the solution was transferred to a syringe, which was sealed by a rubber septum. The sealed syringe was removed from the glove box and transferred to a syringe pump, and the contents were added to bis(trimethylsilyl)acetylene (12.5 mL) in 8 h at 140 °C (the reaction was heated in an oil bath) under an N_2_ atmosphere and visible light irradiation. After the addition was complete, the reaction mixture was heated at 140 °C for an additional 8 h. The reaction was quenched with water and diluted with EtOAc. The organic layer was washed with water and brine and dried over MgSO_4_. The solvent was evaporated, and the crude mixture was purified by column chromatography (SiO_2_, *n*-hexane) to obtain the product as a reddish oil (393 mg, 73%). ^1^H NMR (500 MHz, CDCl_3_) δ = 6.96 (s, 2H), 6.73 (dd, *J* = 4.9, 2.9 Hz, 2H), 6.66 (dd, *J* = 4.9, 2.9 Hz, 1H), 0.33 ppm (s, 18H); ^13^C{^1^H} NMR (126 MHz, CDCl_3_) δ = 152.7 (2), 150.7 (2), 147.9 (2), 128.3 (2), 122.9 (2), 117.8 (2), 2.3 (6) ppm.

#### Synthesis of 2,3-Diiodobiphenylene (**13**)

Compound **12** (300 mg, 1.01 mmol) was dissolved in dichloromethane (15 mL), and ICl (411 mg, 2.53 mmol) was added at 0 °C.^79^ The reaction was allowed to warm to rt. in 2 h, and then the mixture was stirred at rt. for 72 h. After the reaction was complete, most of the solvent was evaporated at reduced pressure, and the residue was dissolved in Et_2_O. The organic layer was washed with Na_2_S_2_O_3_ solution (2×) and brine, and the combined aqueous layer was extracted with Et_2_O. The combined organic layer was dried over MgSO_4_, concentrated, and purified by column chromatography (SiO_2_, *n*-hexane) to obtain the pure product as a brownish yellow solid (256 mg, 63%). ^1^H NMR (500 MHz, CDCl_3_) δ = 7.15 (s, 2H), 6.81 (dd, *J* = 4.9, 2.9 Hz, 2H), 6.69 ppm (dd, *J* = 4.9, 2.9 Hz, 2H); ^13^C{^1^H} NMR (126 MHz, CDCl3) δ = 151.8 (2), 149.9 (2), 129.3 (2), 127.8 (2), 119.0 (2), 106.4 (2) ppm.

#### Synthesis of 2-Phenylthiazole (**15**)

Thiobenzamide (4.0 g, 39.0 mmol, 1.0 eq.) was dissolved in EtOH (20 mL, 1 M), and chloroacetaldehyde (11.0 mL, 87.0 mmol, 3.0 eq., 50 wt % solution in water) was added.^80^ The dark orange reaction mixture was heated in an oil bath at 95 °C for 3 h. After completion, the solvent was removed under reduced pressure, and the residue was diluted with dichloromethane. The organic phase was washed with water, dried over MgSO_4_, and concentrated. The crude product was further purified by column chromatography (SiO_2_, *n*-hexane/EtOAc 12:1) to obtain compound **15** as a pale-yellow oil (3.5 g, 29 mmol, 74%). ^1^H NMR (500 MHz, CDCl_3_) δ = 7.97 (dd, *J* = 8.0, 1.6 Hz, 2H), 7.87 (d, *J* = 3.2 Hz, 1H), 7.48–7.41 (m, 3H), 7.33 ppm (d, *J* = 3.3 Hz, 1H); ^13^C{^1^H} NMR (126 MHz, CDCl_3_) δ = 168.4, 143.7, 133.7, 130.0, 129.0 (2), 126.6 (2), 118.8 ppm.

#### Synthesis of 5-Bromo-2-phenylthiazole (**16**)

Compound **15** (3.5 g, 21.6 mmol, 1.0 eq.) was dissolved in CHCl_3_ (50 mL, 0.4 M) and *N*-bromosuccinimide (4.0 g, 22.7 mmol 1.05 eq.) was added.^80^ The solution was heated in an oil bath at 65 °C for 24 h. After completion, the solution was transferred to a separation funnel, washed with water, dried over MgSO_4_, and concentrated under reduced pressure. The crude product was further purified by column chromatography (SiO_2_, *n*-hexane/EtOAc 12:1) to obtain the pure product as pink crystals (3.4 g, 14.0 mmol, 65%). ^1^H NMR (500 MHz, CDCl_3_) δ = 7.91–7.84 (m, 2H), 7.74 (s, 1H), 7.48–7.41 ppm (m, 3H); ^13^C{^1^H} NMR (126 MHz, CDCl3) δ = 169.6, 144.9, 133.2, 130.4, 129.1 (2), 126.3 (2), 108.5 ppm.

#### Synthesis of 4-Bromo-5-methyl-2-phenylthiazole (**17**)

Diisopropylamine (2.56 mL, 18.1 mmol, 1.50 eq.) was dissolved in THF (50 mL) under an N_2_ atmosphere and *n*-BuLi (7.25 mL, 18.1 mmol, 1.50 eq., 2.5 M solution in hexanes) was added at 0 °C.^80^ The LDA solution was stirred for 15 min and then added dropwise to a solution of compound **16** (2.90 g, 12.1 mmol, 1.0 eq.) in THF (50 mL) under an N_2_ atmosphere at 0 °C. The red colored reaction mixture was stirred for 15 min followed by the addition of MeI (2.26 mL, 36.2 mmol, 3.0 eq.) at 0 °C. The mixture was stirred at room temperature for 90 min. After completion, the reaction mixture was diluted with water and EtOAc. The organic phase was separated, washed with water and brine, and dried over MgSO_4_. The crude product was further purified by column chromatography (SiO_2_, *n*-hexane/EtOAc 12:1) to obtain compound **17** as a white crystalline solid (2.7 g, 10.7 mmol, 89%). ^1^H NMR (500 MHz, CDCl_3_) δ = 7.93–7.79 (m, 2H), 7.48–7.38 (m, 3H), 2.44 ppm (s, 3H); ^13^C{^1^H} NMR (126 MHz, CDCl_3_) δ = 165.5, 132.9, 130.2, 128.9 (2), 128.7, 126.0 (2), 125.4, 13.0 ppm.

#### Synthesis of 5-Methyl-2-phenyl-4-(4,4,5,5-tetramethyl-1,3,2-dioxaborolan-2-yl)thiazole (**18**)

Compound **17** (254 mg, 1.0 mmol, 1.0 eq.) was dissolved in Et_2_O (10 mL, 0.1 M) in a round-bottom flask under an N_2_ atmosphere.^80^ The solution was cooled to −78 °C, and *n*-BuLi (0.44 mL, 1.1 mmol, 1.10 eq., 2.5 M solution in hexanes) was added dropwise in 15 min. The reaction mixture was stirred for 1 h followed by the addition of 2-isopropoxy-4,4,5,5-tetramethyl-1,3,2-dioxaborolane (0.30 mL, 1.5 mmol, 1.5 eq.). The reaction mixture was allowed to warm to rt overnight. Subsequently, water and EtOAc were added and the organic layer was separated. The organic phase was washed with water and brine and concentrated under reduced pressure. The crude product was further purified by column chromatography (SiO_2_, *n*-hexane/EtOAc 2:1) to obtain compound **18** as a yellow oil (132 mg, 0.54 mmol, 53%), which solidified in the freezer. ^1^H NMR (500 MHz, CDCl_3_) δ = 7.94 (dd, *J* = 7.9, 1.8 Hz, 2H), 7.40–7.31 (m, 3H), 2.72 (s, 3H), 1.37 ppm (s, 12H); ^13^C{^1^H} NMR (126 MHz, CDCl_3_) δ = 166.0, 148.3, 133.8, 129.5, 128.6 (2), 127.1 (2), 83.9, 75.0, 24.9 (4), 12.9 ppm.

#### Synthesis of Compounds **10o** and **10c**

A 30 mL scintillation vial was charged with 2,3-diiodobihenylene **13** (50 mg, 0.12 mmol, 1.0 eq.), compound **18** (112 mg, 0.37 mmol, 3 eq.), K_2_CO_3_ (85 mg, 0.62 mmol, 5 eq.), and Pd(PPh_3_)_2_Cl_2_ (4 mg, 5 mol %). The vial was purged with N_2_ thoroughly and EtOH (1 mL) was added with a syringe. The mixture was stirred at 90 °C for 22 h. The progress of the reaction was monitored by TLC analysis (*n*-hexane/EtOAc 12:1). After completion, the solvent was evaporated, and the crude product was purified by column chromatography (SiO_2_, *n*-hexane/EtOAc 12:1) to obtain compound **10** as a blue colored waxy solid (the blue color is caused by the presence of a trace amount of **10c**) (7 mg, 10%). An analytically pure sample of **10o** was obtained by subjecting a sample of compound **10** to visible light irradiation (620 nm). Pure **10o** is a yellow waxy solid. ^1^H NMR (500 MHz, CD_2_Cl_2_) δ = 7.88–7.82 (m, 4H), 7.42–7.38 (m, 6H), 6.94 (s, 2H), 6.88 (dd, *J* = 4.9, 2.9 Hz, 2H), 6.80 (dd, *J* = 4.9, 2.9 Hz, 2H), 2.10 ppm (s, 6H); ^13^C{^1^H} NMR (126 MHz, CD_2_Cl_2_) δ = 163.4 (2), 152.1 (2), 150.7 (2), 150.6 (2), 134.8 (2), 133.9 (2), 129.9 (2), 129.4 (2), 128.7 (2), 128.7 (2), 126.0 (2), 119.5 (2), 117.9 (2), 11.8 (2) ppm. HRMS (APCI) *m*/*z*: [M + H]^+^ calcd for C_32_H_23_N_2_S_2_^+^: 499.1308; found 499.1320.

The ^1^H NMR characterization of compound **10c** is based on the PSS mixture following the irradiation of **10o** (Figure S21 in the SI). ^1^H NMR of **10c** (500 MHz, CD_2_Cl_2_) δ = 7.99 (dd, *J* = 7.6, 1.8 Hz, 4H), 7.60–7.49 (m, 6H), 7.38–7.29 (m, 4H), 7.23 (s, 2H), 2.06 ppm (s, 6H).

## Supporting Information Available

UV–vis spectroscopic characterization of **1** and **10**, ^1^H NMR spectroscopic characterization of the photochemical transformations of **1** and **10**, isolation and characterization of side-product **9**, thermal stability assessment of **10c**, computational details and complementary computational results, NMR spectra of reported compounds, HRMS of **1** and **10**, LED emission spectra, and Cartesian coordinates and electronic energies of **1** and **10** (PDF)
