# Supplementary material for: Photoswitching of Local (Anti)Aromaticity in Biphenylene-Based Diarylethene Molecular Switches
Source: J Org Chem. 2022 Jul 18;87(15):9532–42. doi: 10.1021/acs.joc.2c00504 (PMC9361354; doi:10.1021/acs.joc.2c00504)
Supplement: Supplementary file 1 — jo2c00504_si_001.pdf [file jo2c00504_si_001.pdf]

Supporting Information

Photoswitching of Local (Anti)Aromaticity in Biphenylene-Based Diarylethene Molecular Switches

Péter Pál Kalapos,[a] Péter J. Mayer,[a,b] Tamás Gazdag,[a,c] Attila Demeter,[d] Baswanth Oruganti,*[e] Bo Durbeej,*[f] and Gábor London*[a]

[a] MTA TTK Lendület Functional Organic Materials Research Group, Institute of Organic Chemistry, Research Centre for Natural Sciences, 1117 Budapest, Magyar tudósok krt. 2, Hungary

[b] Institute of Chemistry, University of Szeged, Rerrich tér 1., 6720 Szeged, Hungary

[c] Hevesy György PhD School of Chemistry, Eötvös Loránd University, Pázmány Péter sétány 1/a, Budapest 1117, Hungary

[d] Institute of Materials and Environmental Chemistry, Research Centre for Natural Sciences, 1117 Budapest, Magyar tudósok krt. 2, Hungary

[e] Department of Chemistry and Biomedical Sciences, Faculty of Health and Life Sciences, Linnaeus University, SE-45041 Kalmar, Sweden

[f] Division of Theoretical Chemistry, IFM, Linköping University, SE-58183 Linköping, Sweden

Emails: baswanth.oruganti@gmail.com (B.O.); bodur@ifm.liu.se (B.D.);
london.gabor@ttk.hu (G.L.)

Table of Contents

S1. UV-Vis spectroscopic characterization of **1** S3

S2. ^{1}H NMR spectroscopic characterization of the photochemical transformations of **1** S8

S3. Isolation and characterization of side-product **9** S13

S4. UV-Vis spectroscopic characterization of **10** S16

S5. ^{1}H NMR spectroscopic characterization of the photochemical transformations of **10** ... S18

S6. Thermal stability of **10c** S21

S7. Computational details and complementary computational results S26

S8. NMR spectra of reported compounds S31

S9. HRMS of **1** and **10** S44

S10. LED emission spectra S45

S11. References S46

S12. Cartesian coordinates (in Å) and electronic energies (E, in Ha) of **1** and **10** S48

S1. UV-Vis spectroscopic characterization of 1

UV-Vis spectrophotometry was executed on a Jasco V-750 spectrophotometer. Data were collected from 800 to 200 nm using a 1 nm data interval and a 400 or 1000 nm/s scan speed. Hellma Analytics High Precision quartz cuvettes were used with optical path length of 1.0 cm. Irradiation of samples was carried out with LED lamps with nominal emission maxima at 365, 450 and 590 nm in Hellma Analytics High Precision quartz cuvettes. All irradiation experiments were performed under nitrogen atmosphere, unless otherwise indicated. In the presence of atmospheric oxygen only photodegradation could be observed upon irradiation of **1**.

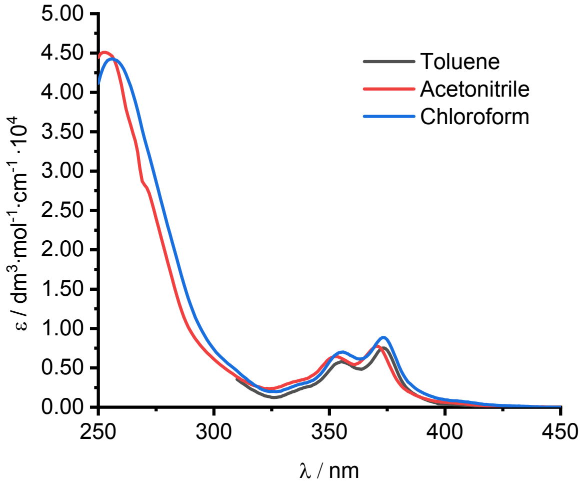

Figure S1. UV-Vis spectra of **1** in different solvents (c = 1.02×10^{-5} M, rt).

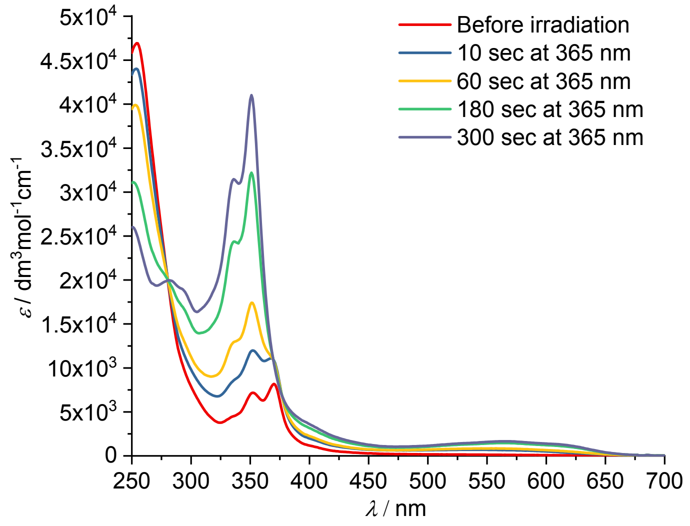

Figure S2. Irradiation of **1** in CH_3CN solution with 365 nm light under nitrogen atmosphere followed by UV-Vis spectroscopy (c = 4.02×10^{-5} M, rt).

Exhaustive irradiation of 1o and thermal stability. Prolonged UV irradiation of a fresh sample of **1o** produced a sharp absorption at 343 nm, while the intensity of the bands at 353, 575 and 625 nm increased (Figure S3). Interestingly, the band at 372 nm that was observed after short irradiation times (Figure 1, see main text) quickly disappeared. After 25 min of UV irradiation no further change was observed. Moreover, the resulting blue solution was stable to visible light irradiation (590 nm) and to high temperatures (110 °C) (Figure S4).

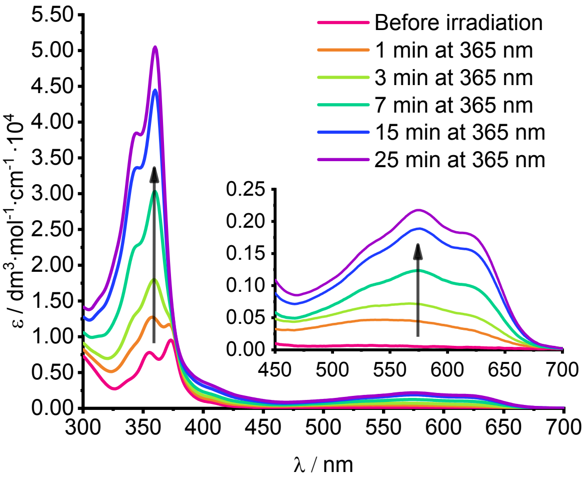

Figure S3. Exhaustive irradiation of **1o** by UV light in toluene solution followed by UV-Vis spectroscopy ($c = 4.02 \times 10^{-5}$ M, rt).

The thermal reversibility of the photoreaction was explored by heating **9** (the side-product, see main text). A sample of **1** that was irradiated exhaustively with 365 nm light (Figure S3) was subjected to high temperatures (70 °C for 10 min and then 110 °C for 40 min, Figure S4).

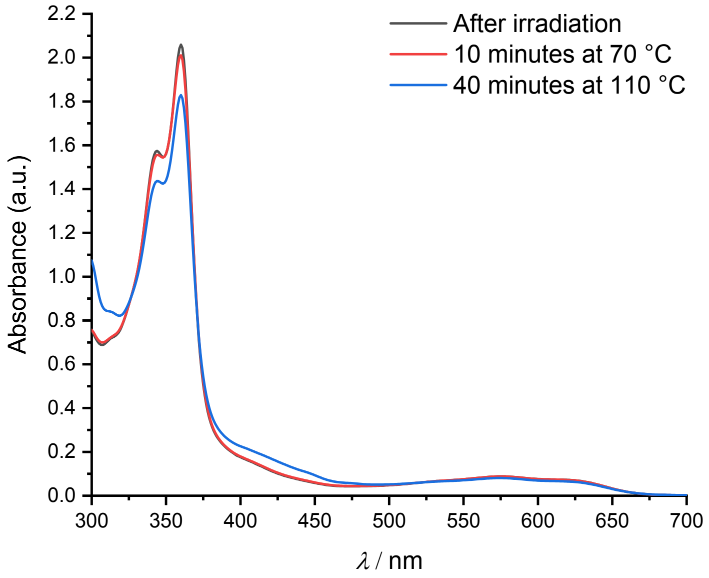

Figure S4. Heating of **9** in toluene solution followed by UV-Vis spectroscopy (c = approx. 3×10^{-5} M, rt).

Upon heating the solution at 70 °C no change was observed. Heating at 110 °C for 40 min resulted in small changes: absorption bands started to appear at 425 and 300 nm (Figure S4). It is possible that, at 110 °C, **9** underwent degradation that gave rise to the new absorptions.

Solid-state irradiation of 1o. As it has been reported that the formation of the annulated side-product may be suppressed in the solid state,[1a,b] we performed solid-state irradiation experiments (Figure S5). A film of **1o** was prepared on the inside of a quartz cuvette by the slow evaporation of solvent from a concentrated sample. The cuvette was thoroughly purged with nitrogen gas. Irradiation with 365 nm light produced characteristic absorption bands of the side-product at ~600 nm. The sample was then irradiated with visible light resulting in a very small amount of reversible change, suggesting that most of the closed-product **1c** rearranged during the initial UV irradiation to side-product **9**.

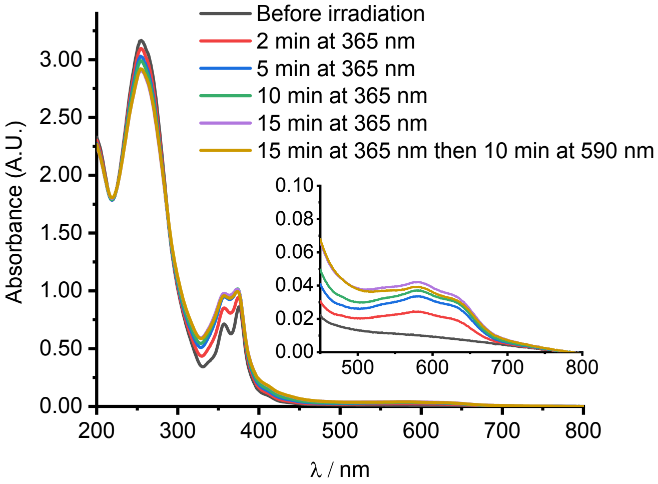

Figure S5. Irradiation of **1o** in the solid state. A film was created by the slow evaporation of a concentrated solution. Irradiation was performed under nitrogen atmosphere.

Triplet-sensitization experiments. 1 was dissolved in toluene and diacetyl (100 eq.) was added and the solution was transferred to a quartz cuvette. The solution was degassed with the freeze-pump-thaw technique (5 cycles) in a special quartz cuvette (Figure S6a). The UV-Vis absorption spectrum was recorded, and the solution was irradiated with 450 nm light.

To establish whether diacetyl is responsible for the change observed in the UV-Vis spectra in Figure 4 of the main text, we performed a control experiment. A solution of **1** in toluene was prepared, thoroughly degassed by the freeze-pump-thaw technique and irradiated at 450 nm *in the absence of diacetyl* (Figure S6b).

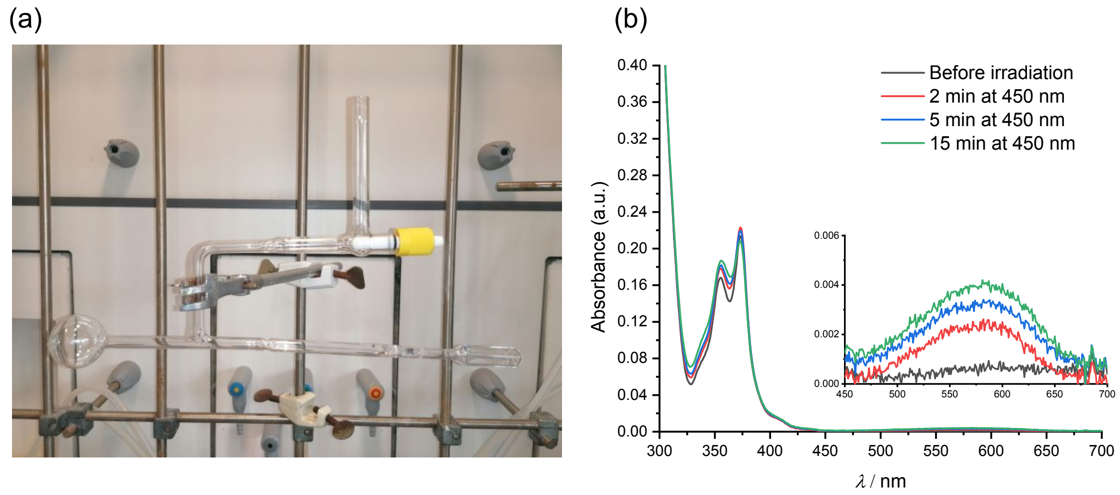

Figure S6. (a) The special quartz cuvette used for freeze-pump-thaw experiments. (b) Irradiation of **1** in toluene solution with 450 nm light *in the absence of diacetyl* (degassed by freeze-pump-thaw method).

It can be seen that photoreaction takes place (Figure S6b), but that the position of the emerging new bands highly resemble the new absorptions obtained when irradiation with 365 nm light was performed in the absence of diacetyl (see Figure 1 of the main text). The new absorption bands are not like the ones obtained when **1** was irradiated in the presence of diacetyl (see Figure 4 of the main text). Also, the intensity of change is very small. Based on these observations we believe that the exclusive formation of the ring-closed isomer in the case of triplet-sensitization experiments is caused by the triplet-sensitizer molecule.

The formation of side-product **9** in this control experiment may be caused by the non-monochromatic nature of the light source. The 450 nm LED light source might have a wider emission, resulting in small amounts of ~425 nm light, where **1** has a very small, but non-negligible, absorbance.

S2. ^{1}H NMR spectroscopic characterization of the photochemical transformations of 1

Compound **1** (~0.5 mg) was dissolved in C_6D_6 or CD_3CN (6-700 μL) and transferred to a 5 mm quartz NMR tube. The tube was sealed with a rubber septum, and the headspace of the NMR tube was purged with N_2 gas for 10-15 min. The NMR tube was then transferred to the NMR spectrometer, the sample was locked, tuned, shimmed and a spectrum was recorded. The sample was removed and irradiated with LED lamps with nominal emission maxima at 365, 450 and 590 nm. After each irradiation period, the sample was reshimmed and retuned before data acquisition. Sample spectra in CD_3CN are depicted in Figure S7.

The triplet-sensitization experiments were done similarly. Freshly distilled diacetyl (100 eq.) was added to the solution of compound **1** in C_6D_6. The solution was purged thoroughly with N_2 in a quartz NMR tube and sealed with a rubber septum.

Compound **1o** in C_6D_6 was irradiated with 365 nm UV light under N_2 atmosphere at rt. After 5 min two new sets of signals appeared, while the signals of compound **1o** decreased (Figure S7). After ~10 min of irradiation it was clear that the concentration of one of the species was constant while the other component started to accumulate in the solution (for quantitative analysis, see Figure S9). After 30 min of UV irradiation, the solution was exposed to visible light (590 nm) that resulted in the disappearance of the steady state product, while the concentration of the cumulative product did not change. These findings are in agreement with the UV-Vis studies that showed only partial reversibility after UV irradiation (Figure 1, see main text).

Exhaustive irradiation converted almost all starting material to the irreversible product. 2D NMR spectroscopy (HSQC, HMBC, Section S3) revealed that the irreversible photoproduct was not the closed form **1c** but an annulated side-product **9**. This structural motif has been identified as the product of a major side-reaction during the photochemical transformation of diarylethenes.[1] The formation of compound **9** type structures proceeds from the photochemically excited state of the closed form[1b,c] **1c**. Therefore, the steady state species is suggested to be the closed form **1c** of the dithienylbiphenylene switch. This is corroborated by the number of methyl signals in the ^{1}H NMR spectra (Figure S7): two with 1:1 integral ratio for the closed form **1c**, and three with 1:1:2 intensity for the side-product **9**. *Since visible light irradiation converted the closed form **1c** to the open form **1o**, we conclude that dithienylbiphenylene **1** exhibits reversible photochromism.*

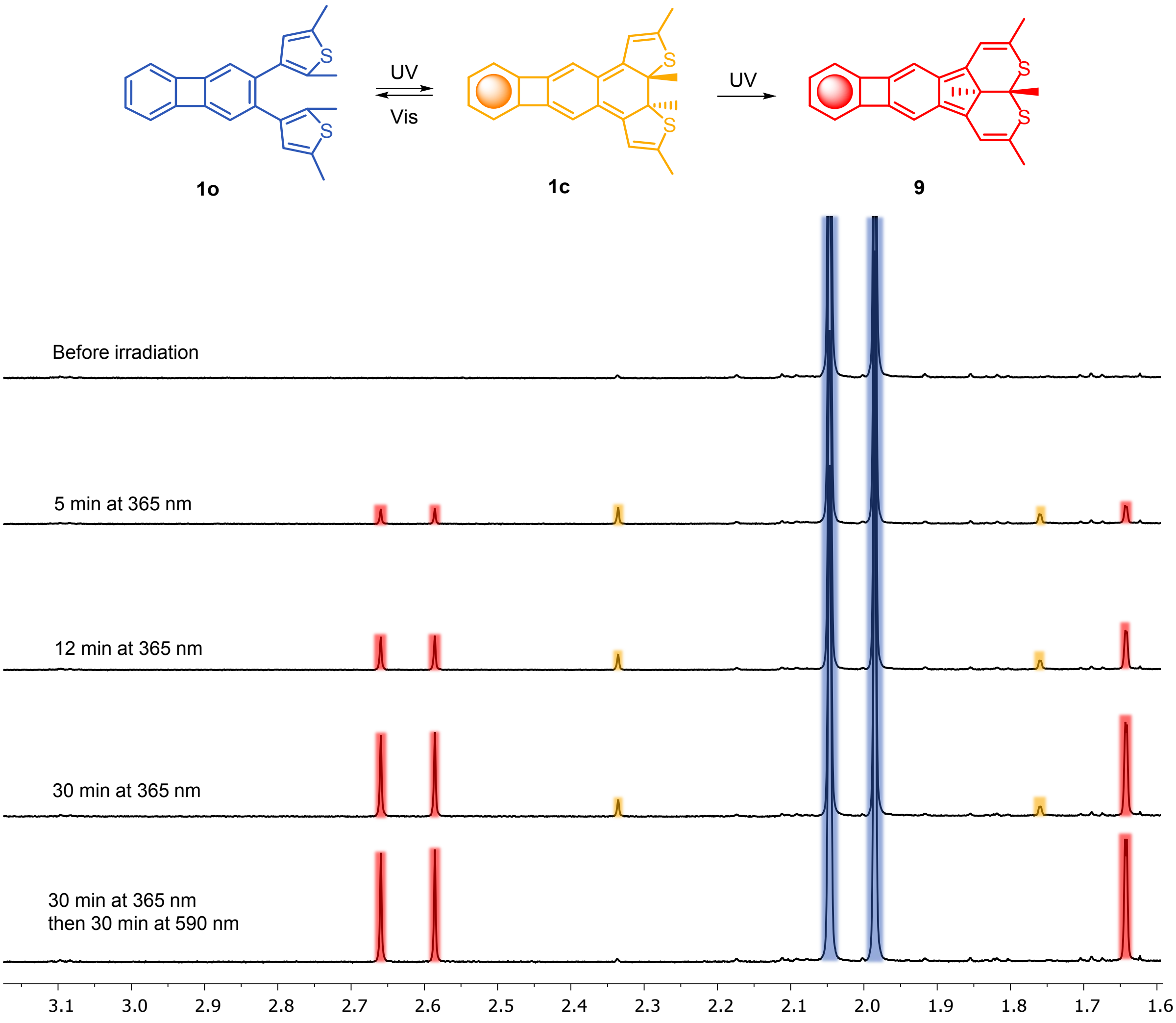

Figure S7. Irradiation of **1o** in C_6D_6 solution followed by ^1H NMR spectroscopy (ppm scale).

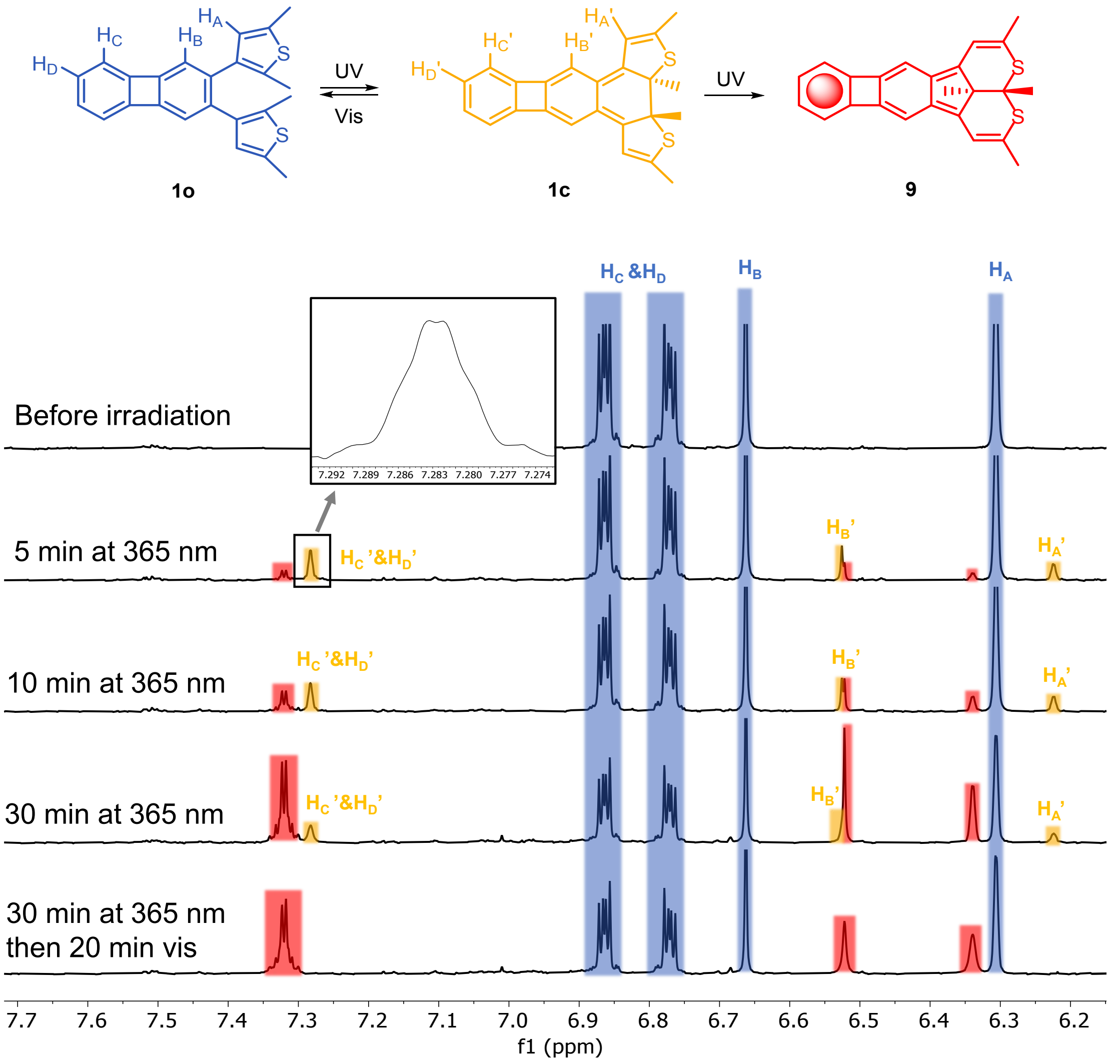

Figure S8. Irradiation of **1o** in CD_3CN solution followed by ^1H NMR (500 MHz) spectroscopy (ppm scale).

Quantitative interpretation of the ^{1}H NMR spectrum. A solution of **1o** in C_6D_6 was irradiated with 365 and 590 nm light, and the reaction was followed by ^1H NMR spectroscopy (Figure S7). The ^1H NMR spectra could be interpreted quantitatively (Figure S9). The integral of the methyl-signals of each species was used for quantization. The satellite peak of the residual C_6D_5H signal was used as an internal standard. A pulse angle of 45° and a relaxation time of 5 s were used to obtain the ^1H NMR spectra.

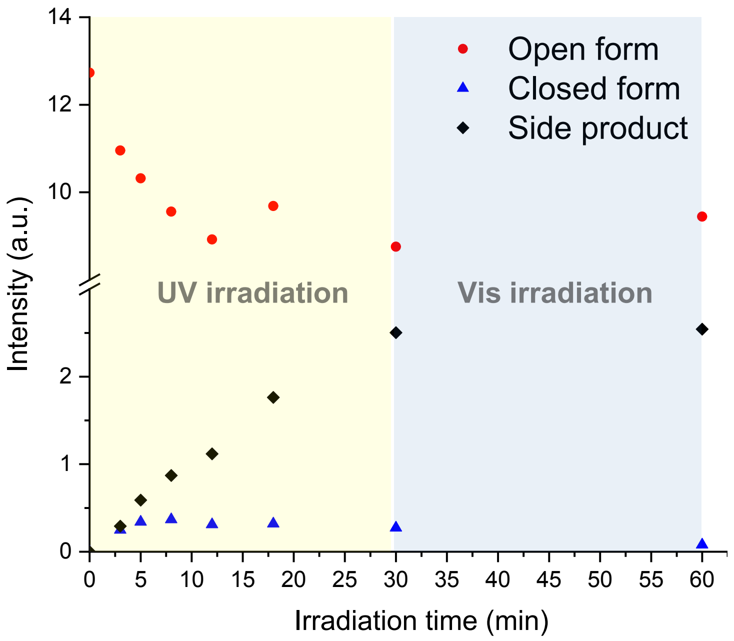

Figure S9. Relative concentration of the three species present in the irradiated sample of **1o** followed by ^{1}H NMR spectroscopy.

It can be seen that upon UV irradiation, the concentration of the open form rapidly decreases while the side-product starts to accumulate (Figure S9). During UV irradiation the concentration of the closed form is at steady state. When the UV irradiated sample was irradiated with visible light, the concentration of the open form increased, while the concentration of the closed form decreased significantly. The concentration of the side-product did not change significantly.

Triplet-sensitization experiment. To confirm the initial formation of the closed form **1c** we followed the sensitization experiment by ^{1}H NMR spectroscopy. After 5 min of irradiation only one new signal emerged in the methyl region (Figure S10). Based on the previous NMR experiments we could identify that this signal corresponds to the closed form **1c**, while the formation of the side-product **9** was not observed. Unfortunately, prolonged irradiation caused signal intensity loss, confirming the photodegradation of **1o** and **1c** in the presence of a triplet sensitizer.

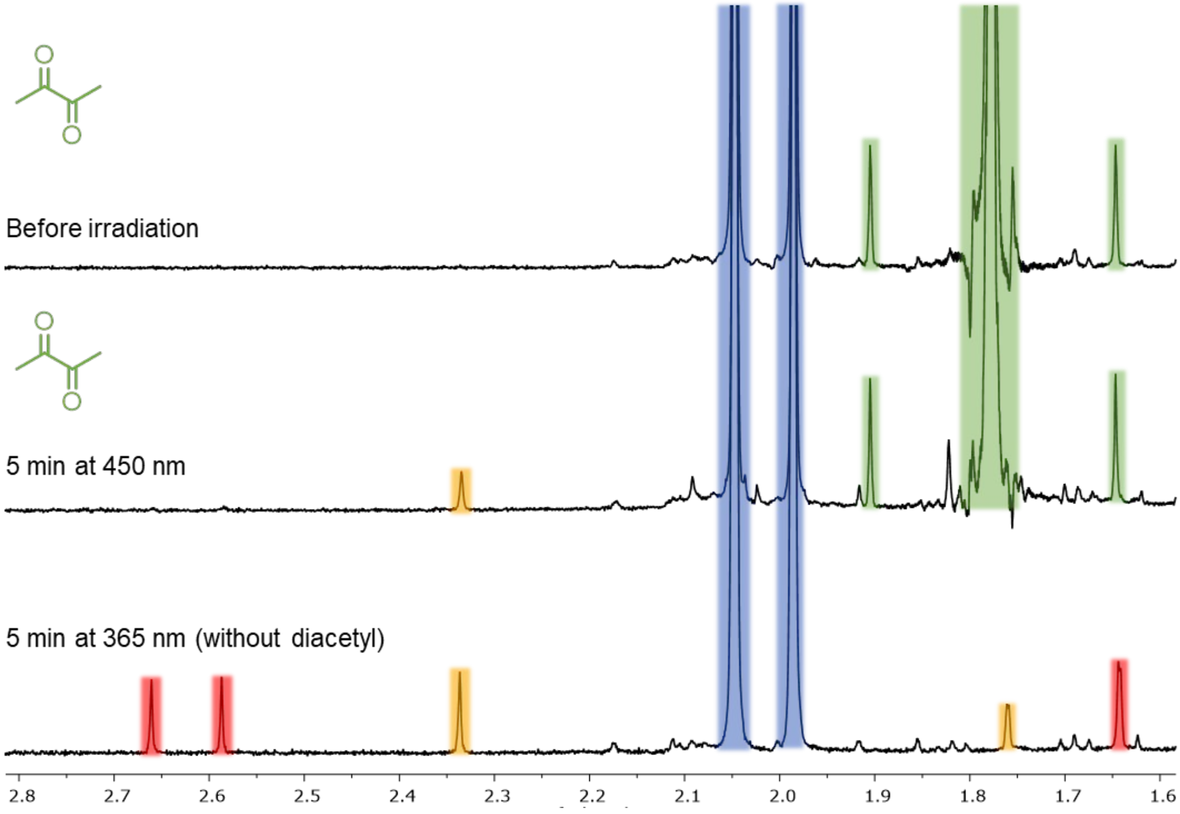

Figure S10. Irradiation experiments with and without diacetyl as triplet sensitizer followed by ^{1}H NMR spectroscopy in C_6D_6. Resonances of **1o** are highlighted in blue, resonances of **1c** are highlighted in orange, resonances of the side-product (**9**) are highlighted in red, while the methyl signals of diacetyl are highlighted in green.

S3. Isolation and characterization of side-product 9

A solution of **1** (C_6D_6) was irradiated for 5 hours with UV light (365 nm) in a quartz NMR tube under nitrogen atmosphere (Figure S11). After 5 hours the sample contained almost exclusively the side-product **9**. The sample was concentrated and transferred to a 3 mm NMR tube for further measurements (HSQC, HMBC). A ^{13}C NMR spectrum could not be obtained due to low concentration. ^{1}H NMR (500 MHz, C_6D_6) δ = 7.03 – 6.96 (m, 2H), 6.91 (dd, J = 5.3, 2.9 Hz, 2H), 6.23 (s, 2H), 5.99 ($d_{apparent}$, J = 1.4 Hz, 2H), 2.66 (s, 3H), 2.59 (s, 3H), 1.64 ($d_{apparent}$, J = 1.3 Hz, 6H) ppm.

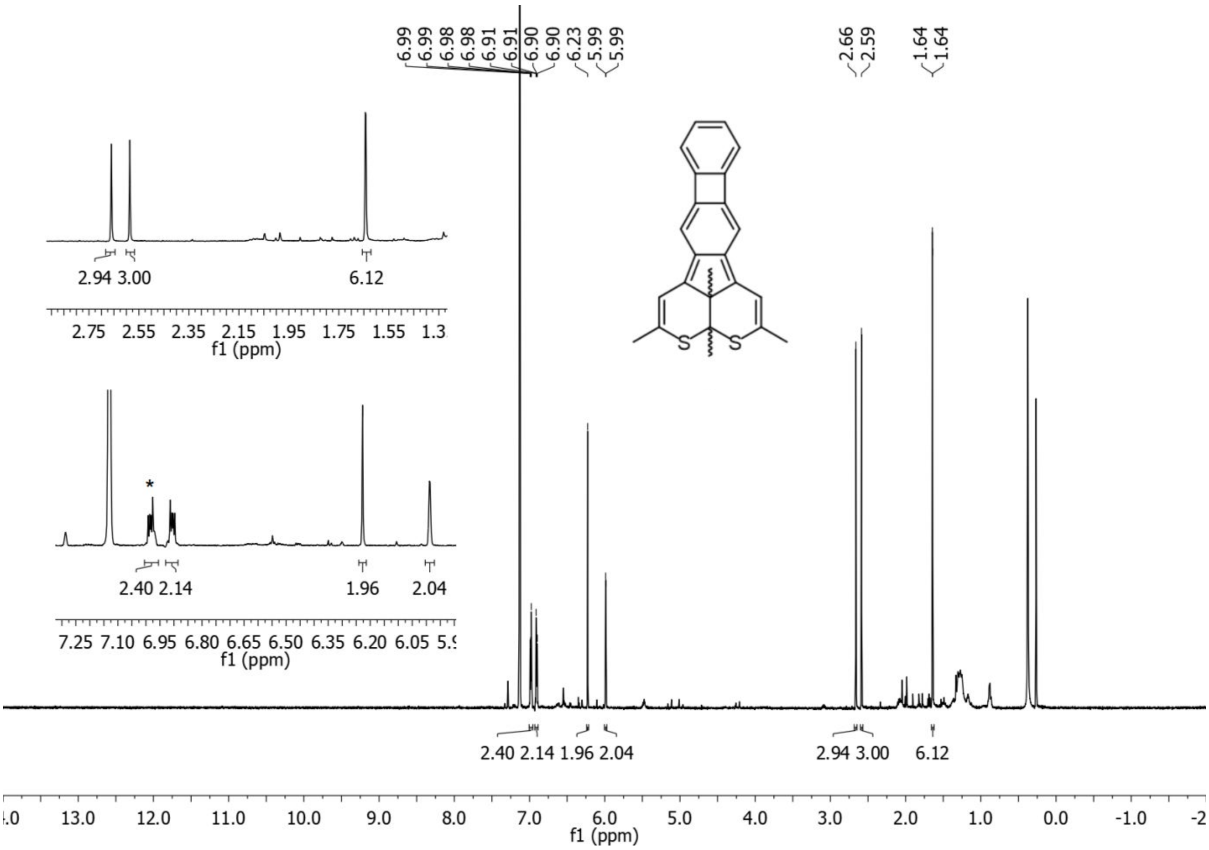

Figure S11. ^{1}H NMR spectrum of **9** in C_6D_6 (500 MHz). The spectrum was recorded in a 3 mm NMR tube. (*- the satellite peak of the residual C_6D_5H peak overlaps with the signal).

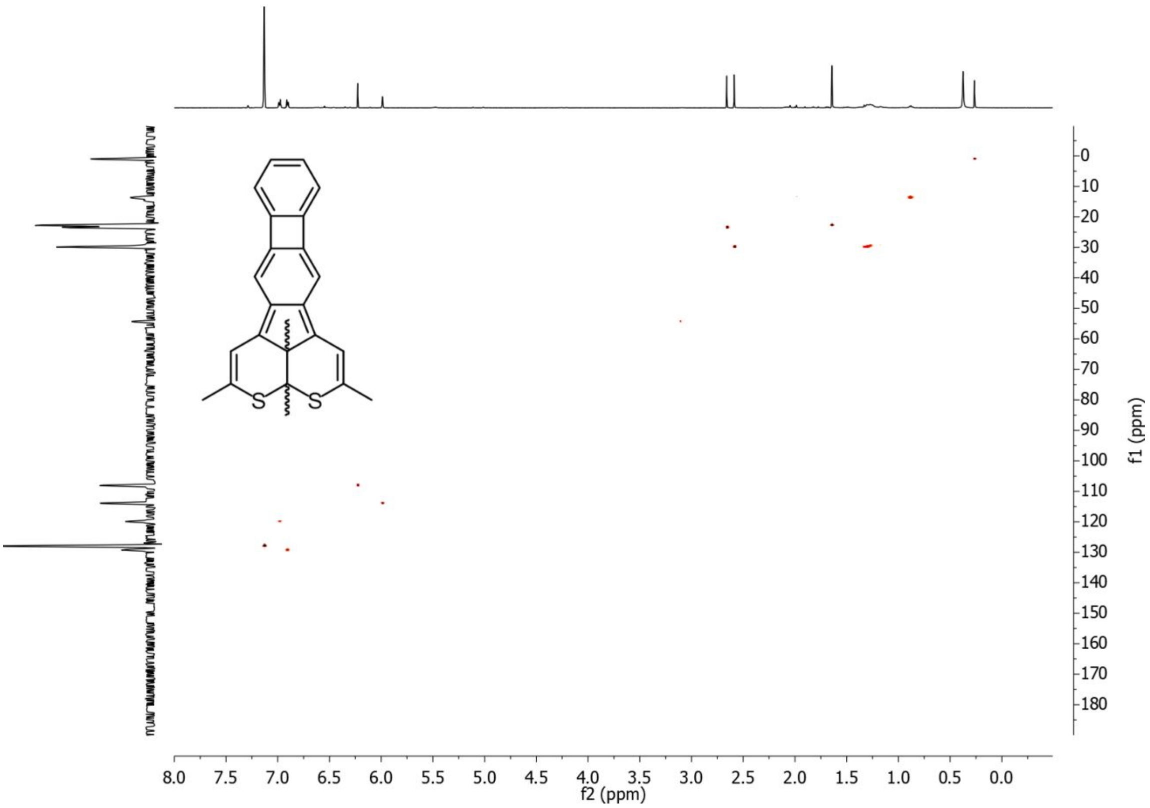

Figure S12. $^{1}H - ^{13}C$ HSQC spectrum of **9** in C_6D_6.

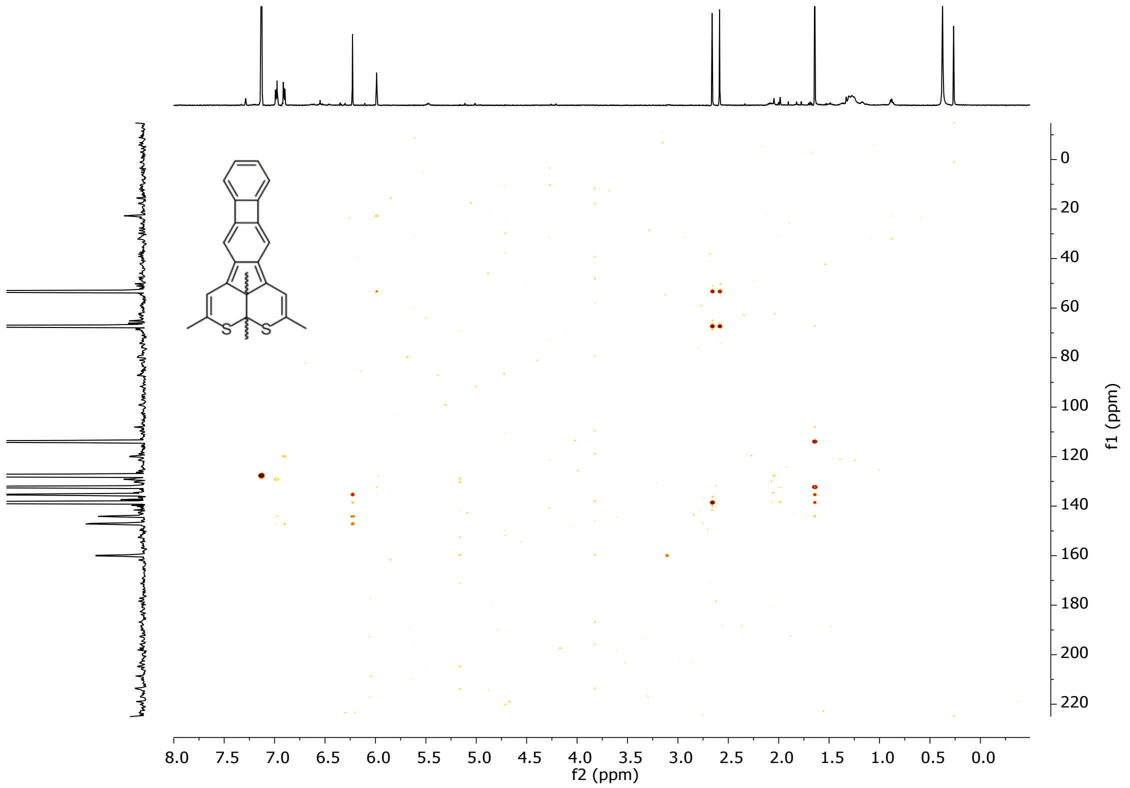

Figure S13. $^{1}H - ^{13}C$ HMBC spectrum of **9** in C_6D_6.

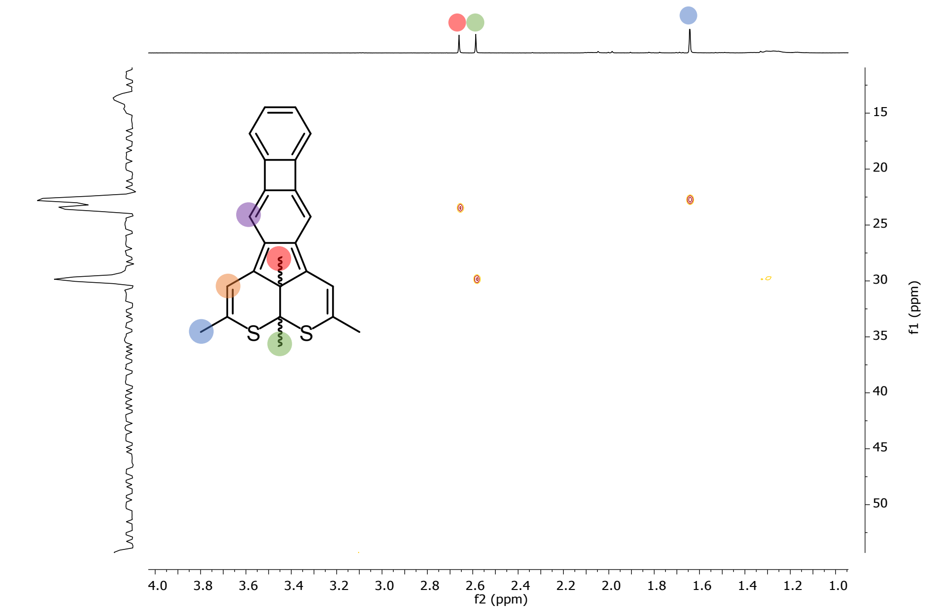

Figure S14. The methyl region of the ^{1}H – ^{13}C HSQC spectra of **9** in C_6D_6. C/H pairs were assigned based on the ^{1}H – ^{13}C HSQC and HMBC spectra.

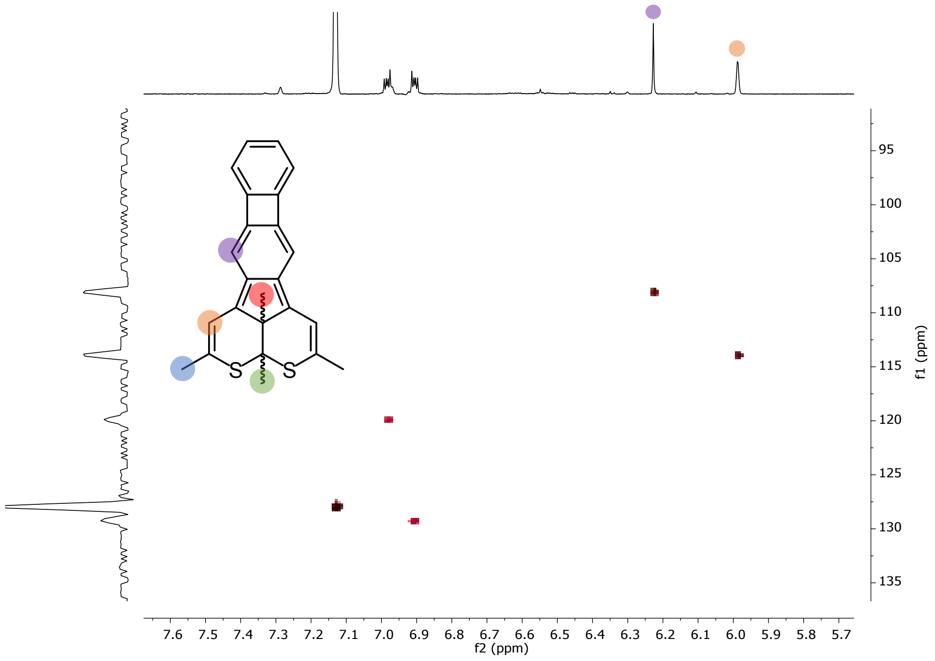

Figure S15. The aromatic region of the ^{1}H – ^{13}C HSQC spectra of **9** in C_6D_6. C/H pairs were assigned based on the ^{1}H – ^{13}C HSQC and HMBC spectra.

S4. UV-Vis spectroscopic characterization of 10

UV-Vis spectrophotometry was executed on a Perkin-Elmer Lambda 465 spectrophotometer. Hellma Analytics High Precision quartz cuvettes were used with optical path length of 1.0 cm. Irradiation of samples was carried out with LED lamps with nominal emission maxima at 365 and 620 nm in Hellma Analytics High Precision quartz cuvettes. The irradiation experiments summarized in Figure 6 (see main text) and Figure S17 were performed under N_2 atmosphere. The irradiation experiments summarized in Figure S18 and S19 were performed under air.

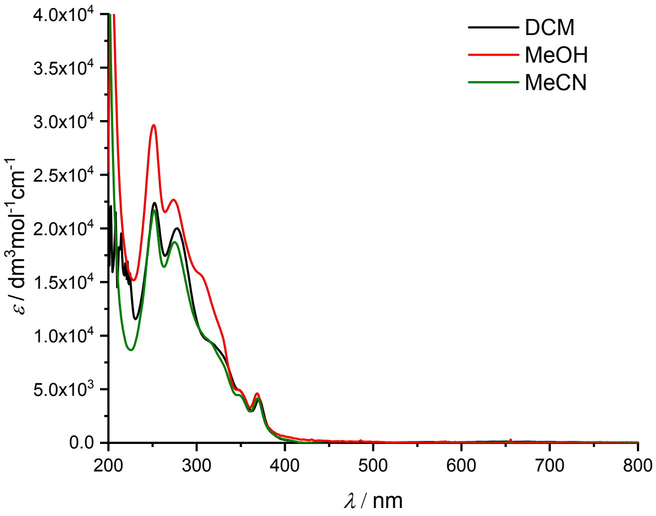

Figure S16. UV-Vis spectra of **10o** in different solvents (c = 2.72×10^{-5} M, rt).

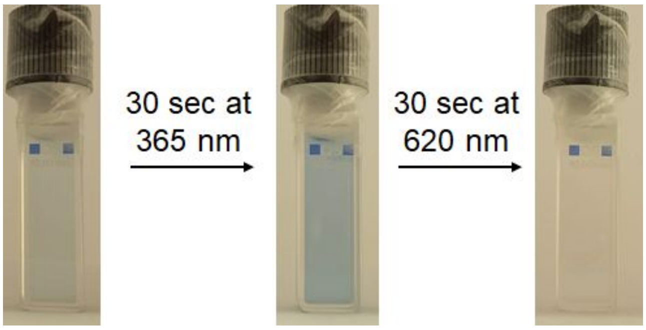

Figure S17. Solution of **10** in CH_3CN before irradiation, after UV irradiation, and after visible light irradiation. It can be seen that the initial solution contains a small amount of the closed form, which can also be deduced from the UV-Vis spectra in Figure 5 (see main text).

Irradiation of 10o in different solvents followed by UV-Vis spectroscopy. In all cases the initial solutions were pre-irradiated with 620 nm light for 30 s to transform residual closed form to the open form. Irradiation experiments were performed without the exclusion of air.

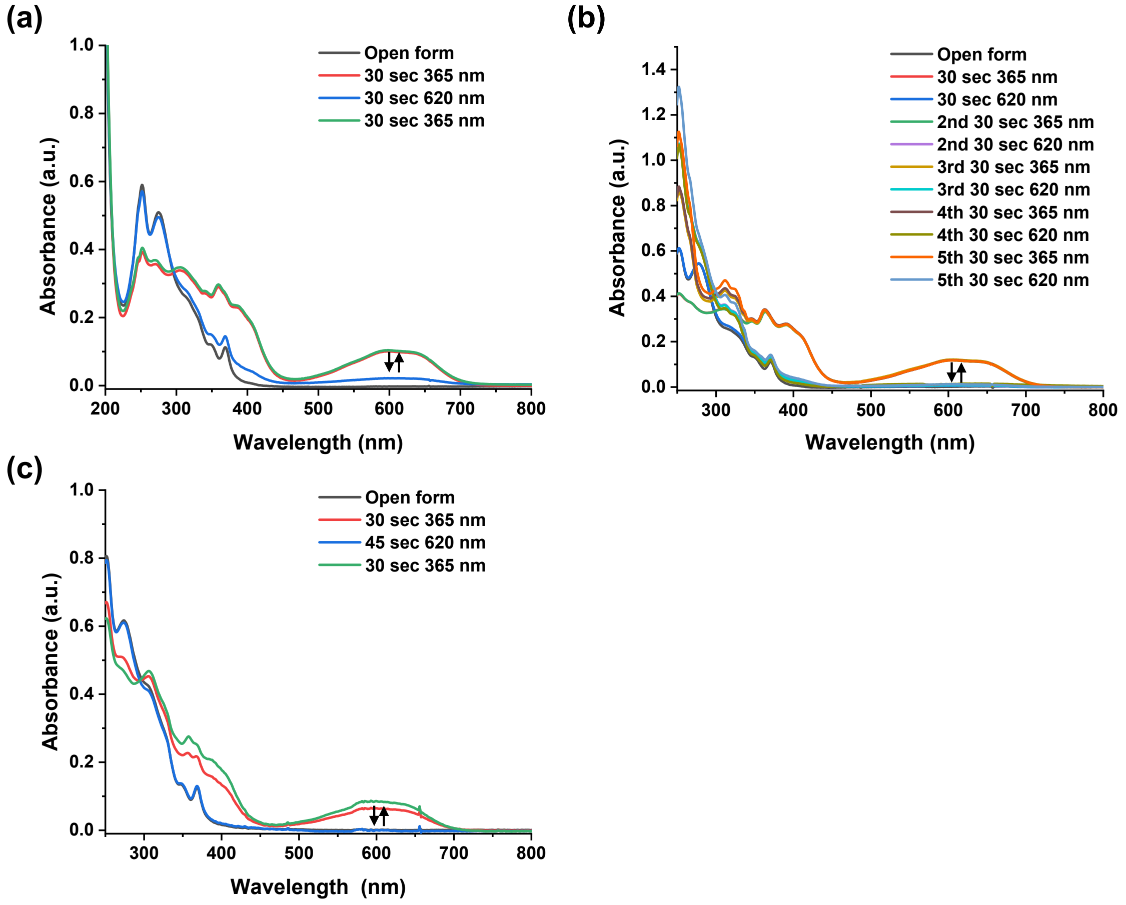

Figure S18. Irradiation of **10o** in (a) CH_3CN, (b) DCM and (c) MeOH followed by UV-Vis spectroscopy (c = approx. 3×10^{-5} M, rt).

S5. ^{1}H NMR spectroscopic characterization of the photochemical transformations of 10

In a control experiment to track the formation of **10c** from **10o** under ambient light, a sample of **10** was subjected to column chromatography (SiO_2, hexane/ethyl-acetate 12:1) in the dark to isolate **10o**. After TLC analysis of the fractions in the dark, a portion of the fractions containing **10o** was treated in the dark, while another portion was treated without the exclusion of light. While no significant difference could be observed between the ^{1}H NMR spectra of the two samples, it is clear (Figure S19) that the sample that was treated under ambient light is dark blue coloured while the sample that was treated in the dark has a light green colour. This is probably due to a minute amount of **10c** formed.

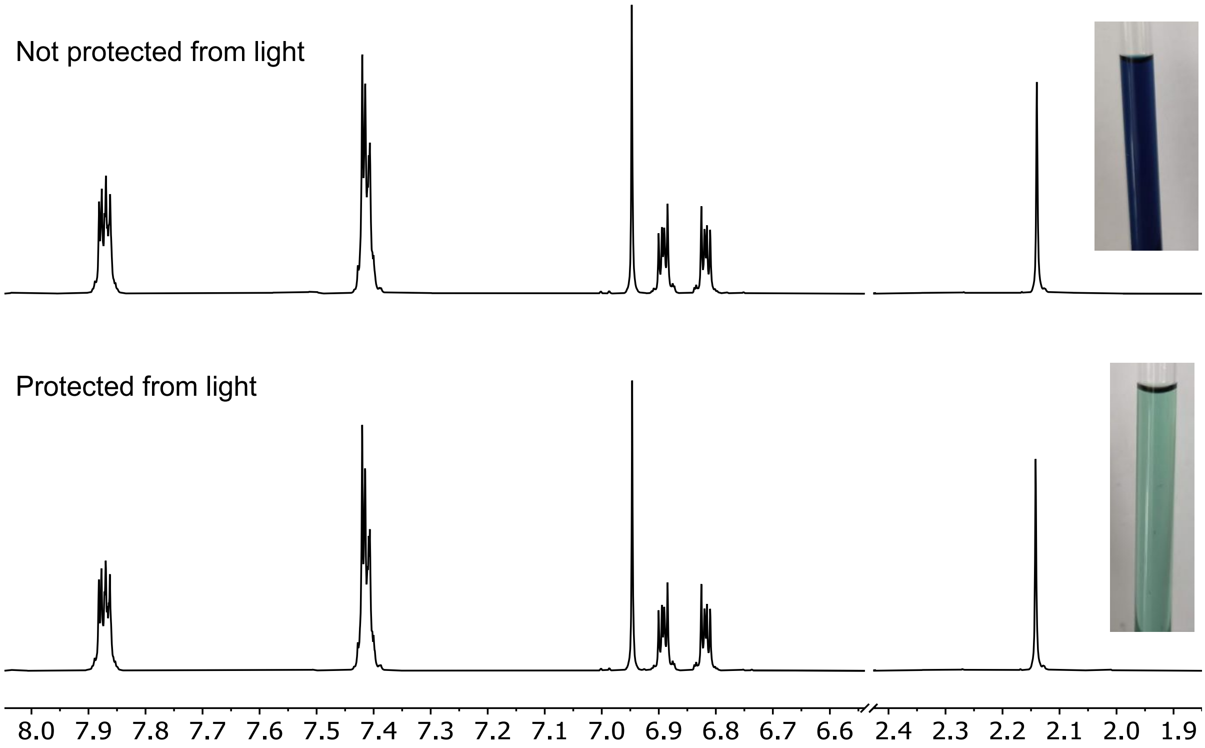

Figure S19. Control experiment to track the formation of **10c** from **10o** under ambient light. ^{1}H NMR spectra (500 MHz) were recorded in CD_2Cl_2 at 30 °C and shown in ppm scale. For better visibility, the intensity is lower for the methyl region.

Determination of the photostationary state. The composition of compound **10** at the photostationary state (PSS) in CD_2Cl_2 and C_6D_6 was determined by ^1H NMR spectroscopy and integration. The results are summarized in Table S1 and in Figures S20–S22. Attempts were made to also determine the PSS composition in CH_3CN (both by ^1H NMR and HPLC experiments). However, in this solvent, prolonged exposure (1 hour) to UV light resulted in the photodegradation of **10**.

Table S1. PSS Composition of 10o/10c in Different Solvents

solvent	irradiation time (min)	PSS composition **10o**/**10c**
CD_2Cl_2	95	1:1.6
C_6D_6	160	1:4.8

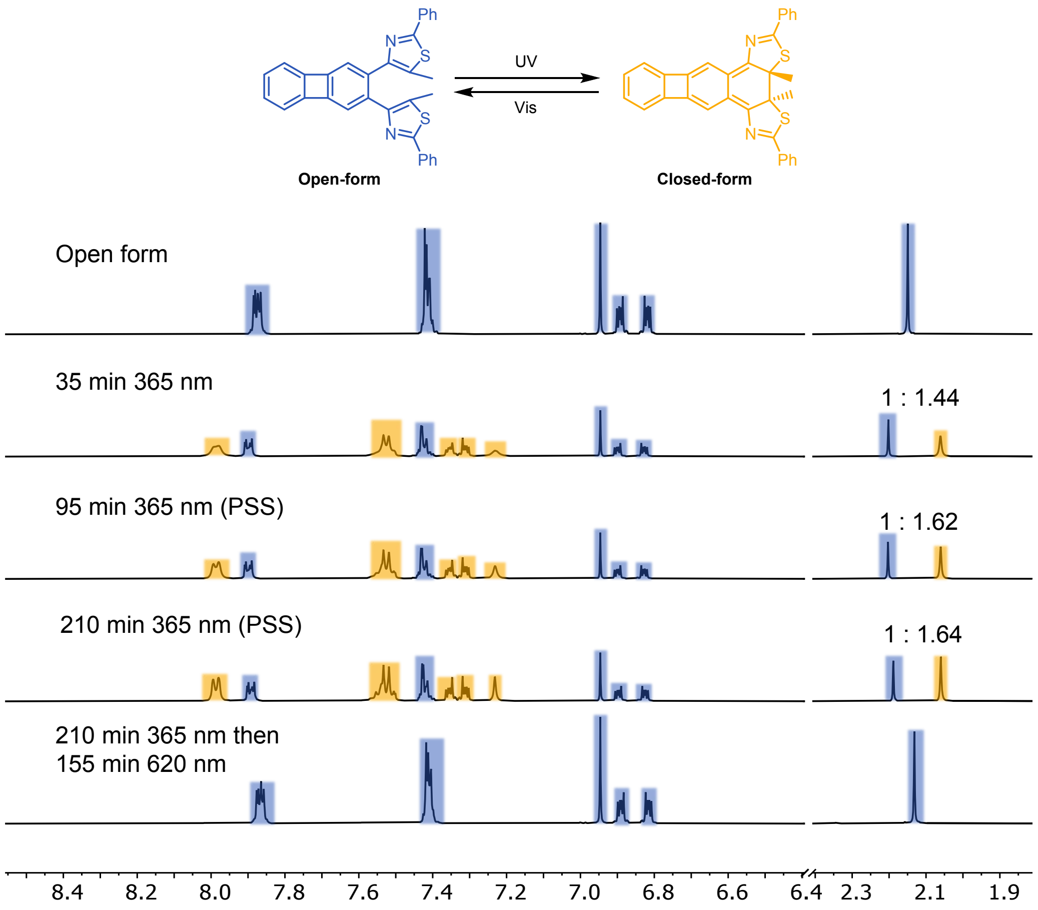

Figure S20. Irradiation of pure **10o** in CD_2Cl_2 followed by ^1H NMR spectroscopy (ppm scale). The PSS was reached after 95 min of UV irradiation, and **10c** could be completely converted back to **10o**. For better visibility, the intensity is lower for the methyl region.

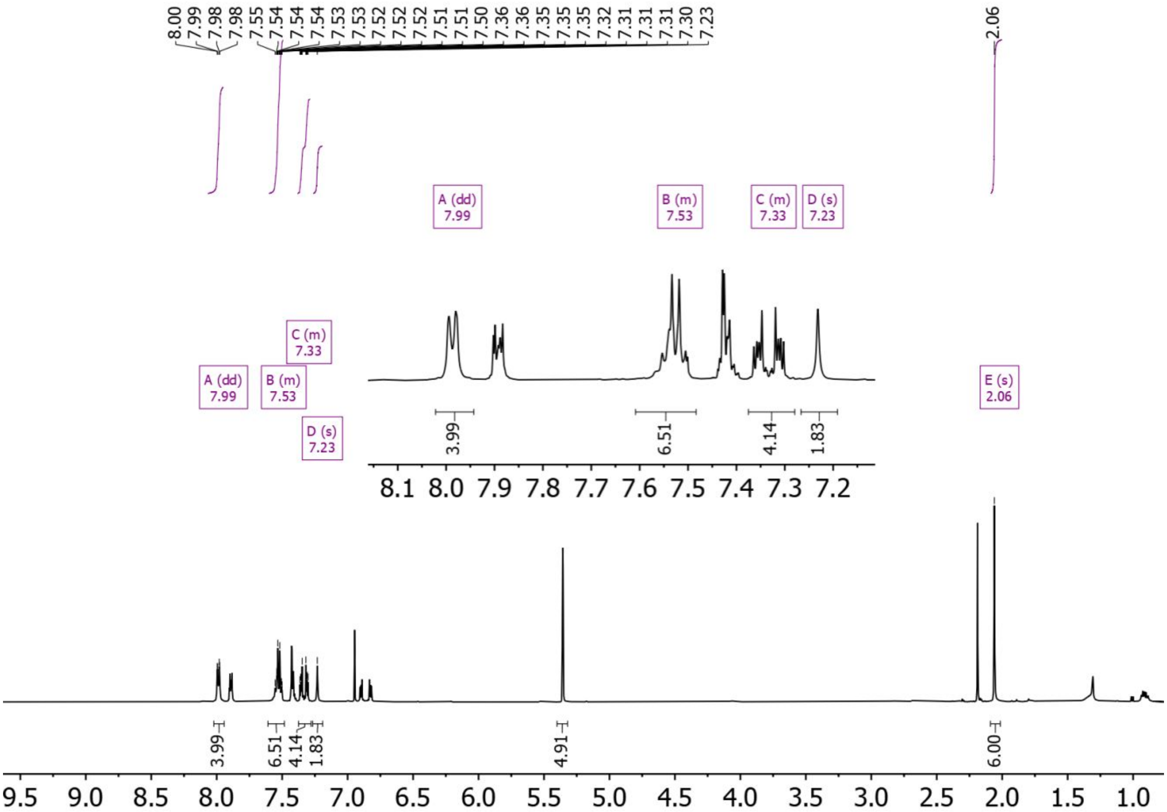

Figure S21. ^{1}H NMR (500 MHz) spectrum of the PSS mixture in CD_2Cl_2. The ^{1}H NMR shifts of **10c** are highlighted in the inset.

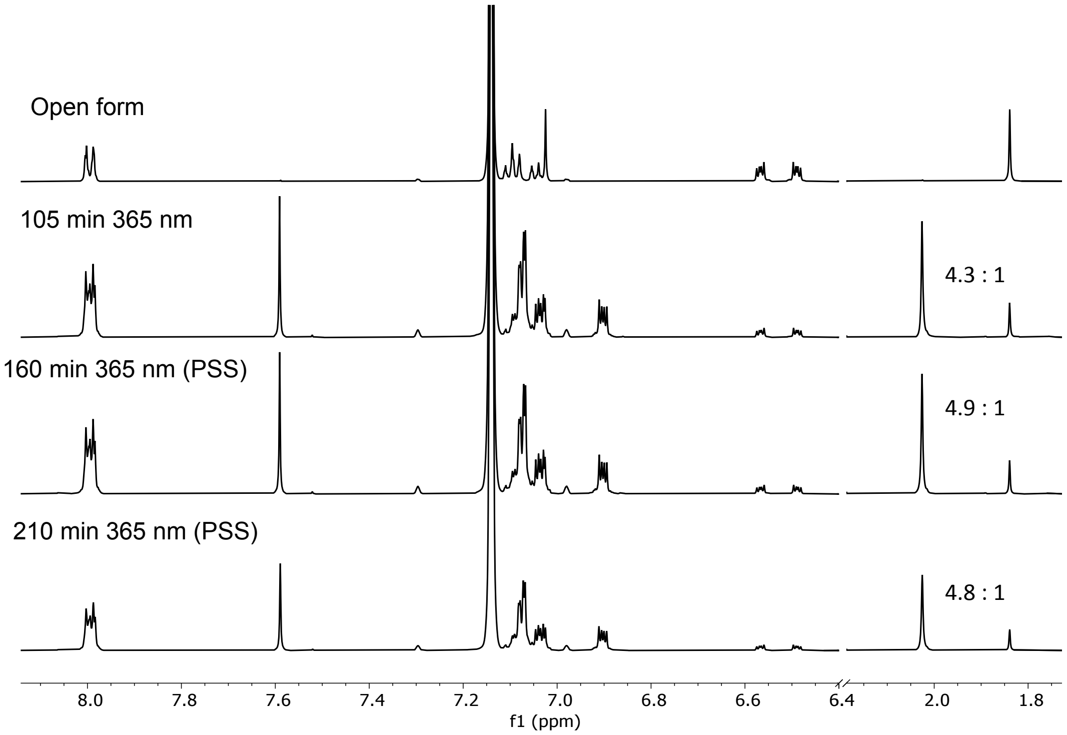

Figure S22. ^{1}H NMR (500 MHz) spectrum of the PSS mixture in C_6D_6. For better visibility, the intensity is lower for the methyl region.

S6. Thermal stability of 10c

The thermal stability of **10c** was assessed by UV-Vis and ^{1}H NMR spectroscopic measurements.

UV-Vis experiments. A sample containing the PSS composition of **10o** and **10c** was generated by UV light irradiation (5 min at 365 nm), then the sample was heated in an aluminium block in the dark for 1 hour at 110 °C. Another sample that was prepared identically was stored in the dark at room temperature for 1 hour. The results are summarized in Figure S23.

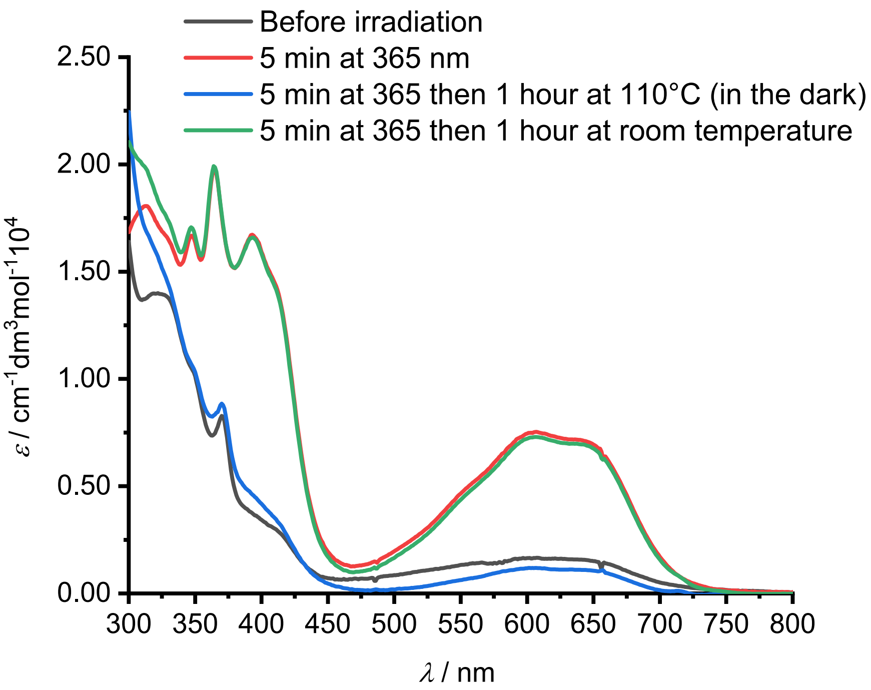

Figure S23. Irradiation of **10** in toluene (c = 2.86×10^{-5} M) followed by thermal stability experiments at 110 °C.

It is clear from Figure S23 that while the UV-Vis spectrum of the sample that was left at room temperature (in the dark) did not change significantly, that of the heated sample is very similar to that before irradiation. This shows that, at 110 °C, **10c** is thermally unstable and undergoes cycloreversion to produce **10o**.

NMR experiments. In order to gain quantitative information on the thermal cycloreversion of **10c** into **10o**, samples containing photochemically generated mixtures of the two species in C_6D_6 were heated in an NMR magnet at two different temperatures (70 and 90 °C). The thermal

cycloreversion process was followed by ^{1}H NMR spectroscopy. A sample series of spectra of the time-course measurement is presented in Figure S24.

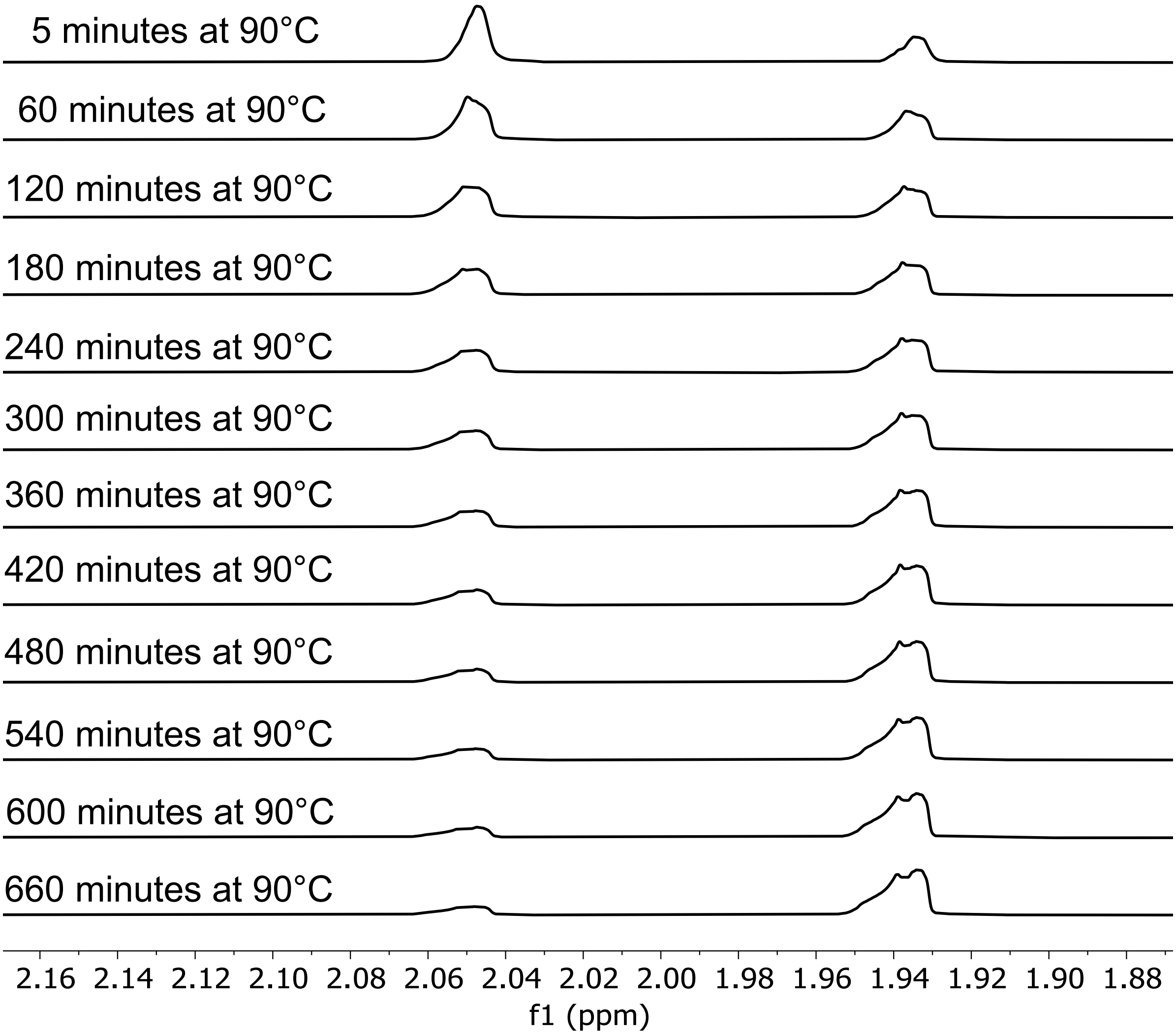

Figure S24. Methyl region of the ^{1}H NMR (500 MHz) spectrum of **10** at 90 °C in C_6D_6 at different thermolysis times.

Integration of the absorptions of the methyl groups of **10o** and **10c** allowed us to extract kinetic data for the cycloreversion at both temperatures (Figures S25 and S26). The data points were fitted to exponential functions of the form $c = ae^{bt}$, where a and b are parameters, c is the relative intensity, and t is time (Figures S25 and S26).

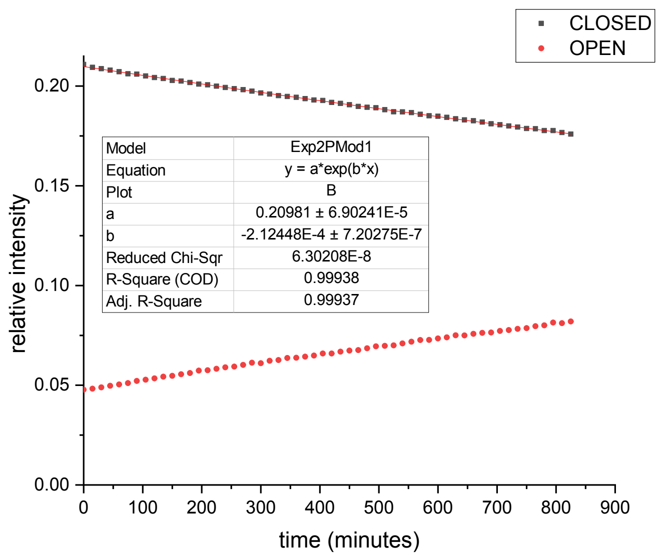

Figure S25. Changes in the relative intensities of the methyl signals from **10o** an **10c** at 70 °C based on ^{1}H NMR (500 MHz) integration. A sample containing the PSS composition of **10o**/**10c** was used for the experiment.

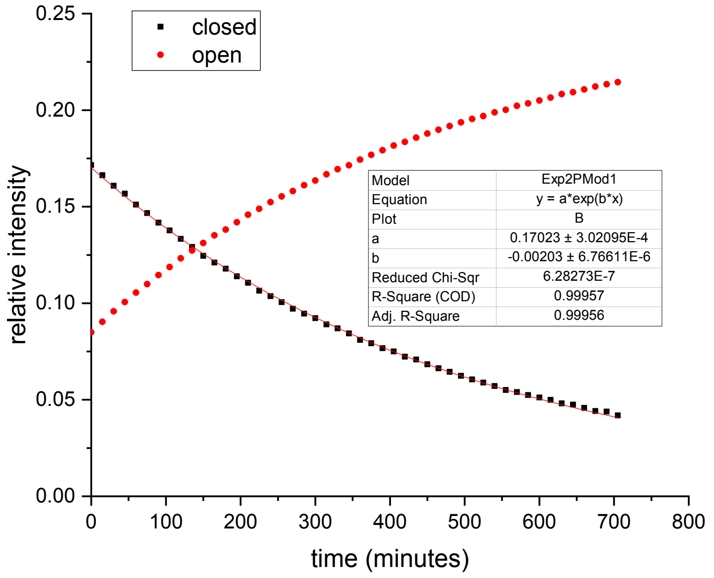

Figure S26. Changes in the relative intensities of the methyl signals from **10o** an **10c** at 90 °C based on ^{1}H NMR (500 MHz) integration. The sample that was previously heated at 70 °C was used for the experiment.

Assuming that the cycloreversion is a first-order process, the corresponding rate constants at the two temperatures were calculated from parameter b of the exponential functions in Figures S25 and S26, according to equations 1 and 2. Furthermore, from these rate constants, the corresponding thermal half-lives were calculated according to equations 3 and 4. The results are summarized in Table S2.

$$k(70\ °\mathrm{C}) = \frac{-b(70\ °\mathrm{C})}{60} = 3.5 \cdot 10^{-6}\ \mathrm{s}^{-1} \tag{1}$$

$$k(90\ °\mathrm{C}) = \frac{-b(90\ °\mathrm{C})}{60} = 3.4 \cdot 10^{-5}\ \mathrm{s}^{-1} \tag{2}$$

$$t_{1/2}(70\ °\mathrm{C}) = \frac{\ln(2)}{k} = \frac{\ln(2)}{3.54 \cdot 10^{-6}} = 54\ \mathrm{h} \tag{3}$$

$$t_{1/2}(90\ °\mathrm{C}) = \frac{\ln(2)}{k} = \frac{\ln(2)}{3.38 \cdot 10^{-5}} = 5.7\ \mathrm{h} \tag{4}$$

Table S2. Kinetic Data for the Thermal Cycloreversion of 10c into 10o

T (°C)	k (s^{-1})	$t_{1/2}$ (h)
70	$3.5 \cdot 10^{-6}$	54
90	$3.4 \cdot 10^{-5}$	5.7

Assuming that the activation energy (E_a) for the cycloreversion process is independent of temperature, the Arrhenius equation was then used to estimate a value of 117 kJ mol^{-1} for this quantity:

$$k = Ae^{-\frac{E_a}{RT}} \tag{5}$$

$$\ln\left(\frac{k_1}{k_2}\right) = \frac{E_a}{RT_2} - \frac{E_a}{RT_1} \tag{6}$$

$$E_a = \frac{R\ln\left(\frac{k_1}{k_2}\right)}{\frac{1}{T_2} - \frac{1}{T_1}} = \frac{8.314\ln\left(\frac{3.5\ \cdot\ 10^{-6}}{3.4\ \cdot\ 10^{-5}}\right)}{\frac{1}{363.15} - \frac{1}{343.15}} = 117\ \mathrm{kJ\ mol}^{-1} \tag{7}$$

As we will see below, this value agrees quite well with that of 104.8 kJ mol^{-1} obtained from the quantum chemical calculations. Finally, it should be noted that no thermal decomposition or side-product formation was observed in these experiments.

Experimental procedure for thermokinetic measurements. A solution of compound **10** in C_6D_6 was added to a screw cap NMR tube, and was irradiated with UV light for 3 hours. Internal standard (1,3,5-trimethoxybenzene) was added and a time-course measurement was performed at 70 °C for 14 hours (sampling an ^{1}H NMR spectrum every 15 min). Following the experiment at 70 °C, the temperature of the NMR was increased to 90 °C and another time-course measurement was started for 12 hours (again sampling an ^{1}H NMR spectrum every 15 min). The resulting concentration time profiles were fitted using OriginLab.

S7. Computational details and complementary computational results

Geometries and NICS indices. The geometries of **1o**/**1c** and **10o**/**10c** and of the transition structures (ts) that mediate their thermal electrocyclization and cycloreversion reactions were optimized using the B3LYP hybrid density functional in combination with the cc-pVTZ basis set and the SMD continuum solvation model[2] to describe the toluene solvent employed for many of the irradiation experiments. Based on the resulting geometries, which are displayed in Figure S27 and show C1–C1' distances of 3.72 (**1o**), 1.99 (**1ts**) and 1.54 Å (**1c**) and of 3.64 (**10o**), 1.99 (**10ts**) and 1.54 Å (**10c**), frequency calculations were performed at the same level of theory. These calculations helped ensure that the resulting structures are either potential-energy minima with real vibrational frequencies only (**1o**/**1c** and **10o**/**10c**), or first-order saddle points with one imaginary vibrational frequency along the electrocyclization/cycloreversion reaction coordinate (**1ts** and **10ts**). The reason why it is of interest to locate the latter structures is the possibility to thereby predict the free-energy barriers for the thermal electrocyclization and cycloreversion reactions of **1o**/**1c** and **10o**/**10c**. Specifically, if these barriers are high, it might be worthwhile to consider the photocyclization reactions of **1o** and **10o** for applications in molecular solar thermal energy (MOST) storage.[3]

Using the geometries of **1o**/**1c** and **10o**/**10c**, nucleus-independent chemical shift (NICS) indices[4] were calculated at the B3LYP/cc-pVTZ/SMD level of theory by means of a NICS-scan procedure.[5] Specifically, employing gauge-including atomic orbitals, so-called $NICS_{zz}$ values[4b] corresponding to the negative of the zz-component of the magnetic shielding tensor were calculated for each of the three rings (***A***, ***B*** and ***C***) of the biphenylene moiety at distances ranging from 1.50 to 2.00 Å (in steps of 0.10 Å) above the geometric center of the respective ring. The rationale for choosing this scan interval is to minimize the contributions from σ-electrons to the induced magnetic field, which tend to be larger at distances proximate to the molecular plane.[6] While the full set of calculated $NICS_{zz}$ values is provided in Table S3, the discussion of these results focuses on the values obtained at 1.70 Å, which is the distance that Gershoni-Poranne and Stanger[5b] recommend for calculations of this kind. The corresponding results are summarized in Figure 3 of the main text and in Figure S28.

All geometry optimizations, frequency calculations and NICS-index calculations were performed with the Gaussian 16 suite of programs.[7]

HOMA and SA indices. The harmonic oscillator model of aromaticity (HOMA)[8] index is a geometric aromaticity index that probes the deviation of the carbon-carbon bond lengths R_i of the conjugated ring system in question from an ideal R_{opt} value associated with the fully aromatic benzene molecule. It is defined as

$$\text{HOMA} = 1 - \frac{\alpha}{n}\sum_i^n (R_i - R_{\text{opt}})^2,$$

where n is the number of carbon-carbon bonds in the ring and α is an empirical normalization factor chosen in such a way that the HOMA value approaches 1, 0 and -1 for an aromatic, non-aromatic and antiaromatic system, respectively.[8] In this work, the standard parameters $\alpha = 257.7$ Å^{-2} and $R_{\text{opt}} = 1.388$ Å were employed to calculate HOMA values using the geometries optimized at the B3LYP/cc-pVTZ/SMD level of theory.

The Shannon aromaticity (SA)[9] index, in turn, is an electronic aromaticity index that probes the variation in electron density at bond critical points (BCPs) of the conjugated ring. Specifically, this index is formulated in terms of the so-called Shannon entropy

$$S = -\sum_i^N p_i(\mathbf{r}_\text{c}) \ln p_i(\mathbf{r}_\text{c}),$$

where $\mathbf{r}_\text{c}$ is a BCP, N is the number of BCPs, and $p_i(\mathbf{r}_c)$ is the normalized probability electron density at a given BCP. Using this definition, the SA index is obtained as the difference between the Shannon entropy that the system would have if it was perfectly aromatic, and the Shannon entropy that it *actually* has. Accordingly, aromatic systems typically show SA values below 0.003, whereas antiaromatic systems often have SA values above 0.005 (the former exhibit smaller variations in electron density at BCPs).[9] In this work, SA values were derived from the electron densities of the B3LYP/cc-pVTZ/SMD-optimized geometries.

All HOMA- and SA-index calculations were performed using the Multiwfn program.[10]

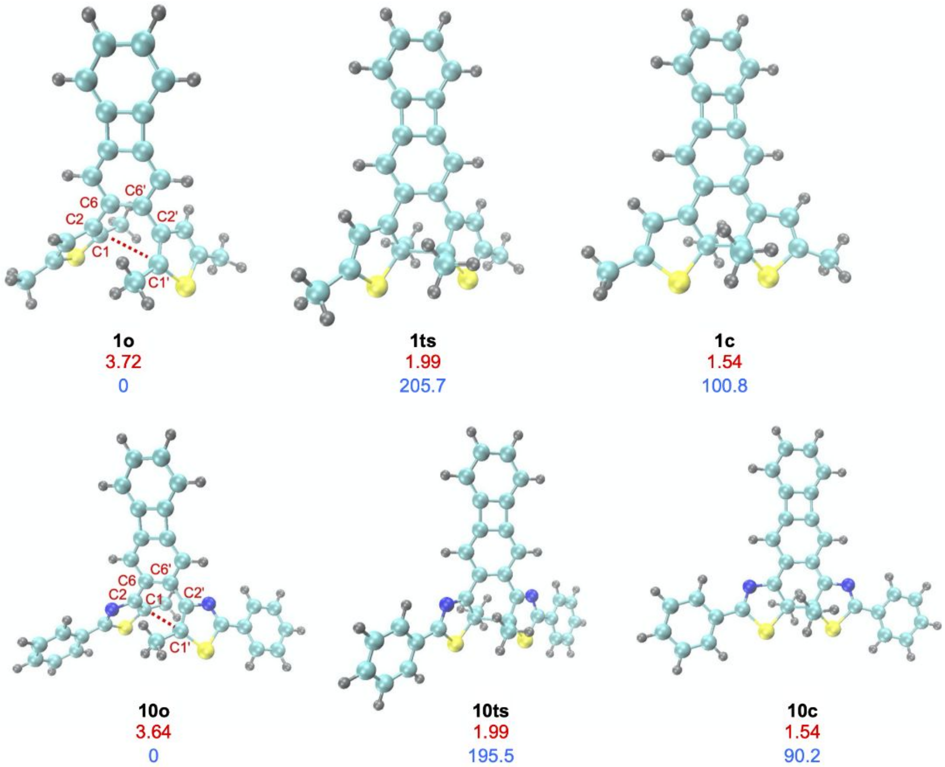

Figure S27. Optimized geometries of the open and closed isomers of **1** and **10** and the transition structures (**1ts** and **10ts**) that connect them. Values in red font are the corresponding C1–C1' distances (Å) and values in blue font are the corresponding relative free energies (kJ mol^{-1}).

Table S3. NICS$_{zz}$ Values (ppm) for Rings *A*–*C* of the Open and Closed Isomers of 1 and 10 Calculated at Different Distances (Å) Above the Geometric Ring Centers

		distance						
species	ring	1.50	1.60	1.70	1.80	1.90	2.00	<NICS$_{zz}$>[a]
**1o**	***A***	−9.6	−9.2	−8.7	−8.2	−7.7	−7.2	−8.4
	B	12.5	9.6	7.3	5.4	3.9	2.6	6.9
	C	−12.1	−11.6	−11.0	−10.4	−9.7	−9.1	−10.6
**1c**	***A***	−1.1	−1.7	−2.1	−2.4	−2.6	−2.7	−2.1
	B	−1.8	−2.7	−3.3	−3.8	−4.1	−4.4	−3.3
	C	−19.5	−18.4	−17.3	−16.1	−15.0	−14.0	−16.7
**10o**	***A***	−7.7	−7.3	−6.9	−6.5	−6.0	−5.6	−6.7
	B	11.5	9.0	7.0	5.3	3.9	2.8	6.6
	C	−10.8	−10.3	−9.8	−9.2	−8.6	−8.0	−9.4
**10c**	***A***	−1.3	−1.9	−2.2	−2.5	−2.7	−2.8	−2.2
	B	−0.7	−1.7	−2.5	−3.1	−3.5	−3.8	−2.6
	C	−19.1	−18.0	−16.9	−15.8	−14.7	−13.7	−16.4

[a]The mean NICS$_{zz}$ value over the different distances above the geometric ring center.

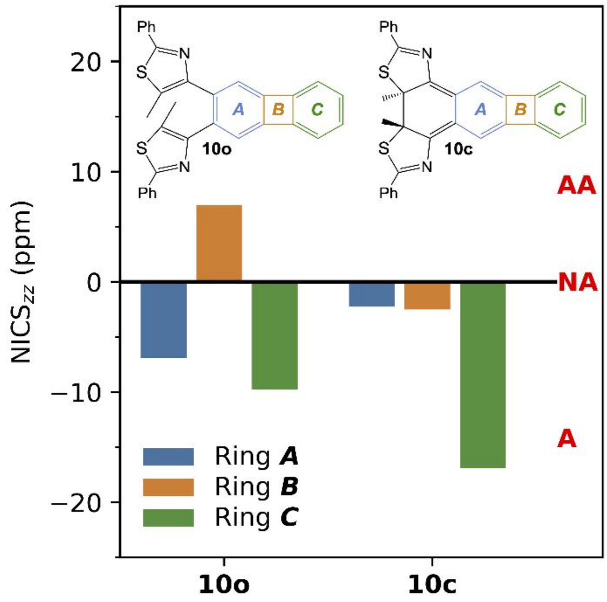

Figure S28 NICS$_{zz}$ values for rings *A*–*C* of **10o** and **10c** calculated 1.70 Å above the geometric ring centers. (A: aromatic; WA: weakly aromatic; AA: antiaromatic; NA: non-aromatic).

Table S4. HOMA and SA Values for Rings *A–C* of the Open and Closed Isomers of 1 and 10

	HOMA				SA/10^{-3}			
species	ring ***A***	ring ***B***	ring ***C***	ring ***A-B-C***	ring ***A***	ring ***B***	ring ***C***	ring ***A-B-C***
**1o**	0.76	−0.85	0.87	0.34	1.3	6.2	0.6	3.7
**1c**	−0.32	−0.60	0.93	0.09	6.5	2.4	0.2	4.6
**10o**	0.76	−0.84	0.87	0.34	1.3	6.0	0.6	3.8
**10c**	−0.19	−0.60	0.94	0.14	5.9	2.5	0.2	4.3

Table S5. Calculated Free Energies and Activation Energies (kJ mol^{-1}) for the Thermal Electrocyclization and Cycloreversion Reactions of 1 and 10, and Atomic Spin Densities at the Corresponding Transition Structures[a]

						spin density[b]						
reaction	$\Delta G_{\mathbf{o}\rightarrow\mathbf{c}}{}^{c}$	$\Delta G^{\ddagger}_{\mathbf{o}\rightarrow\mathbf{c}}{}^{d}$	$\Delta G^{\ddagger}_{\mathbf{c}\rightarrow\mathbf{o}}{}^{e}$	$E_{\mathrm{a},\mathbf{o}\rightarrow\mathbf{c}}{}^{f}$	$E_{\mathrm{a},\mathbf{c}\rightarrow\mathbf{o}}{}^{g}$	C1	C1'	C2	C2'	C6	C6'	$\langle S^2 \rangle^h$
**1o** ↔ **1c**	100.8	205.7	104.9	195.6	107.0	0.21	−0.21	−0.37	0.37	0.18	−0.18	0.85
**10o** ↔ **10c**	90.2	195.5	105.3	184.8	104.8	0.19	−0.19	−0.32	0.32	0.16	−0.16	0.82

[a]For the closed-shell species **1o**/**10o** and **1c**/**10c**, all spin densities are exactly zero. [b]Spin densities are only given for the reactive carbon atoms (these atoms are highlighted in Figure S27). [c]Reaction free energy for electrocyclization. [d]Free-energy barrier for electrocyclization. [e]Free-energy barrier for cycloreversion. [f]Activation energy for electrocyclization. [g]Activation energy for cycloreversion. [h]Expectation value of the spin-squared operator.

S8. NMR spectra of reported compounds

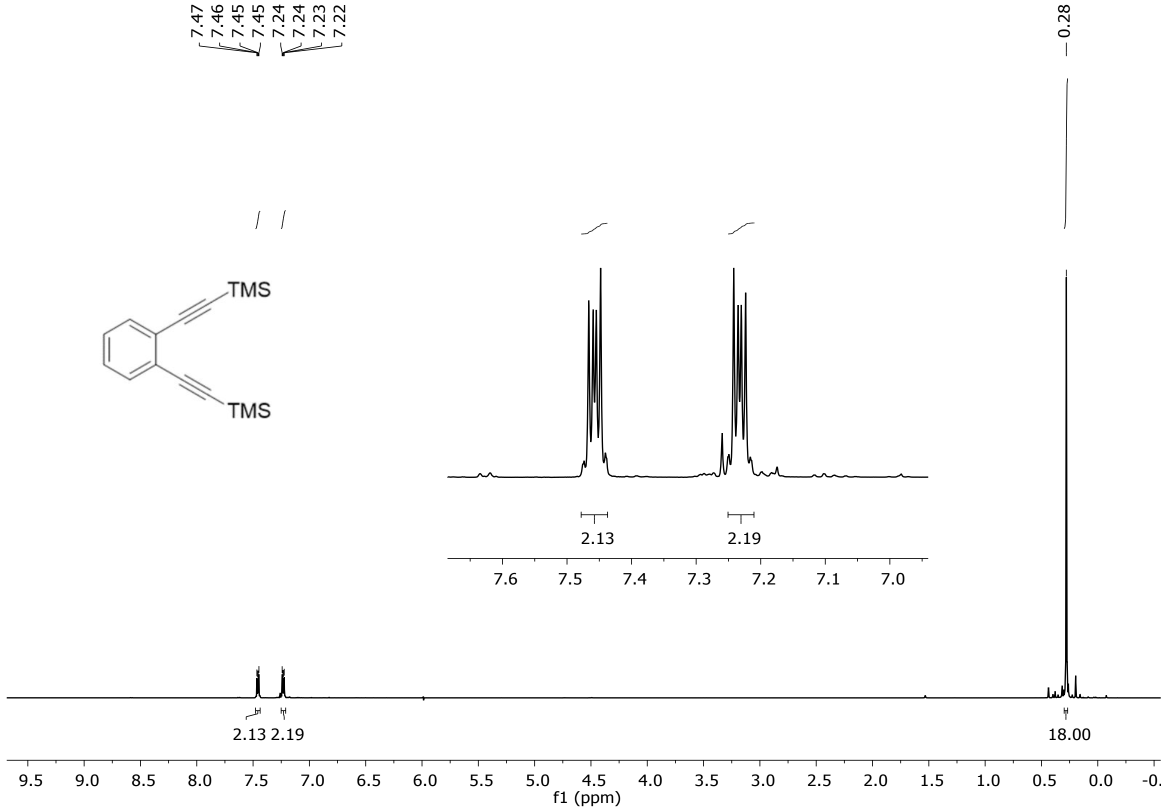

Figure S29. ^{1}H NMR spectrum of **3** in $CDCl_3$ (500 MHz).

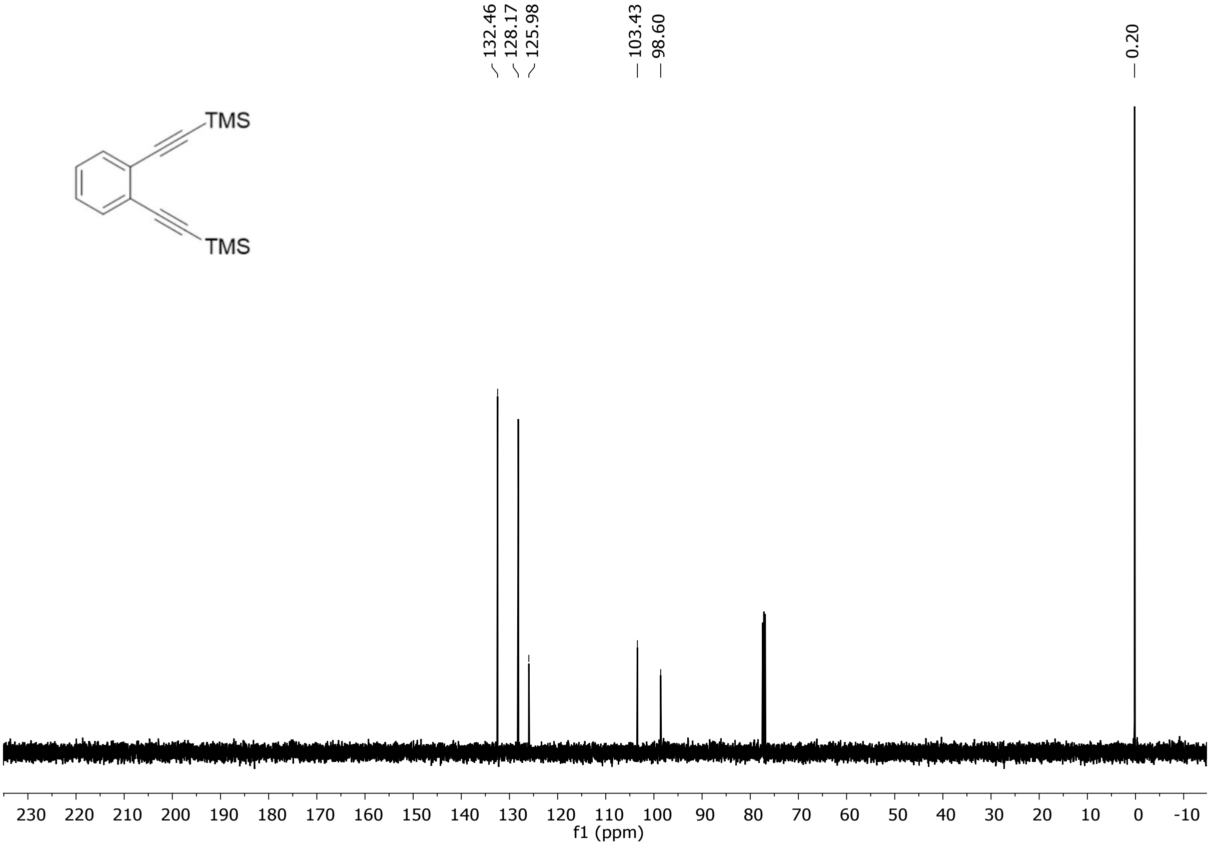

Figure S30. $^{13}C\{^{1}H\}$ NMR spectrum of **3** in $CDCl_3$ (126 MHz).

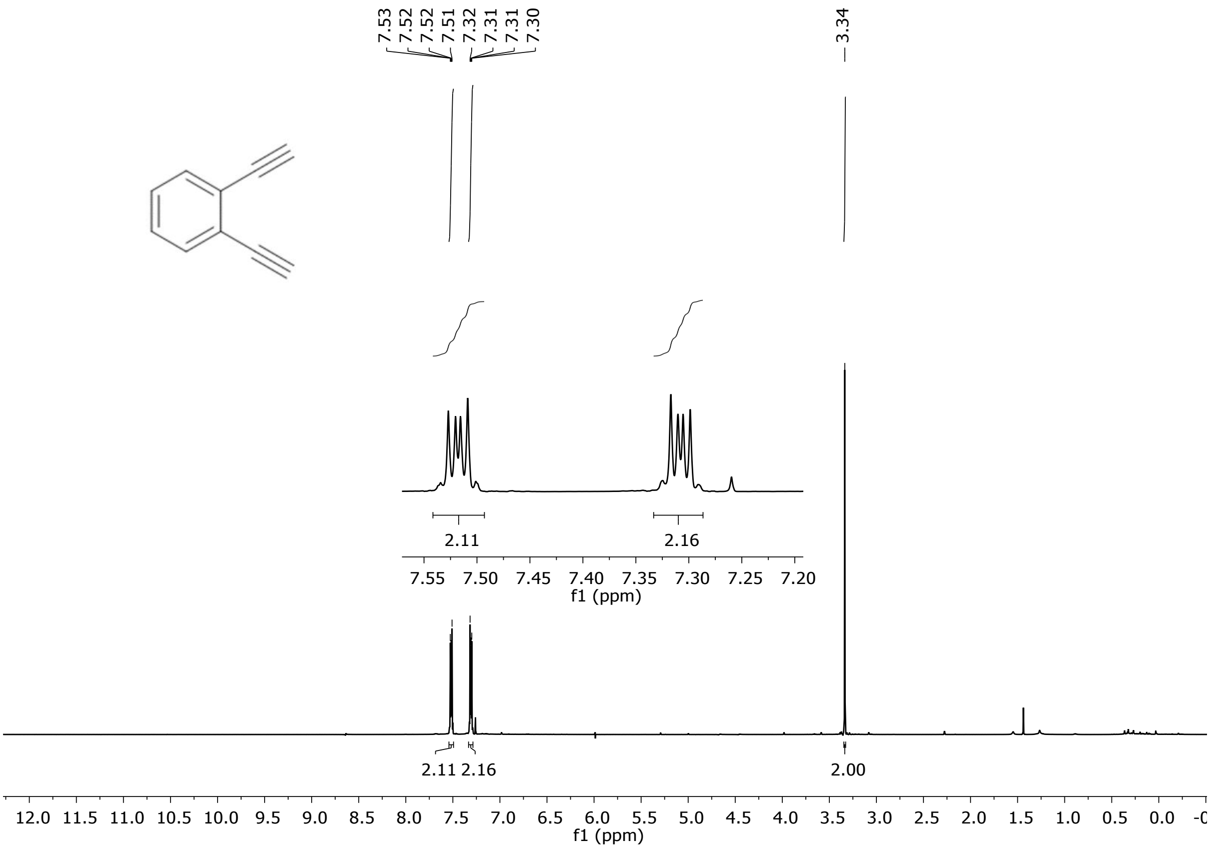

Figure S31. ^{1}H NMR spectrum of **4** in $CDCl_3$ (500 MHz).

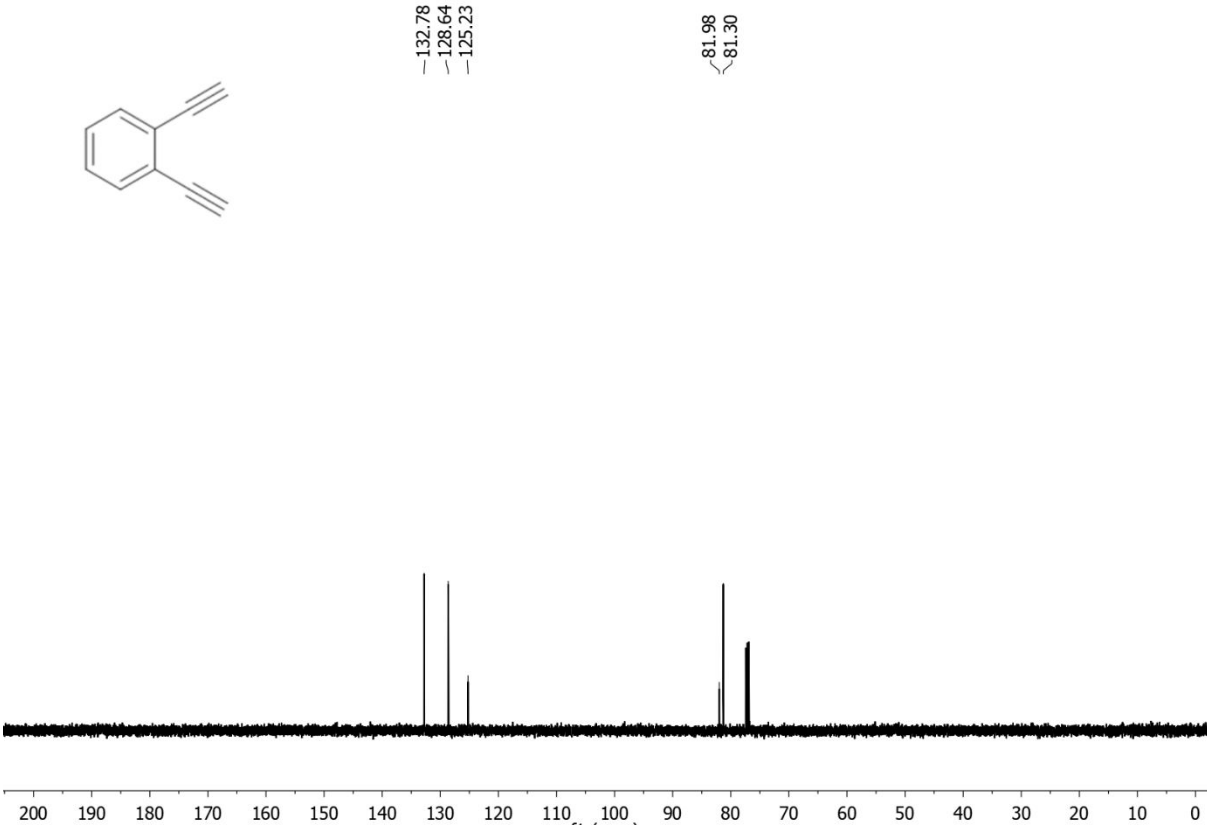

Figure S32. $^{13}C\{^{1}H\}$ NMR spectrum of **4** in $CDCl_3$ (126 MHz).

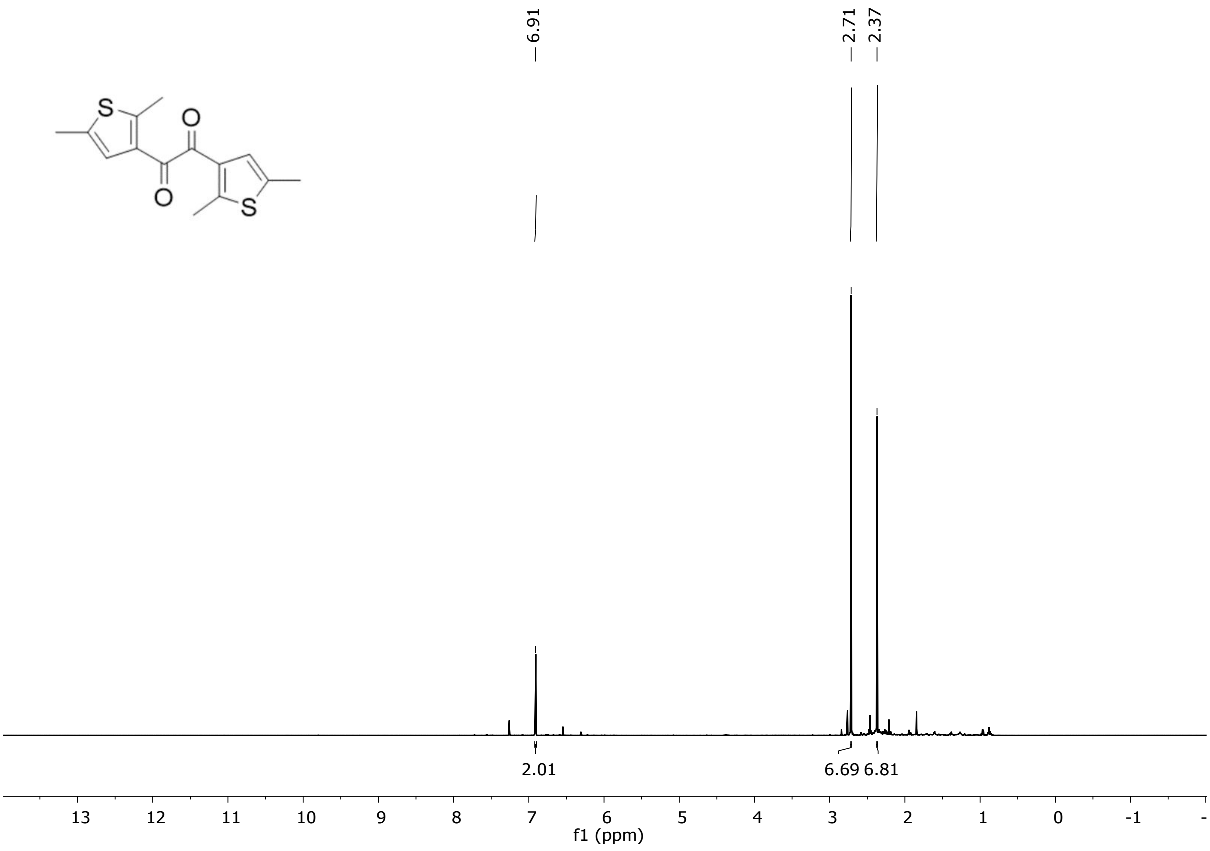

Figure S33. ^{1}H NMR spectrum of **6** in $CDCl_3$ (500 MHz).

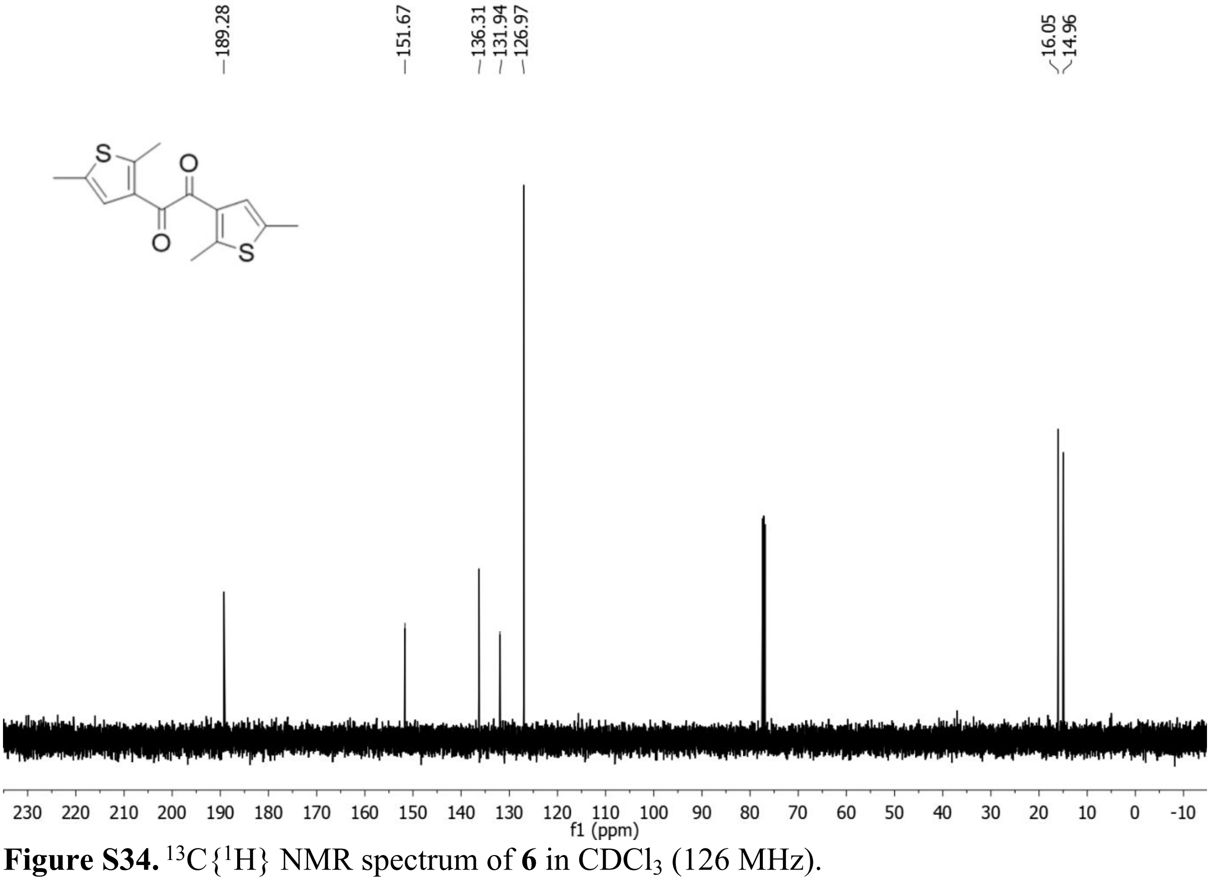

Figure S34. $^{13}C\{^{1}H\}$ NMR spectrum of **6** in $CDCl_3$ (126 MHz).

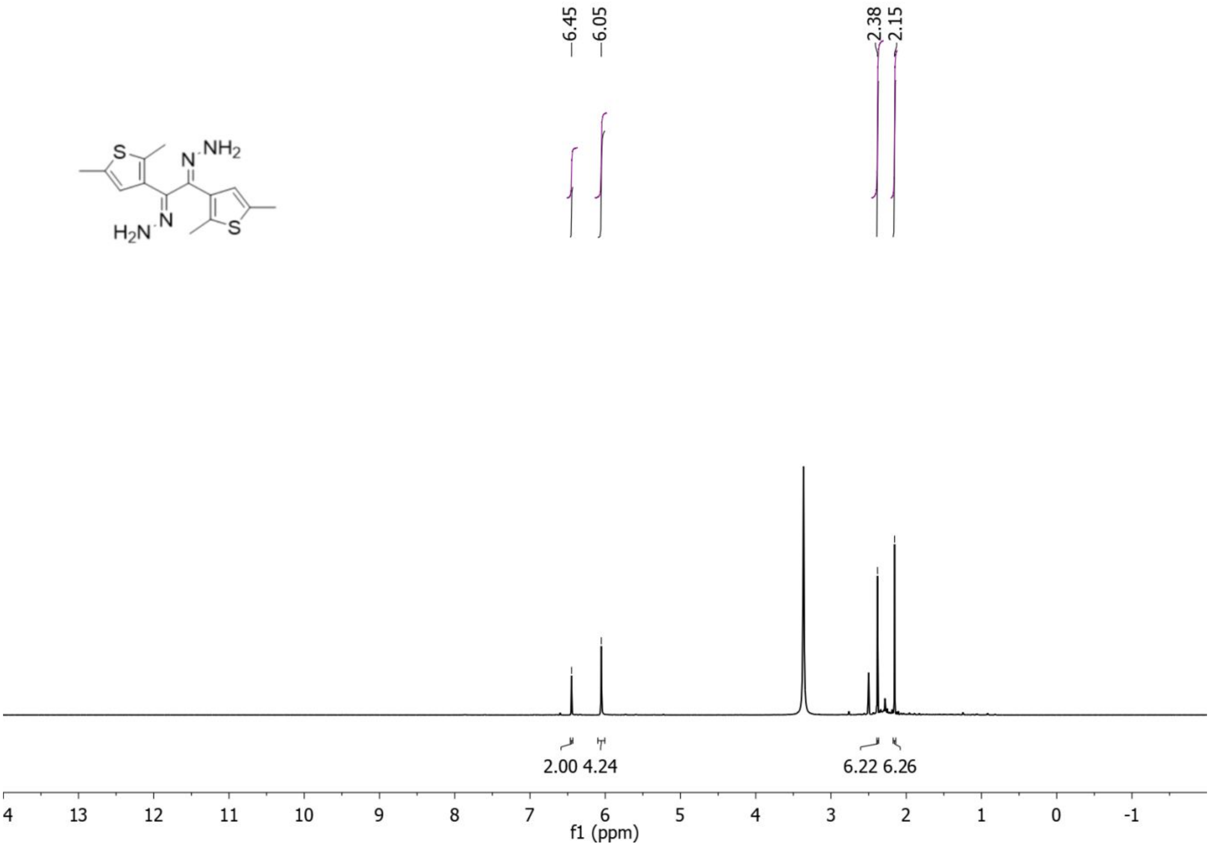

Figure S35. ^{1}H NMR spectrum of **7** in DMSO-d_6 (500 MHz).

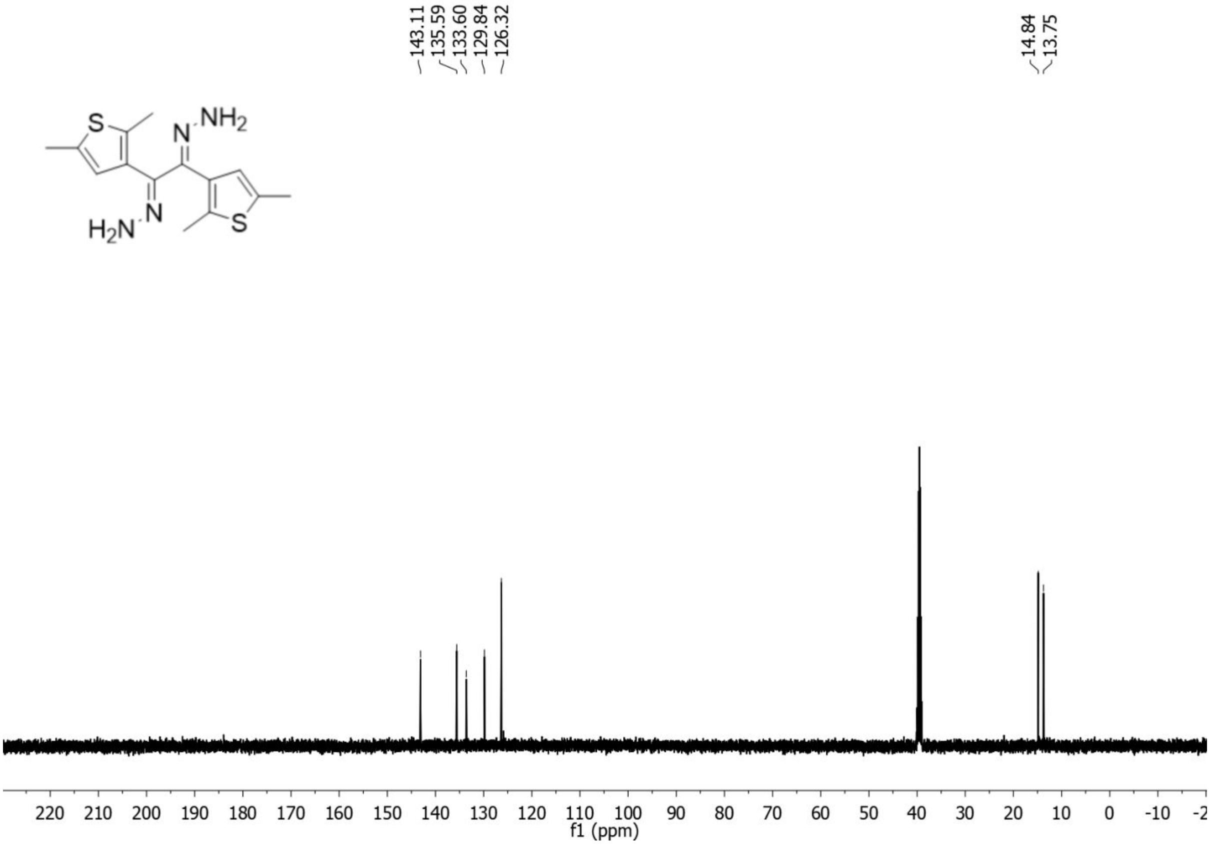

Figure S36. ^{13}C{^{1}H} NMR spectrum of **7** in DMSO-d_6 (126 MHz).

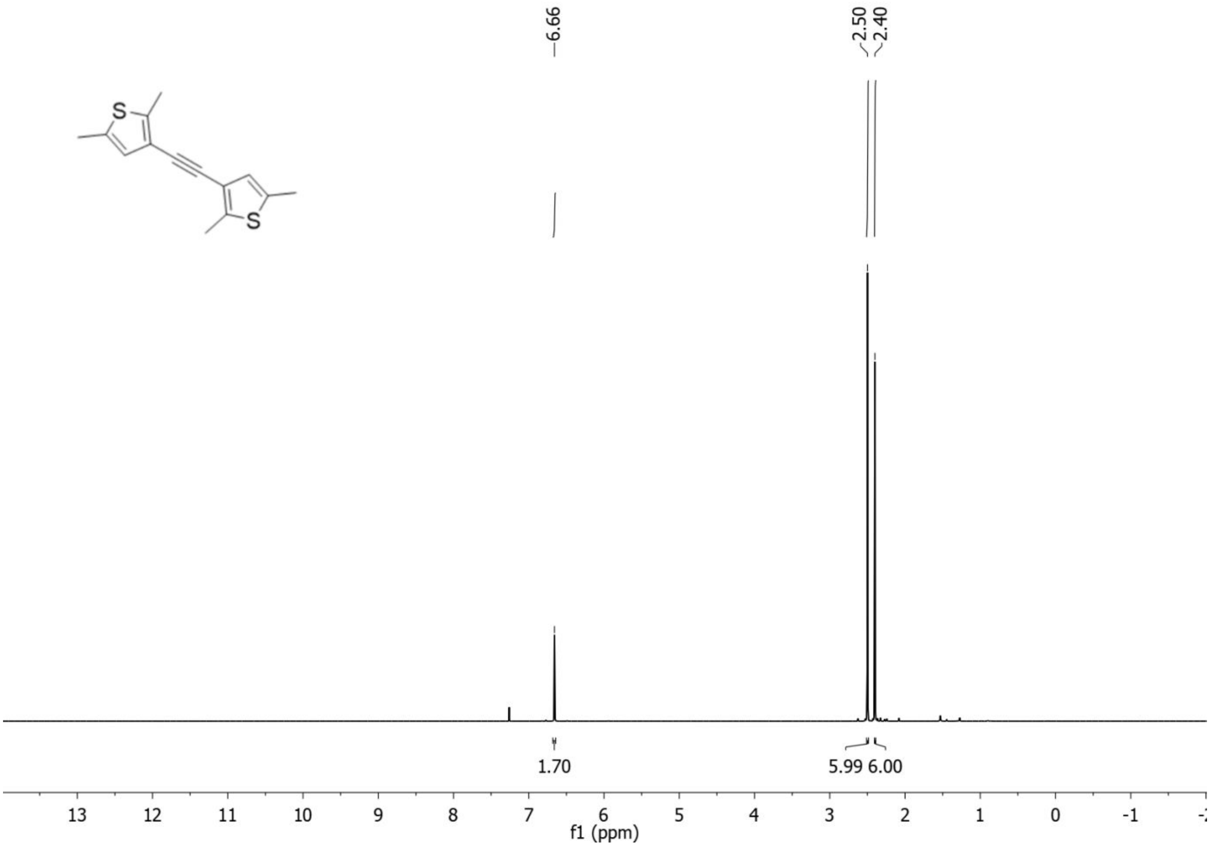

Figure S37. ^{1}H NMR spectrum of **8** in $CDCl_3$ (500 MHz).

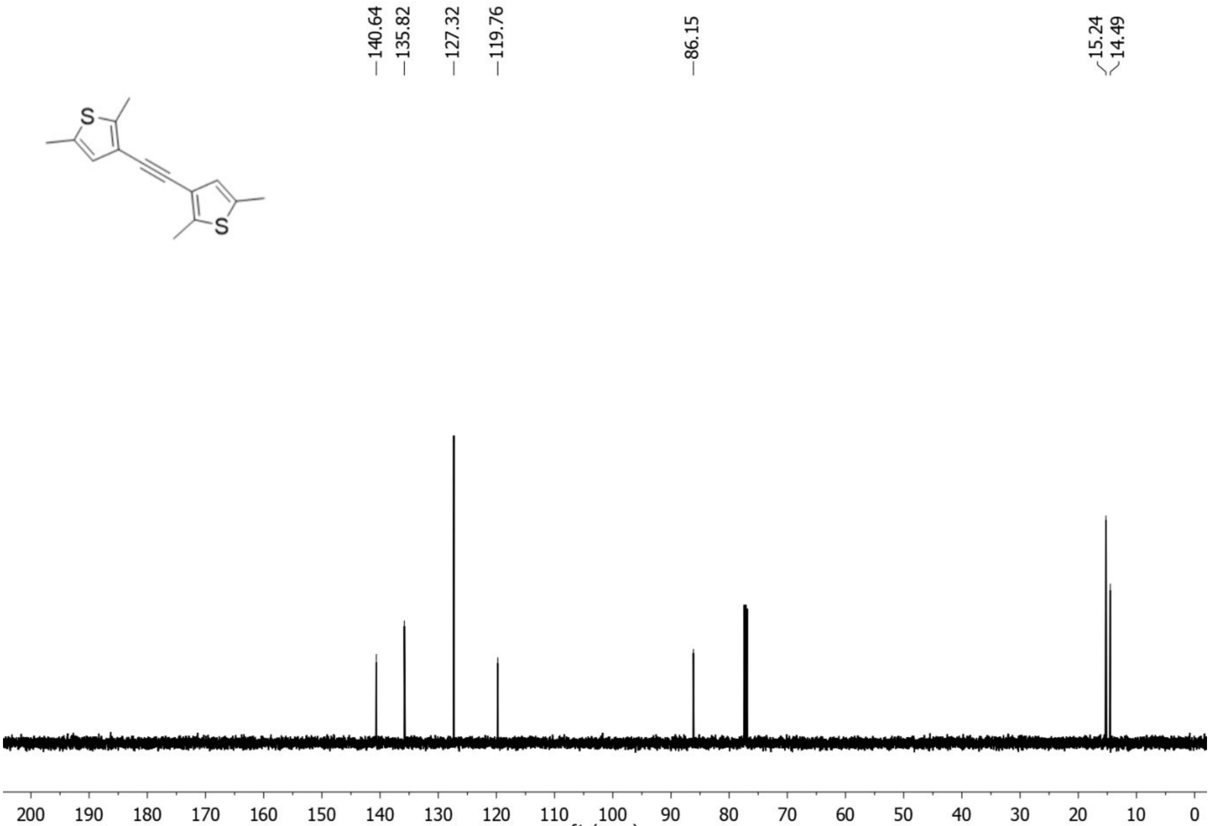

Figure S38. $^{13}C\{^{1}H\}$ NMR spectrum of **8** in $CDCl_3$ (126 MHz).

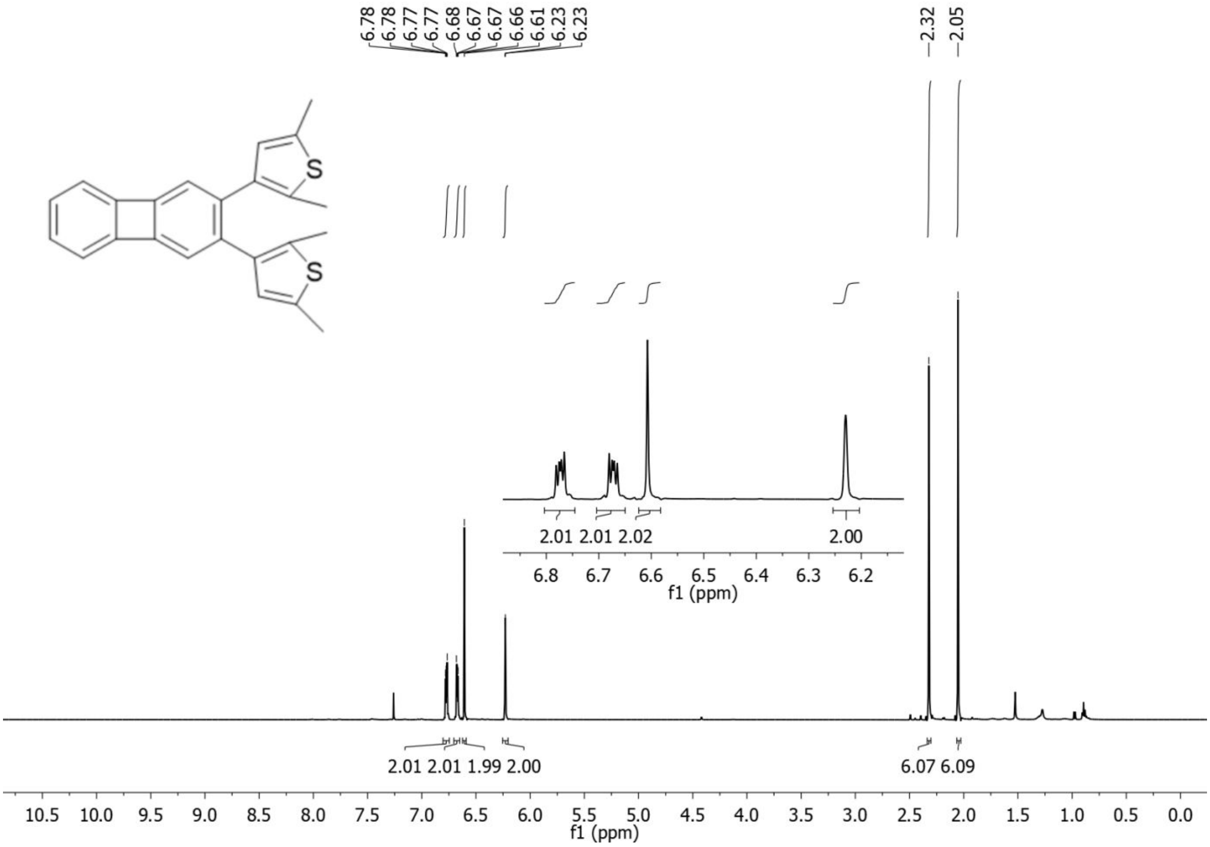

Figure S39. ^{1}H NMR spectrum of **1** in $CDCl_3$ (500 MHz).

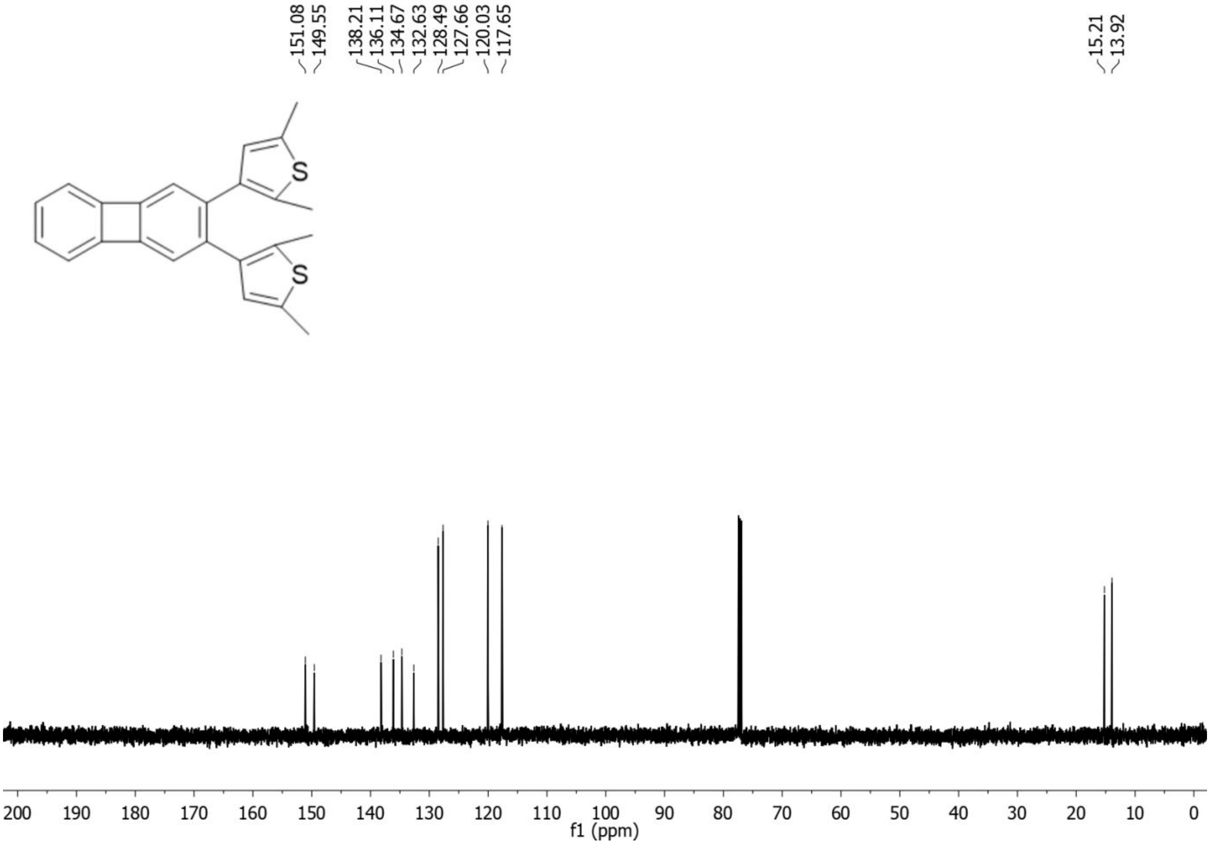

Figure S40. $^{13}C\{^{1}H\}$ NMR spectrum of **1** in $CDCl_3$ (126 MHz).

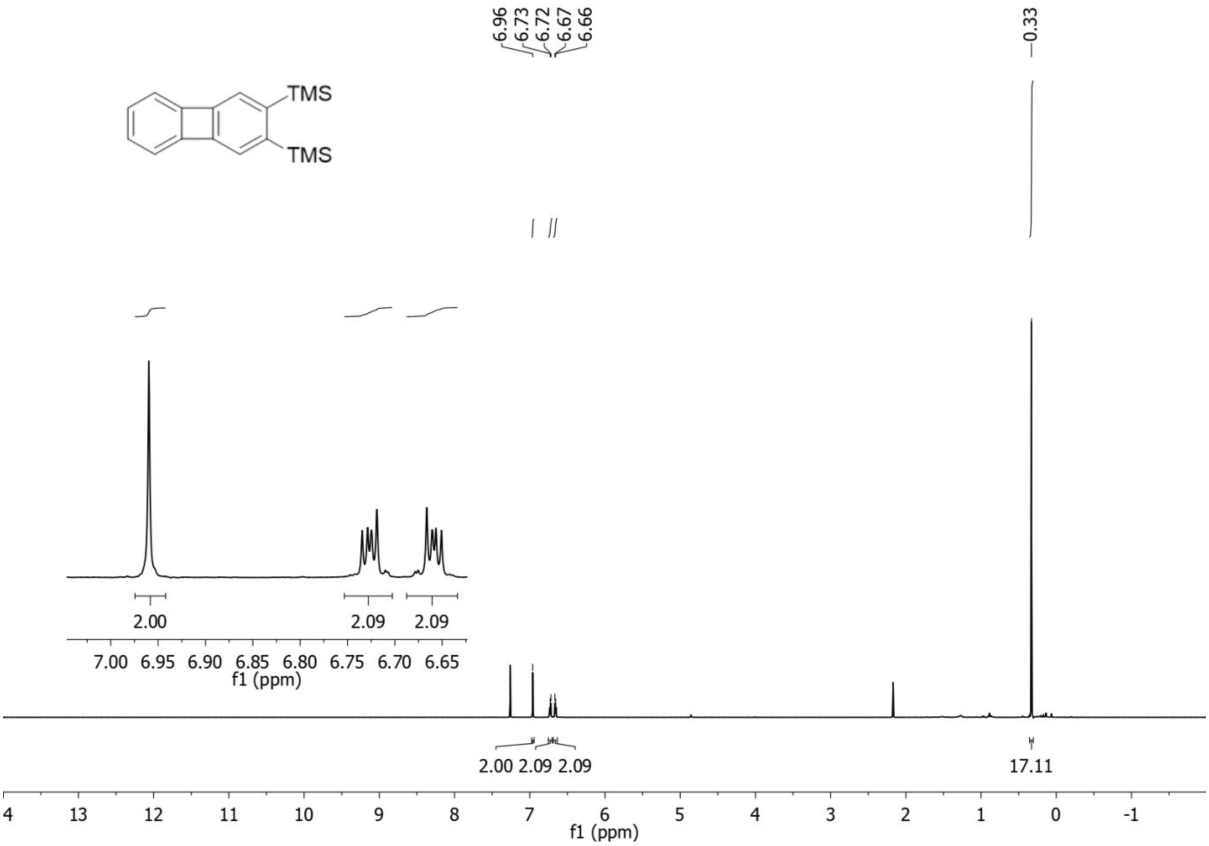

Figure S41. ^{1}H NMR spectrum of **12** in $CDCl_3$ (500 MHz).

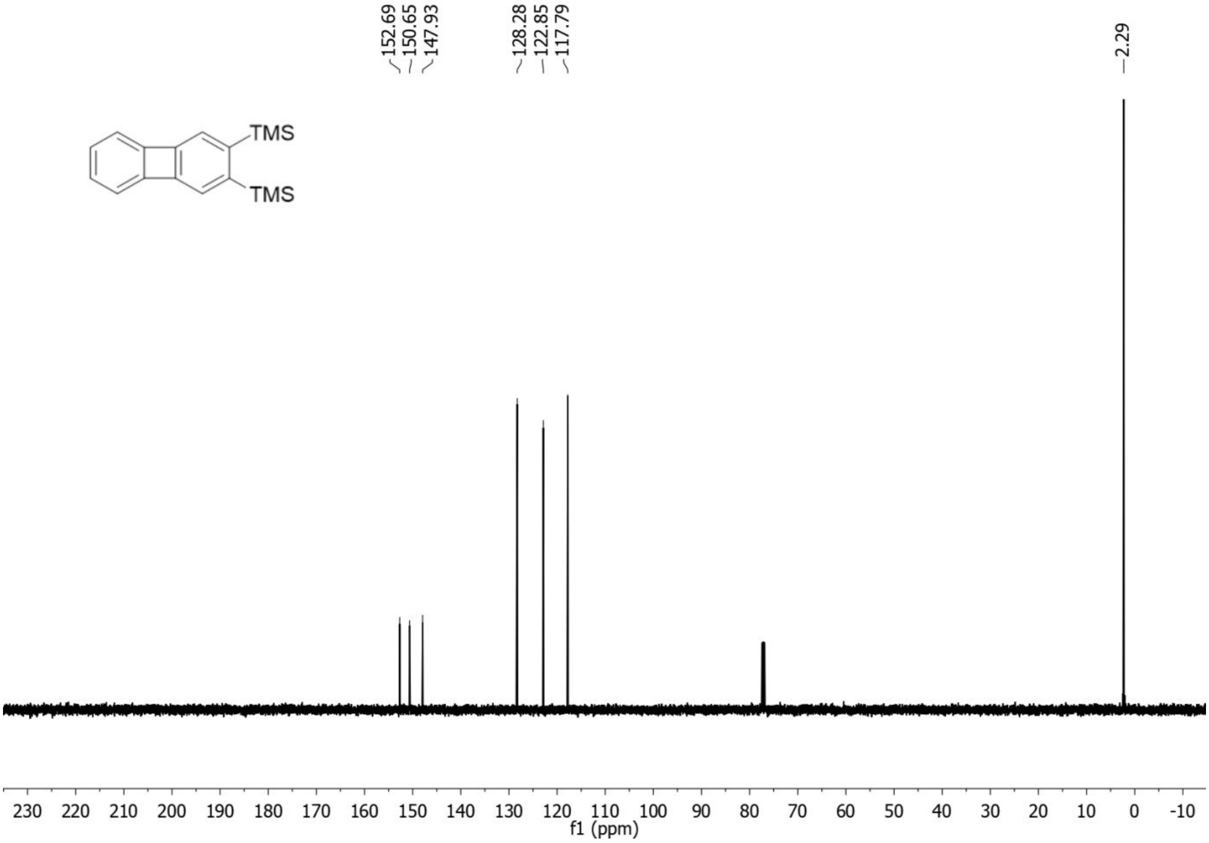

Figure S42. $^{13}C\{^{1}H\}$ NMR spectrum of compound **12** in $CDCl_3$ (126 MHz).

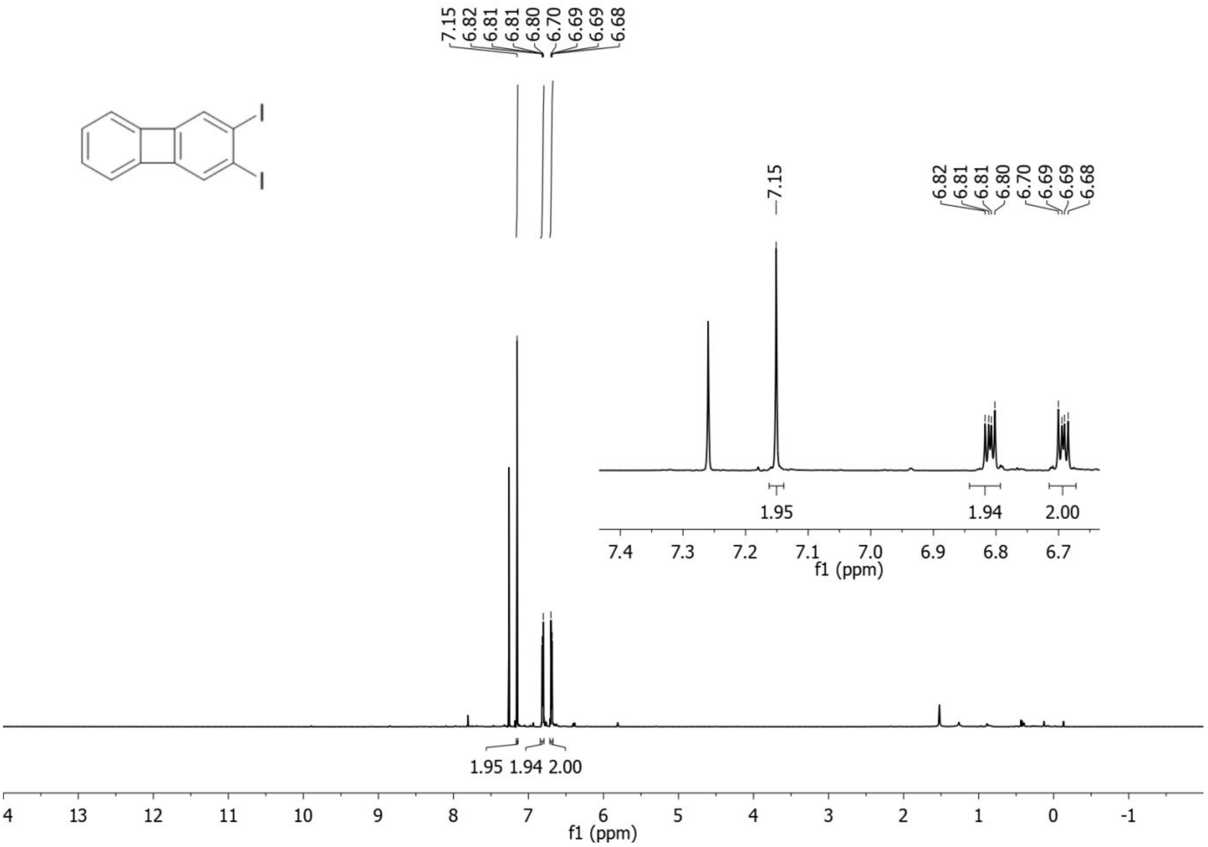

Figure S43. ^{1}H NMR spectrum of **13** in $CDCl_3$ (500 MHz).

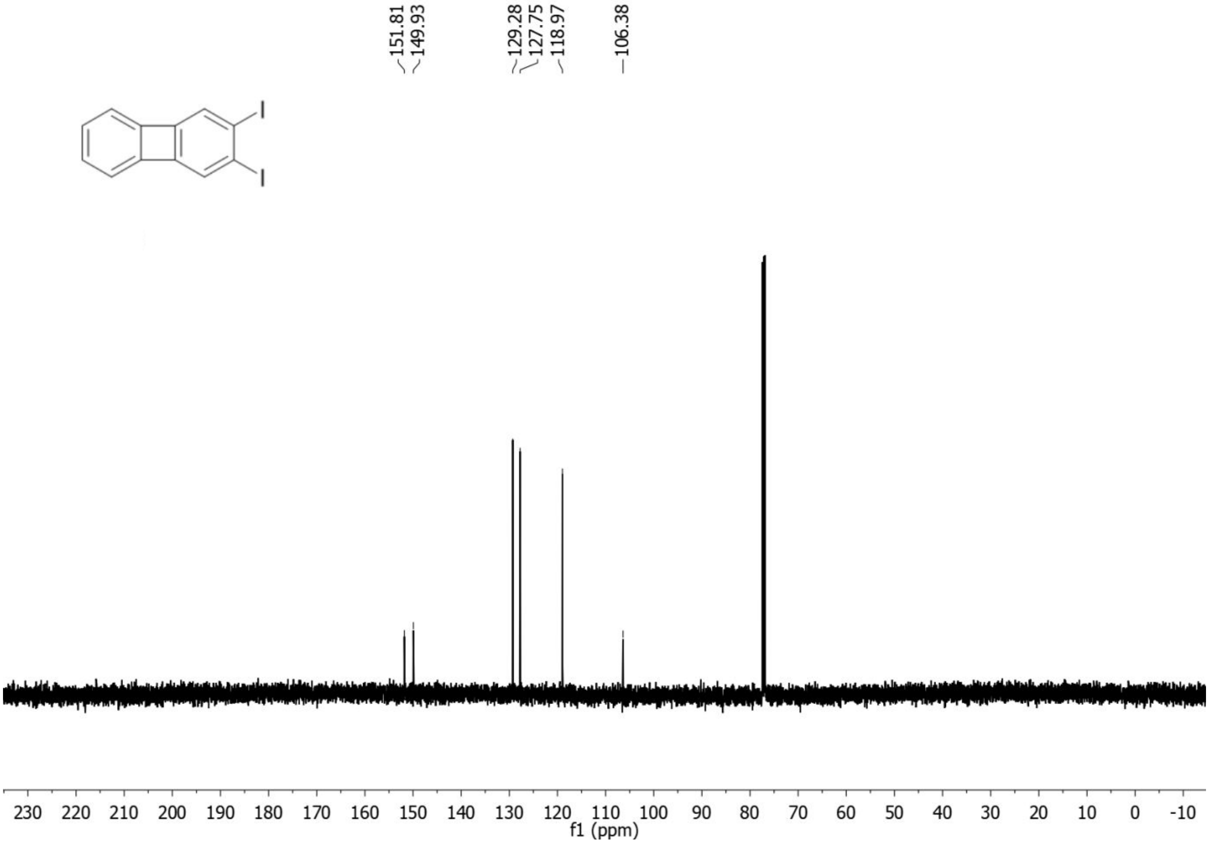

Figure S44. $^{13}C\{^{1}H\}$ NMR spectrum of compound **13** in $CDCl_3$ (126 MHz).

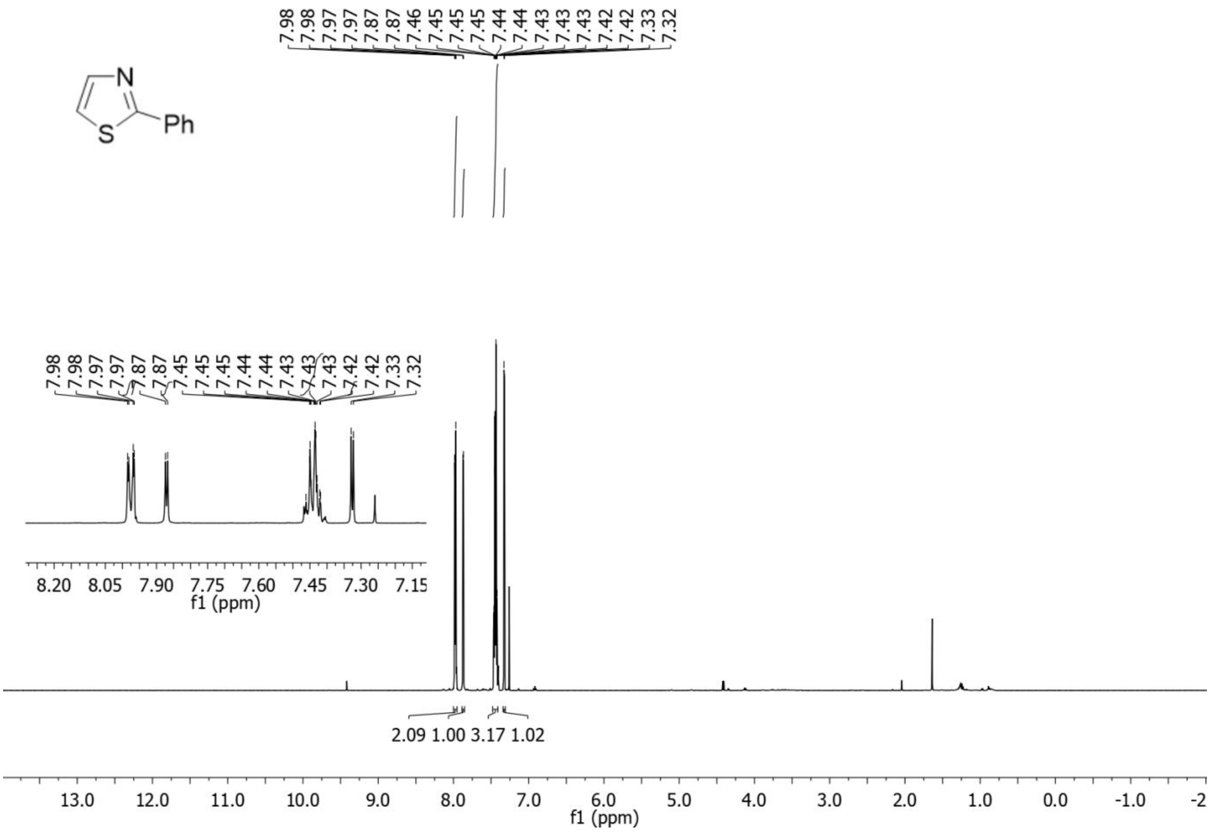

Figure S45. ^{1}H NMR spectrum of **15** in $CDCl_3$ (500 MHz).

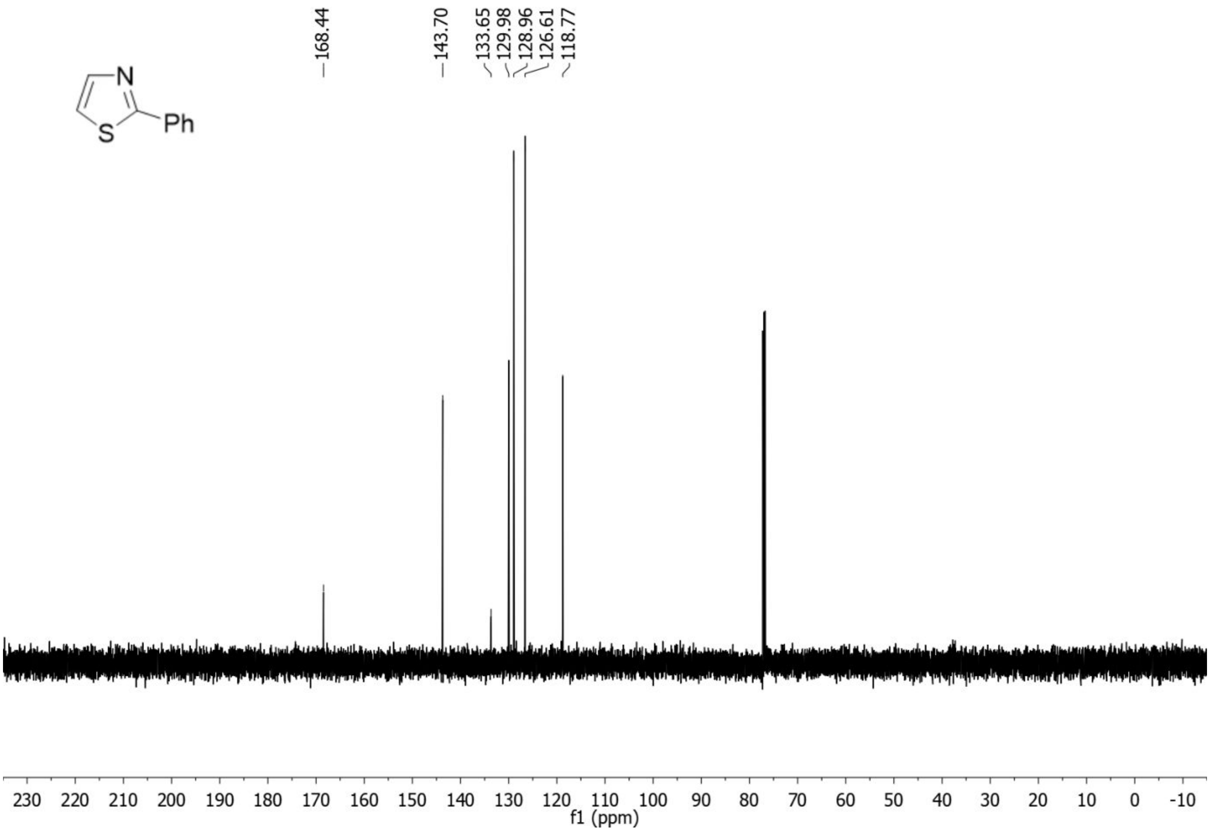

Figure S46. $^{13}C\{^{1}H\}$ NMR spectrum of **15** in $CDCl_3$ (126 MHz).

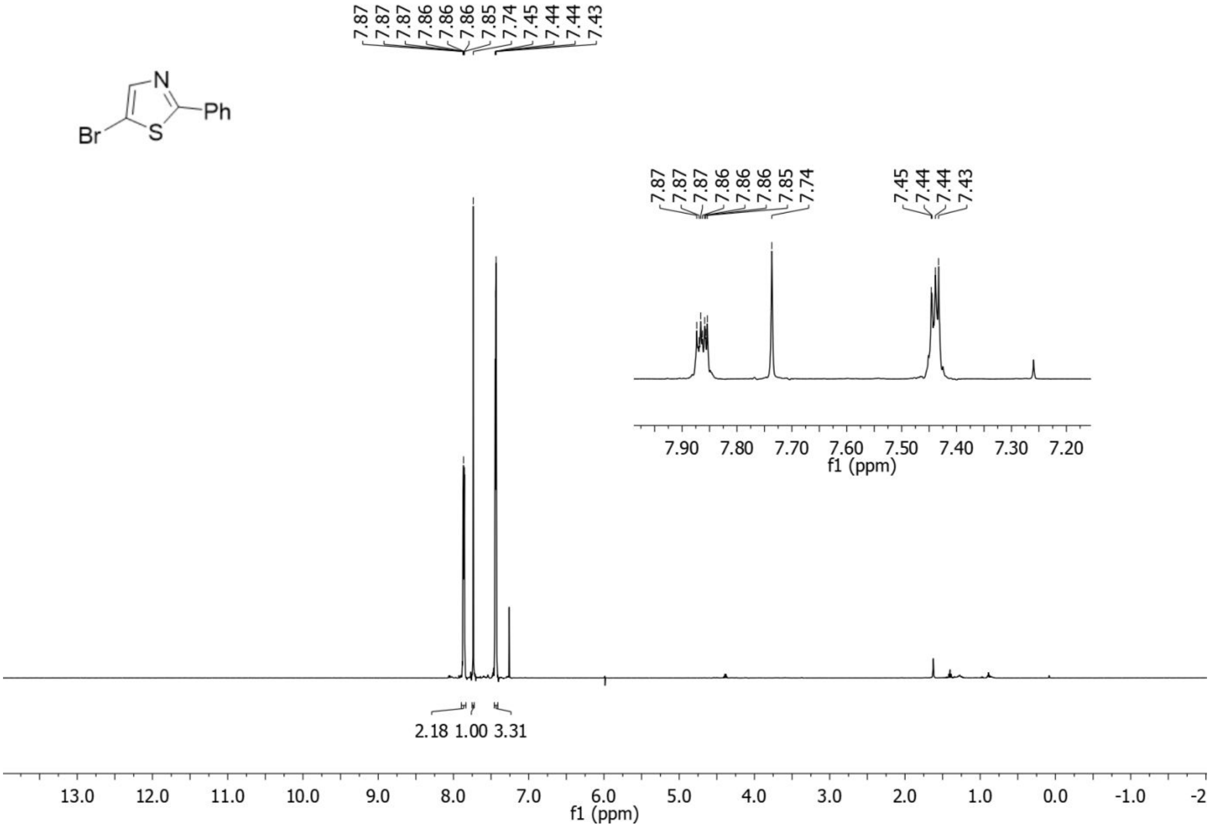

Figure S47. ^{1}H NMR spectrum of **16** in $CDCl_3$ (500 MHz).

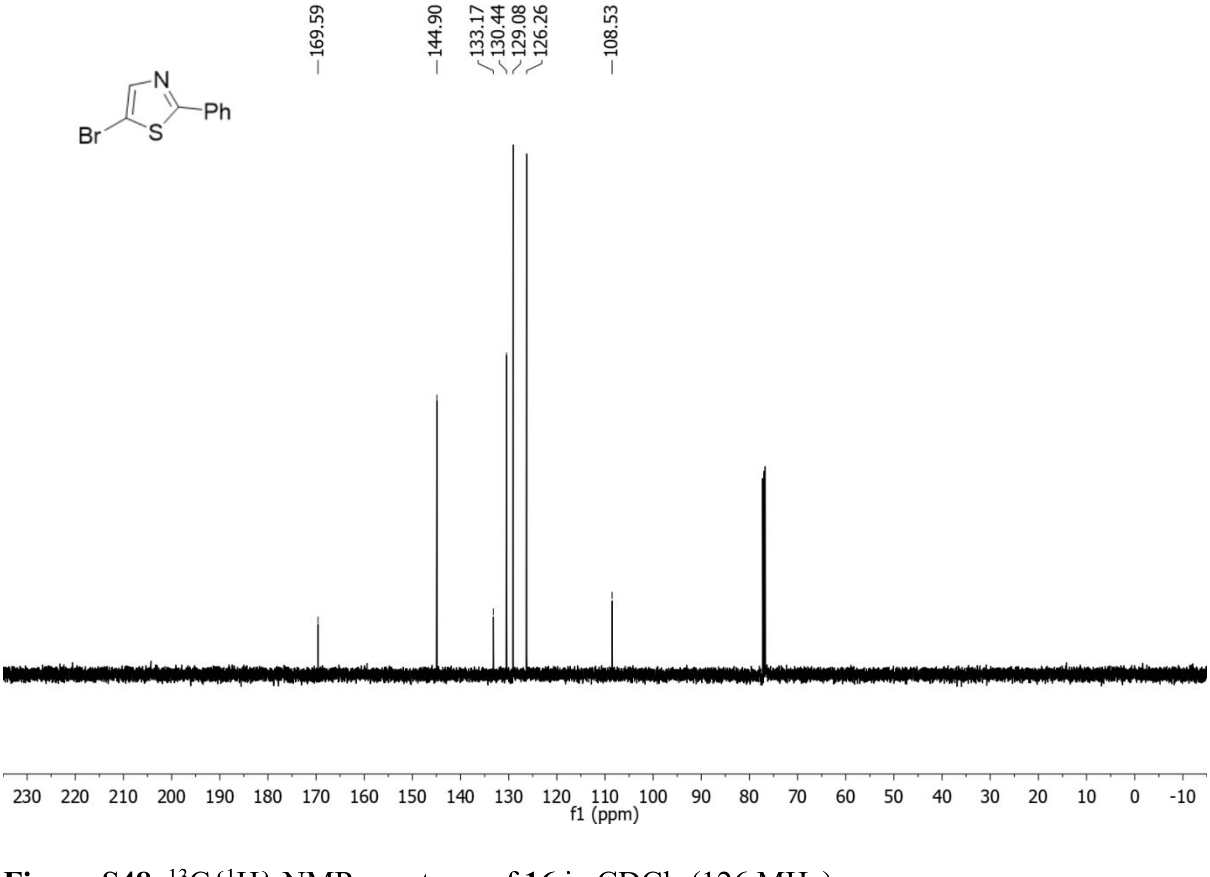

Figure S48. $^{13}C\{^{1}H\}$ NMR spectrum of **16** in $CDCl_3$ (126 MHz).

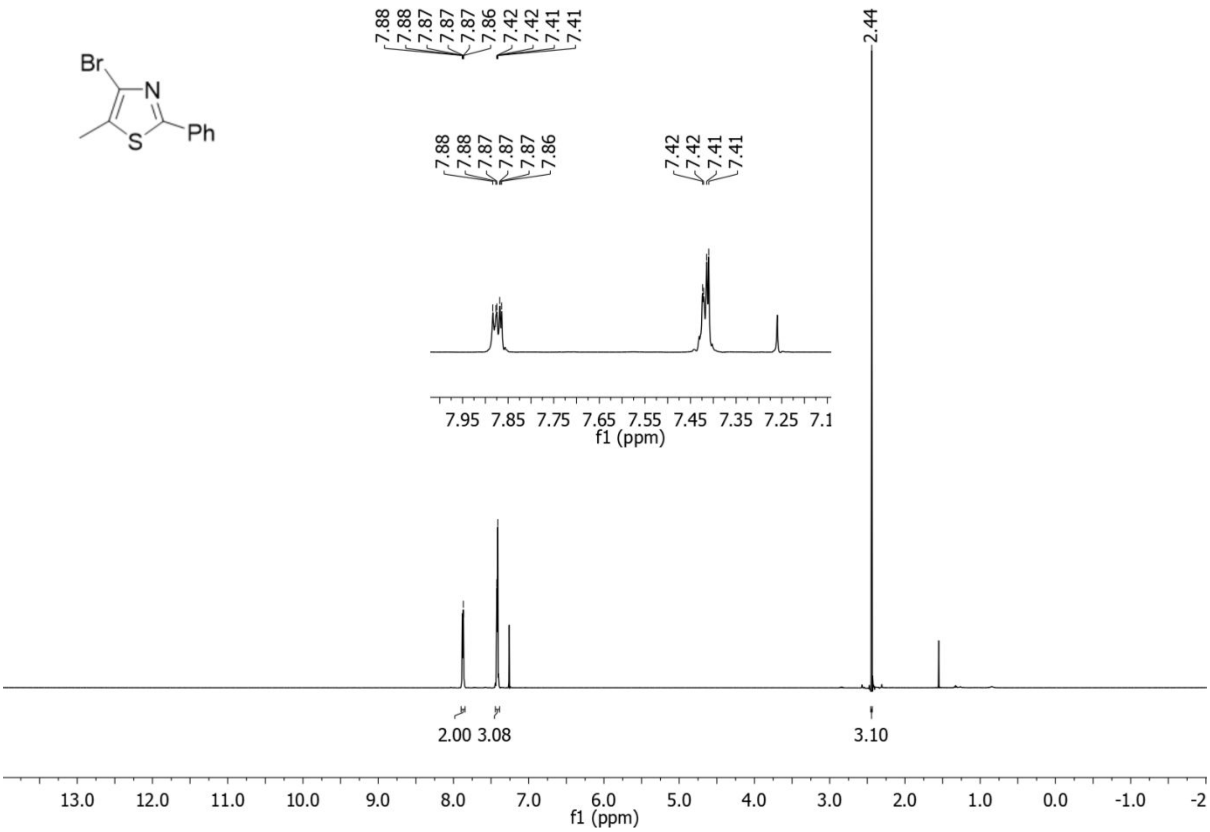

Figure S49. ^{1}H NMR spectrum of **17** in $CDCl_3$ (500 MHz).

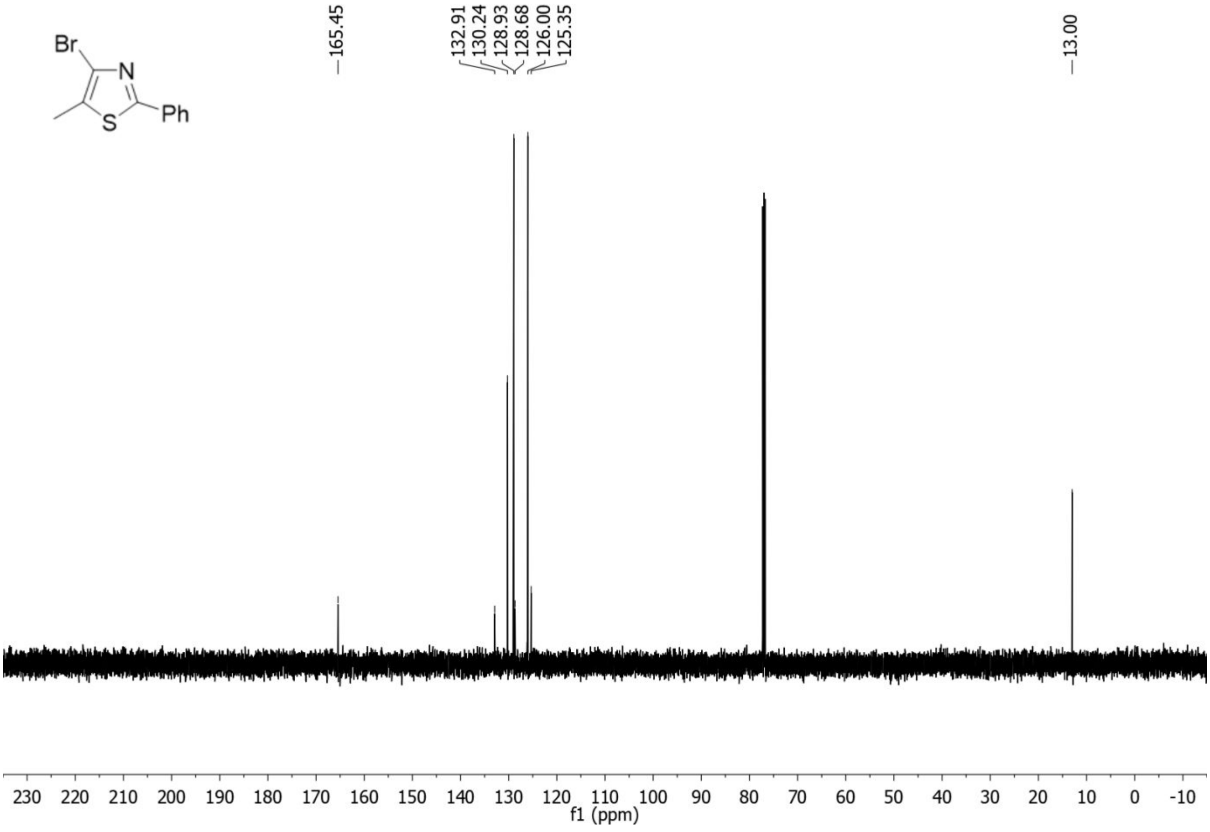

Figure 50. $^{13}C\{^{1}H\}$ NMR spectrum of **17** in $CDCl_3$ (126 MHz).

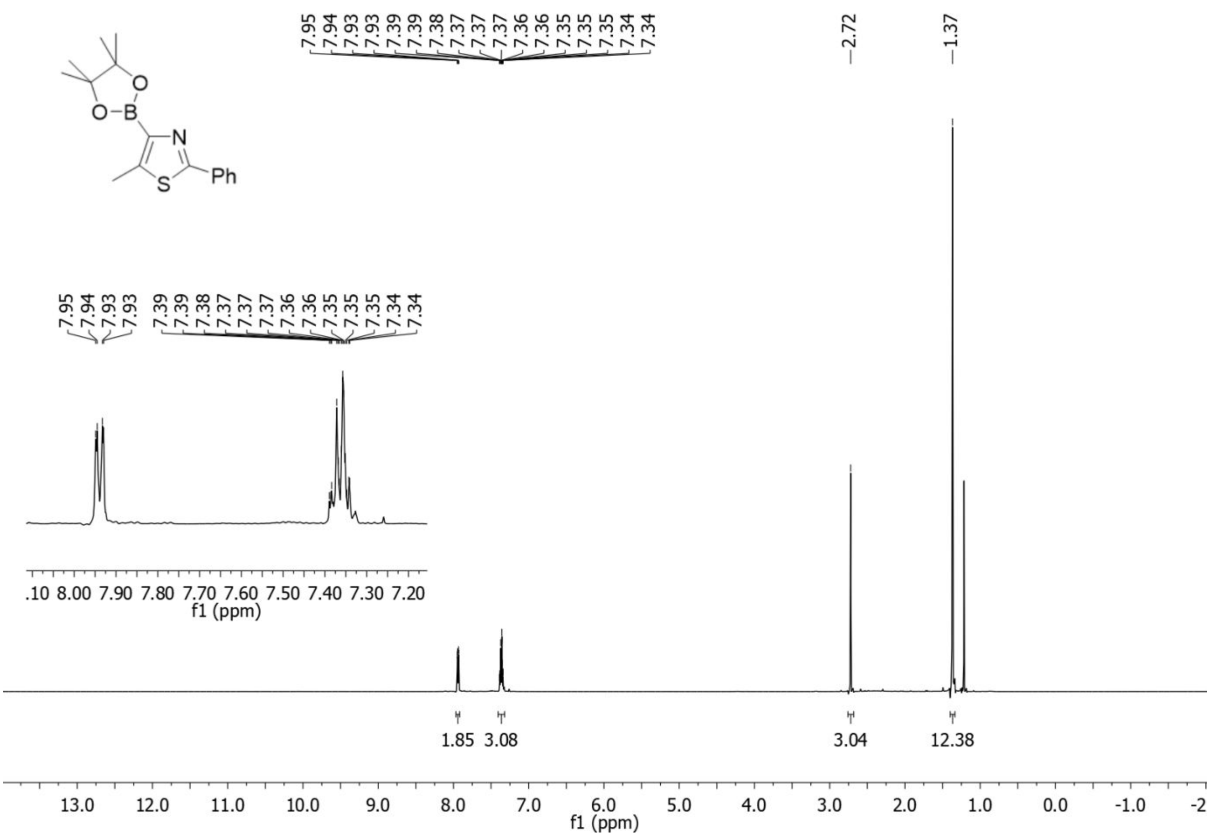

Figure S51. ^{1}H NMR spectrum of **18** in $CDCl_3$ (500 MHz).

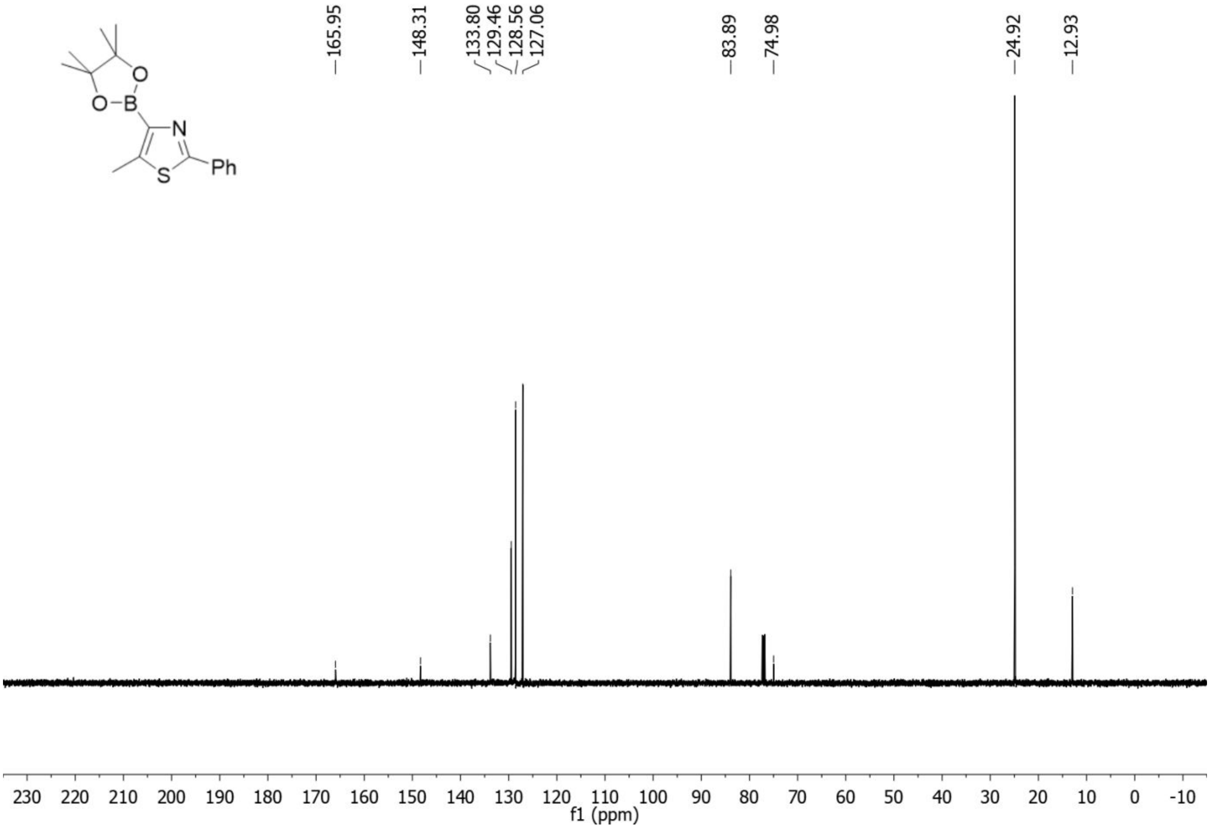

Figure S52. $^{13}C\{^{1}H\}$ NMR spectrum of **18** in $CDCl_3$ (126 MHz).

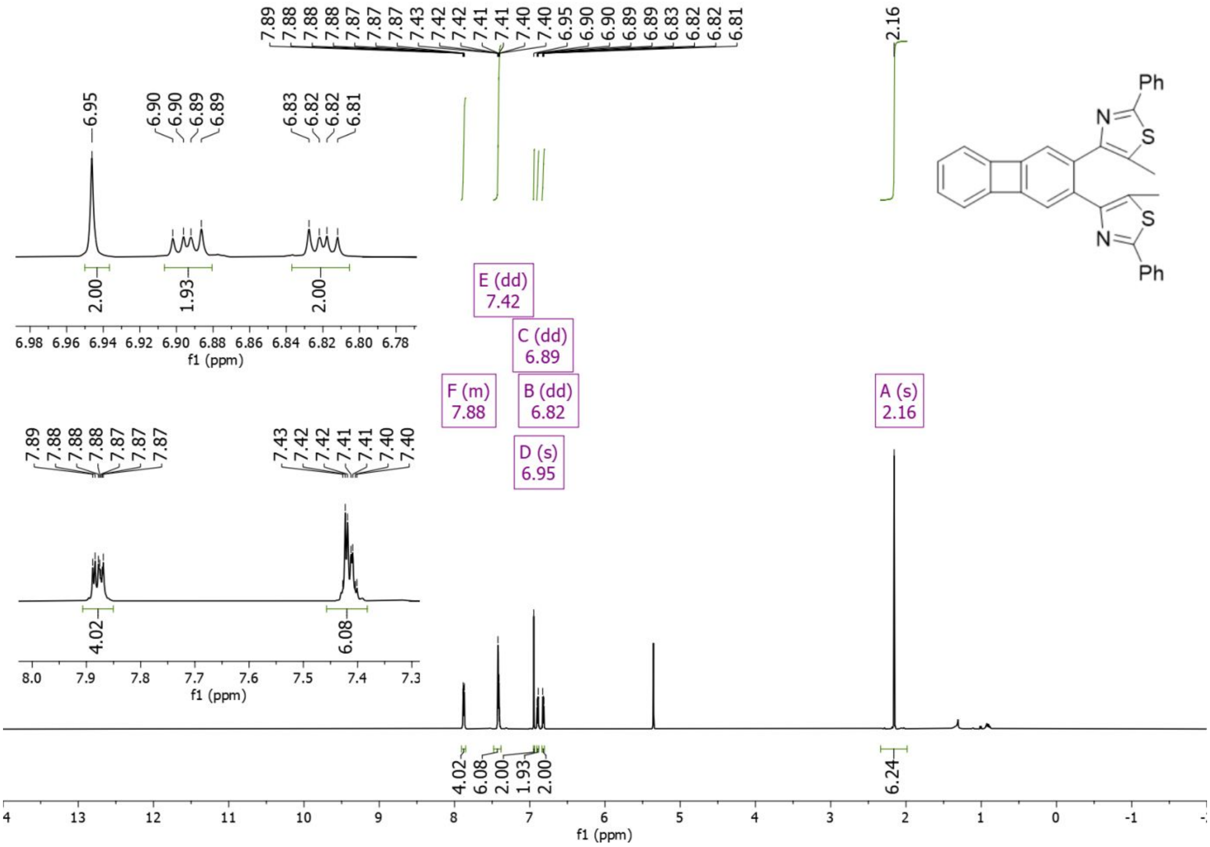

Figure S53. ^{1}H NMR spectrum of **10** in $CDCl_3$ (500 MHz).

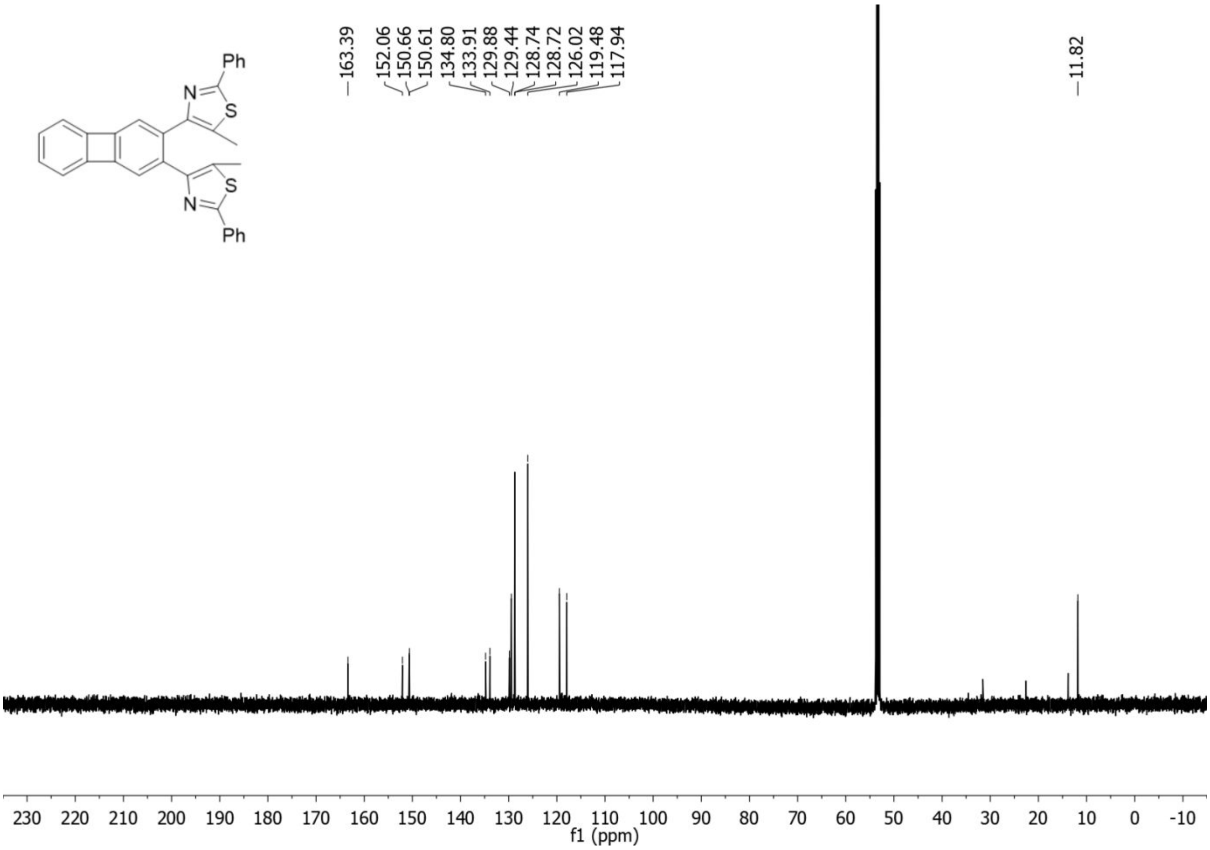

Figure S54. $^{13}C\{^{1}H\}$ NMR spectrum of **10** in $CDCl_3$ (126 MHz).

S9. HRMS of 1 and 10

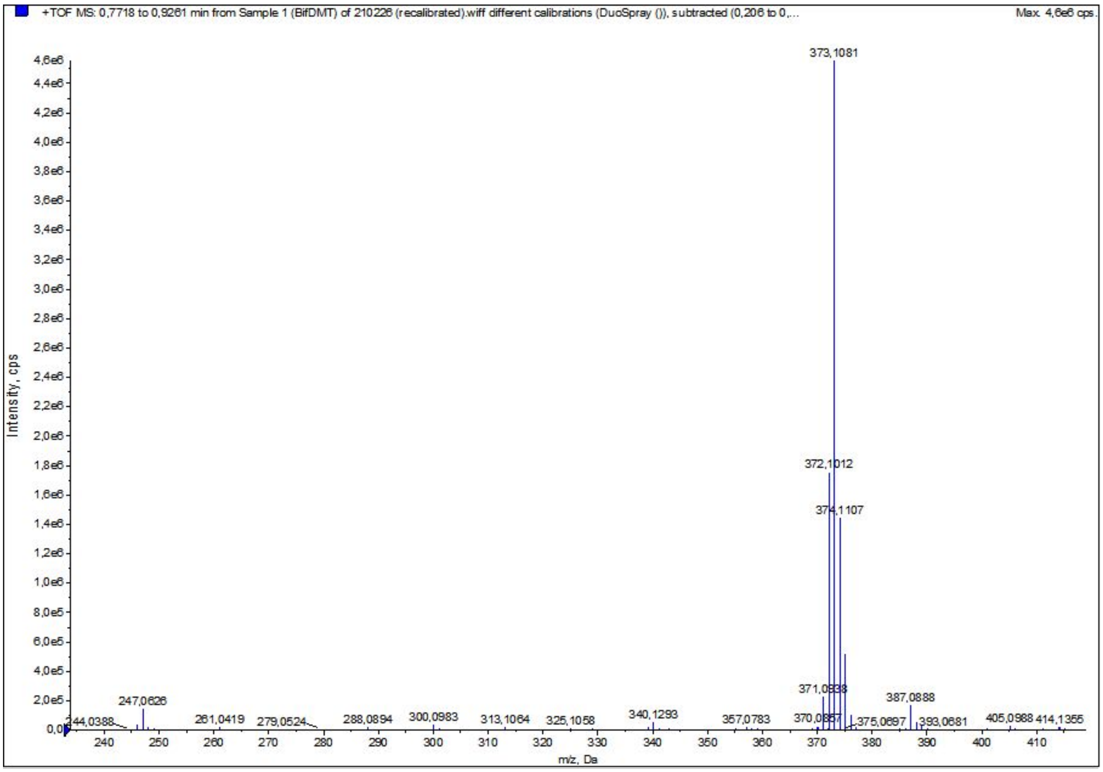

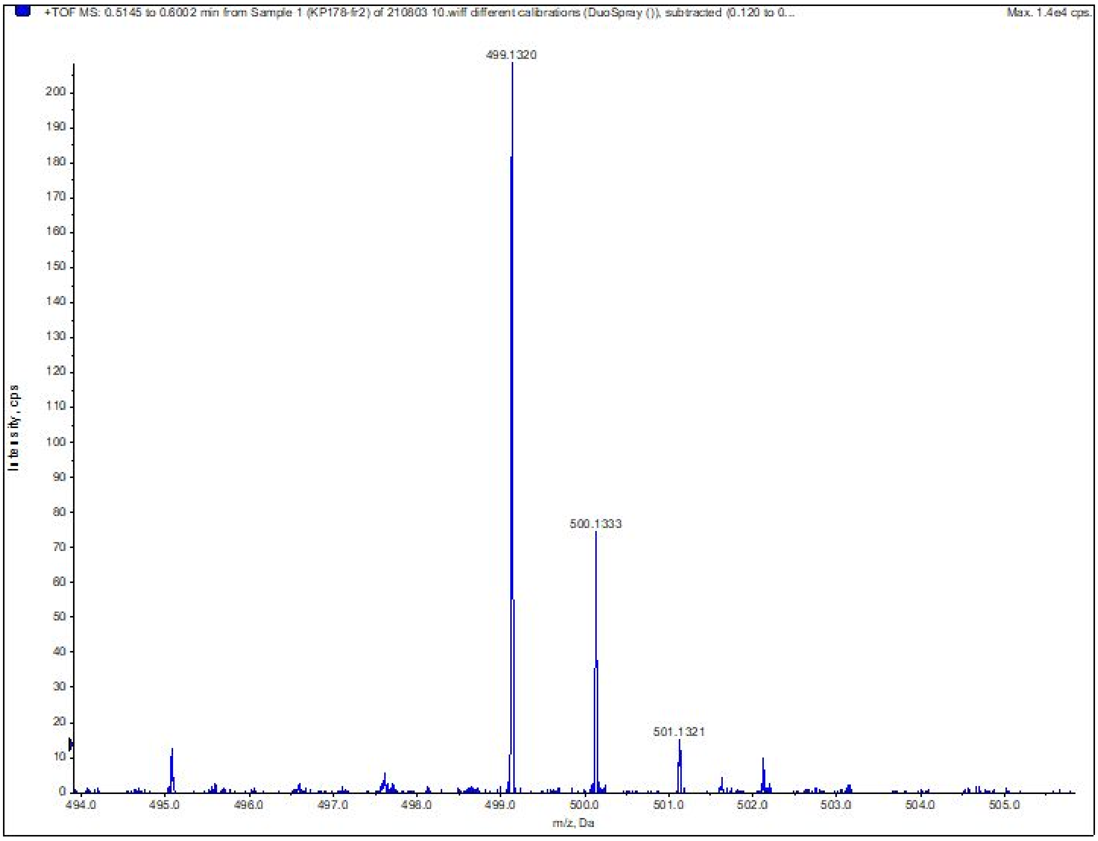

Figure S55. Upper: HRMS spectrum of **1**; lower: HRMS spectrum of **10**.

S10. LED emission spectra

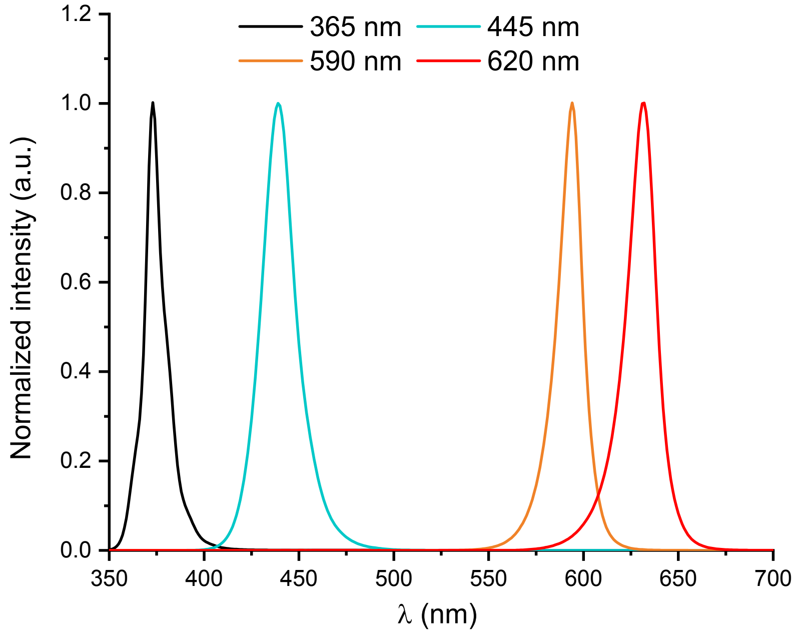

Figure S56. LED emission spectra measured on a Jasco FP 8300 spectrofluorometer.

S11. References

(1) (a) Irie, M.; Lifka, T.; Uchida, K.; Kobatake, S.; Shindo, Y. Fatigue Resistant Properties of Photochromic Dithienylethenes: By-Product Formation. *Chem. Commun.* **1999**, 747–750; (b) Herder, M.; Schmidt, B. M.; Grubert, L.; Pätzel, M.; Schwarz, J.; Hecht, S. Improving the Fatigue Resistance of Diarylethene Switches. *J. Am. Chem. Soc.* **2015**, *137*, 2738–2747; (c) Pariani, G.; Quintavalla, M.; Colella, L.; Oggioni, L.; Castagna, R.; Ortica, F.; Bertarelli, C.; Bianco, A. New Insight into the Fatigue Resistance of Photochromic 1,2-Diarylethenes. *J. Phys. Chem. C* **2017**, *121*, 23592–23598; (d) Lvov, A. G.; Mörtel, M.; Heinemann, F. W.; Khusniyarov, M. M. One-Way Photoisomerization of Ligands for Permanent Switching of Metal Complexes. *J. Mater. Chem. C.* **2021**, *9*, 4757–4763.

(2) Marenich, A. V.; Cramer, C. J.; Truhlar, D. G. Universal Solvation Model Based on Solute Electron Density and on a Continuum Model of the Solvent Defined by the Bulk Dielectric Constant and Atomic Surface Tensions. *J. Phys. Chem. B* **2009**, *113*, 6378–6396.

(3) Sun, C.-L.; Wang, C.; Boulatov, R. Applications of Photoswitches in the Storage of Solar Energy. *ChemPhotoChem* **2019**, *3*, 268–283.

(4) (a) Schleyer, P. v. R.; Maerker, C.; Dransfeld, A.; Jiao, H.; Van Eikema Hommes, N. J. R. Nucleus-Independent Chemical Shifts: A Simple and Efficient Aromaticity Probe. *J. Am. Chem. Soc.* **1996**, *118*, 6317–6318; (b) Fallah-Bagher-Shaidaei, H.; Wannere, C. S.; Corminboeuf, C.; Puchta, R.; Schleyer, P. v. R. Which NICS Aromaticity Index for Planar π Rings is Best? *Org. Lett.* **2006**, *8*, 863–866.

(5) (a) Stanger, A. Nucleus-Independent Chemical Shifts (NICS): Distance Dependence and Revised Criteria for Aromaticity and Antiaromaticity. *J. Org. Chem.* **2006**, *71*, 883–893; (b) Gershoni-Poranne, R.; Stanger, A. The NICS-XY-Scan: Identification of Local and Global Ring Currents in Multi-Ring Systems. *Chem. Eur. J.* **2014**, *20*, 5673–5688.

(6) Stanger, A. Reexamination of NICSπ,zz: Height Dependence, Off-Center Values, and Integration. *J. Phys. Chem. A* **2019**, *123*, 3922–3927.

(7) Gaussian 16, Revision B.01, Frisch, M. J.; Trucks, G. W.; Schlegel, H. B.; Scuseria, G. E.; Robb, M. A.; Cheeseman, J. R.; Scalmani, G.; Barone, V.; Petersson, G. A.; Nakatsuji, H.; Li, X.; Caricato, M.; Marenich, A. V.; Bloino, J.; Janesko, B. G.; Gomperts, R.; Mennucci, B.; Hratchian, H. P.; Ortiz, J. V.; Izmaylov, A. F.;

Sonnenberg, J. L.; Williams-Young, D.; Ding, F.; Lipparini, F.; Egidi, F.; Goings, J.; Peng, B.; Petrone, A.; Henderson, T.; Ranasinghe, D.; Zakrzewski, V. G.; Gao, J.; Rega, N.; Zheng, G.; Liang, W.; Hada, M.; Ehara, M.; Toyota, K.; Fukuda, R.; Hasegawa, J.; Ishida, M.; Nakajima, T.; Honda, Y.; Kitao, O.; Nakai, H.; Vreven, T.; Throssell, K.; Montgomery, J. A., Jr.; Peralta, J. E.; Ogliaro, F.; Bearpark, M. J.; Heyd, J. J.; Brothers, E. N.; Kudin, K. N.; Staroverov, V. N.; Keith, T. A.; Kobayashi, R.; Normand, J.; Raghavachari, K.; Rendell, A. P.; Burant, J. C.; Iyengar, S. S.; Tomasi, J.; Cossi, M.; Millam, J. M.; Klene, M.; Adamo, C.; Cammi, R.; Ochterski, J. W.; Martin, R. L.; Morokuma, K.; Farkas, O.; Foresman, J. B.; Fox, D. J. Gaussian, Inc., Wallingford CT, 2016.

(8) (a) Kruszewski, J.; Krygowski, T. M. Definition of Aromaticity Basing on the Harmonic Oscillator Model. *Tetrahedron Lett.* **1972**, *13*, 3839−3842; (b) Krygowski, T. M. Crystallographic Studies of Inter- and Intramolecular Interactions Reflected in Aromatic Character of π-Electron Systems. *J. Chem. Inf. Model.***1993**, *33*, 70−78.

(9) (a) Noorizadeh, S.; Shakerzadeh, E. Shannon Entropy as a New Measure of Aromaticity, Shannon Aromaticity. *Phys. Chem. Chem. Phys.* **2010**, *12*, 4742−4749; (b) Noorizadeh, S.; Shakerzadeh, E. Aromaticity Study on Tria-, Penta- and Hepta-Fulvene Derivatives. *Comput. Theor. Chem.* **2011**, *964*, 141−147.

(10) Lu, T.; Chen, F. Multiwfn: A Multifunctional Wavefunction Analyzer. *J. Comput. Chem.* **2012**, *33*, 580–592.

S12. Cartesian coordinates (in Å) and electronic energies (E, in Ha) of 1 and 10

All geometries optimized at the B3LYP/cc-pVTZ/SMD level of theory.

1o

E = −1723.3248689 Ha (all vibrational frequencies real)

C	0.245621	0.696862	-0.083858
C	0.245621	-0.696868	0.083817
C	1.477486	1.418144	-0.146920
C	1.477485	-1.418152	0.146879
C	2.635251	0.704509	-0.069623
C	2.635251	-0.704518	0.069574
H	1.454702	-2.491557	0.276271
H	1.454705	2.491551	-0.276306
C	-0.989698	-1.513296	0.217438
C	-1.918176	-1.404903	1.219500
C	-1.277069	-2.600678	-0.677034
C	-0.989697	1.513293	-0.217459
C	-1.277047	2.600673	0.677025
C	-2.408516	3.294799	0.385481
H	-0.652313	2.839649	1.526810
C	-1.918189	1.404914	-1.219507
S	-3.154037	2.623389	-1.039094
C	-2.408533	-3.294802	-0.385462
S	-3.154022	-2.623384	1.039126
H	-0.652353	-2.839663	-1.526829
C	-1.969606	-0.468913	2.382960
H	-0.995041	-0.011990	2.546617
H	-2.691447	0.335669	2.227472
H	-2.251648	-0.990688	3.299234
C	-3.008261	-4.454248	-1.112269
H	-3.106042	-5.332178	-0.469651
H	-4.003483	-4.223015	-1.499024
H	-2.377006	-4.727695	-1.957819
C	-1.969641	0.468941	-2.382980
H	-0.995066	0.012063	-2.546697
H	-2.691437	-0.335675	-2.227463
H	-2.251755	0.990718	-3.299229
C	-3.008227	4.454239	1.112309
H	-2.376950	4.727684	1.957844
H	-3.106028	5.332173	0.469698
H	-4.003438	4.223002	1.499090
C	4.139498	0.705483	-0.071302
C	4.139497	-0.705492	0.071279
C	5.296772	1.432779	-0.145201
C	5.296771	-1.432785	0.145211
H	5.317977	-2.508585	0.254865
C	6.497589	0.688963	-0.069998

C	6.497588	-0.688967	0.070039
H	5.317979	2.508580	-0.254852
H	7.443483	1.211767	-0.123555
H	7.443482	-1.211770	0.123620

1c

E = −1723.2927411 Ha (all vibrational frequencies real)

C	0.823685	1.415622	0.115672
C	2.108498	0.641839	0.421086
C	1.066155	2.826383	0.042254
C	0.823679	-1.415560	-0.115655
C	1.066122	-2.826383	-0.042700
C	2.363331	-3.197865	0.022726
H	0.275264	-3.561980	-0.008693
C	2.108548	-0.641771	-0.420744
S	3.488702	-1.835763	0.001780
C	2.363402	3.197797	-0.023106
S	3.488732	1.835741	-0.001267
H	0.275343	3.562012	0.007754
C	2.181265	0.392365	1.943255
H	1.369118	-0.258541	2.266303
H	3.125407	-0.066950	2.226938
H	2.087194	1.341542	2.468144
C	2.905741	4.581578	-0.120536
H	3.530476	4.823048	0.743421
H	3.532070	4.700551	-1.008537
H	2.095814	5.308528	-0.172673
C	2.181627	-0.392311	-1.942932
H	1.369492	0.258517	-2.266175
H	3.125798	0.067067	-2.226406
H	2.087733	-1.341513	-2.467808
C	2.905695	-4.581672	0.119518
H	2.095794	-5.308701	0.171001
H	3.530697	-4.822590	-0.744398
H	3.531770	-4.701163	1.007631
C	-0.364924	-0.744498	0.008092
C	-0.364922	0.744563	-0.007940
C	-1.630674	-1.457736	0.118209
C	-1.630684	1.457733	-0.118068
C	-2.755495	-0.730422	0.073063
C	-2.755484	0.730392	-0.072811
H	-1.632575	2.535154	-0.200472
H	-1.632412	-2.535152	0.200587
C	-4.236371	-0.706148	0.074166
C	-4.236356	0.706142	-0.073935
C	-5.406125	-1.434062	0.152298
C	-5.406081	1.434073	-0.152341
H	-5.427687	2.509402	-0.266778

C	-6.594063	-0.694421	0.073829
C	-6.594043	0.694444	-0.074110
H	-5.427768	-2.509395	0.266700
H	-7.542838	-1.211953	0.128839
H	-7.542803	1.211983	-0.129322

1ts

E = −1723.2472438 Ha (one imaginary vibrational frequency)

C	0.386686	-0.692011	0.173790
C	0.386686	0.692006	-0.173797
C	1.611692	-1.392980	0.345914
C	1.611691	1.392976	-0.345918
C	2.770234	-0.688828	0.169041
C	2.770233	0.688826	-0.169042
H	1.597528	2.436622	-0.628761
H	1.597529	-2.436625	0.628757
C	-0.882092	1.405825	-0.317565
C	-2.103638	0.713866	-0.695714
C	-1.095635	2.724995	0.120047
C	-0.882092	-1.405830	0.317561
C	-1.095642	-2.724993	-0.120058
C	-2.400105	-3.154857	-0.109037
H	-0.301498	-3.349847	-0.506902
C	-2.103633	-0.713867	0.695723
S	-3.454040	-1.897081	0.506090
C	-2.400096	3.154860	0.109042
S	-3.454044	1.897081	-0.506069
H	-0.301487	3.349852	0.506877
C	4.270730	0.689817	-0.170819
C	5.428676	1.399967	-0.346947
C	6.630772	0.672539	-0.166811
C	6.630773	-0.672542	0.166811
C	5.428676	-1.399970	0.346947
C	4.270729	-0.689820	0.170819
H	5.450597	2.449812	-0.606656
H	7.576690	1.182840	-0.293677
H	7.576689	-1.182843	0.293677
H	5.450596	-2.449815	0.606656
C	-2.947699	-4.451162	-0.596518
H	-3.509884	-4.974494	0.181600
H	-3.628997	-4.308800	-1.440558
H	-2.135444	-5.100746	-0.922413
C	-2.947683	4.451176	0.596486
H	-3.509855	4.974494	-0.181650
H	-3.628997	4.308848	1.440522
H	-2.135420	5.100752	0.922375
C	-2.193145	-0.035918	-2.010795
H	-3.142781	-0.563035	-2.105130

H	-2.114097	0.664988	-2.843363
H	-1.386494	-0.758945	-2.107128
C	-2.193125	0.035918	2.010804
H	-3.142757	0.563040	2.105147
H	-2.114074	-0.664987	2.843373
H	-1.386469	0.758940	2.107130

10o

E = −2139.0502081 Ha (all vibrational frequencies real)

C	0.651299	1.014161	0.255897
C	-0.651296	1.014083	-0.255929
C	1.343630	2.240916	0.488132
C	-1.343780	2.240759	-0.488144
C	0.668292	3.395752	0.236387
C	-0.668579	3.395672	-0.236391
H	-2.357058	2.211851	-0.862902
H	2.356912	2.212126	0.862894
C	-1.388107	-0.221428	-0.621842
C	-0.989141	-1.172983	-1.525335
C	1.388215	-0.221266	0.621861
C	3.227820	-1.482384	0.521918
C	0.989288	-1.172810	1.525384
S	2.243406	-2.367316	1.675584
C	-3.227643	-1.482649	-0.521811
S	-2.243210	-2.367537	-1.675511
C	0.251735	-1.265192	-2.349408
H	0.824406	-0.343098	-2.282180
H	0.897589	-2.081880	-2.020628
H	0.014141	-1.440487	-3.400368
C	-0.251615	-1.265063	2.349413
H	-0.824446	-0.343091	2.281942
H	-0.897291	-2.081953	2.020791
H	-0.014043	-1.440070	3.400428
C	0.668099	4.899463	0.236132
C	-0.668550	4.899383	-0.236183
C	1.357900	6.056827	0.479440
C	-1.358465	6.056663	-0.479567
H	-2.378096	6.077390	-0.839583
C	0.652924	7.256868	0.230495
C	-0.653610	7.256789	-0.230690
H	2.377531	6.077678	0.839449
H	1.148091	8.202982	0.405466
H	-1.148873	8.202844	-0.405714
N	2.642911	-0.406859	0.085479
N	-2.642740	-0.407122	-0.085356
C	4.560745	-1.927778	0.103506
C	5.278814	-1.166891	-0.828482
C	5.143374	-3.094822	0.609004

C	6.541308	-1.566137	-1.238411
H	4.832024	-0.265210	-1.221281
C	6.407178	-3.491138	0.195452
H	4.610862	-3.700528	1.330757
C	7.111877	-2.729191	-0.729753
H	7.083025	-0.967447	-1.958832
H	6.841781	-4.396630	0.597444
H	8.097201	-3.038788	-1.051740
C	-4.560586	-1.928019	-0.103481
C	-5.278303	-1.167675	0.829230
C	-5.143597	-3.094544	-0.609745
C	-6.540789	-1.566963	1.239132
H	-4.831236	-0.266379	1.222600
C	-6.407383	-3.490912	-0.196206
H	-4.611412	-3.699749	-1.332163
C	-7.111714	-2.729522	0.729747
H	-7.082199	-0.968691	1.960131
H	-6.842276	-4.396002	-0.598791
H	-8.097020	-3.039175	1.051728

10c

E = −2139.021162 Ha (all vibrational frequencies real)

C	1.398711	-0.123761	0.159145
C	0.634110	-1.424305	0.434086
C	-1.398736	-0.123771	-0.159239
C	-3.171142	-1.476537	-0.062124
C	-0.634114	-1.424306	-0.434084
S	-1.888432	-2.723386	0.009112
C	3.171144	-1.476514	0.062095
S	1.888375	-2.723407	-0.009014
C	0.360220	-1.528282	1.949114
H	-0.341779	-0.758445	2.269080
H	-0.051787	-2.499987	2.212676
H	1.291210	-1.389963	2.496223
C	-0.360239	-1.528432	-1.949102
H	0.341714	-0.758591	-2.269155
H	0.051820	-2.500141	-2.212566
H	-1.291239	-1.390212	-2.496220
C	-0.739223	1.071020	-0.024396
C	0.739220	1.071010	0.024188
C	-1.467381	2.327253	0.033643
C	1.467400	2.327241	-0.033926
C	-0.733897	3.449922	0.026010
C	0.733924	3.449916	-0.026610
H	2.546830	2.302231	-0.046084
H	-2.546811	2.302263	0.045954
C	-0.709070	4.931012	0.026579
C	0.709120	4.931003	-0.026856

C	-1.441561	6.099955	0.055614
C	1.441640	6.099936	-0.055558
H	2.522134	6.120729	-0.097131
C	-0.697914	7.287309	0.027299
C	0.698023	7.287300	-0.026902
H	-2.522054	6.120764	0.097185
H	-1.217664	8.236192	0.047447
H	1.217796	8.236177	-0.046783
N	-2.758989	-0.253343	-0.130166
N	2.758981	-0.253351	0.130094
C	4.582484	-1.850929	0.015156
C	4.984258	-3.191963	-0.014601
C	5.566635	-0.850601	-0.008468
C	6.330253	-3.524701	-0.067654
H	4.243865	-3.980065	0.012007
C	6.908130	-1.188028	-0.060476
H	5.259725	0.184596	0.013227
C	7.296354	-2.525671	-0.090570
H	6.624090	-4.565487	-0.088892
H	7.656497	-0.406857	-0.078370
H	8.345707	-2.785798	-0.130884
C	-4.582486	-1.850882	-0.015142
C	-4.984310	-3.191919	0.014358
C	-5.566615	-0.850543	0.008812
C	-6.330301	-3.524635	0.067518
H	-4.243943	-3.980034	-0.012613
C	-6.908111	-1.187949	0.060918
H	-5.259690	0.184656	-0.012674
C	-7.296379	-2.525582	0.090785
H	-6.624162	-4.565417	0.088588
H	-7.656438	-0.406744	0.079054
H	-8.345731	-2.785692	0.131202

10ts

E = −2138.9767413 Ha (one imaginary vibrational frequency)

C	-0.659019	1.127225	-0.260330
C	0.658861	1.127343	0.260121
C	-1.343064	2.344228	-0.518493
C	1.342646	2.344471	0.518372
C	-0.663503	3.499409	-0.253637
C	0.662848	3.499530	0.253586
H	2.345067	2.320398	0.920788
H	-2.345486	2.319966	-0.920895
C	1.335986	-0.147877	0.507791
C	0.596946	-1.370449	0.799107
C	-1.335904	-0.148104	-0.508042
C	-3.089758	-1.517583	-0.352366
C	-0.596691	-1.370584	-0.799287

S	-1.823595	-2.675871	-0.780362
C	3.090049	-1.517115	0.352258
S	1.824031	-2.675557	0.780248
C	0.662170	5.000197	0.254128
C	1.346465	6.157489	0.516747
C	0.646685	7.357725	0.248430
C	-0.648137	7.357608	-0.248228
C	-1.347670	6.157244	-0.516623
C	-0.663136	5.000077	-0.254079
H	2.356266	6.178692	0.903464
H	1.137009	8.303897	0.436558
H	-1.138656	8.303690	-0.436295
H	-2.357475	6.178264	-0.903340
C	-0.345285	-1.459185	1.983440
H	-0.868764	-2.414624	2.002694
H	0.220259	-1.366966	2.911325
H	-1.083786	-0.660807	1.963657
C	0.345548	-1.459233	-1.983621
H	0.869214	-2.414570	-2.002808
H	-0.220020	-1.367193	-2.911508
H	1.083892	-0.660708	-1.963901
N	2.644196	-0.275682	0.270696
N	-2.644100	-0.276096	-0.270925
C	4.461735	-1.906343	0.093006
C	4.875238	-3.246862	0.171868
C	5.418737	-0.930891	-0.244524
C	6.193154	-3.598062	-0.078551
H	4.164941	-4.021307	0.430240
C	6.733838	-1.289224	-0.489651
H	5.115670	0.103690	-0.312550
C	7.130376	-2.623286	-0.409494
H	6.489910	-4.636495	-0.013371
H	7.455380	-0.524882	-0.747073
H	8.158353	-2.899112	-0.602886
C	-4.461368	-1.907024	-0.092952
C	-4.874618	-3.247636	-0.171564
C	-5.418539	-0.931695	0.244470
C	-6.192455	-3.599043	0.078981
H	-4.164177	-4.021994	-0.429802
C	-6.733555	-1.290241	0.489732
H	-5.115661	0.102953	0.312301
C	-7.129845	-2.624392	0.409816
H	-6.489014	-4.637545	0.014000
H	-7.455227	-0.525993	0.747069
H	-8.157758	-2.900383	0.603309
